# Subconductance Gating and Voltage Sensitivity of Sarcoplasmic Reticulum K^+^ Channels: A Modeling Approach

**DOI:** 10.1016/j.bpj.2015.06.020

**Published:** 2015-07-21

**Authors:** Antoni Matyjaszkiewicz, Elisa Venturi, Fiona O’Brien, Tsunaki Iida, Miyuki Nishi, Hiroshi Takeshima, Krasimira Tsaneva-Atanasova, Rebecca Sitsapesan

**Affiliations:** 1 Bristol Centre for Complexity Sciences, University of Bristol, Bristol, United Kingdom; 2 Department of Engineering Mathematics, University of Bristol, Bristol, United Kingdom; 3 Department of Pharmacology, University of Oxford, Oxford, United Kingdom; 4 Graduate School of Pharmaceutical Sciences, Kyoto University, Yoshidahonmachi, Sakyo Ward, Kyoto, Japan; 5 College of Engineering, Mathematics and Physical Sciences, University of Exeter, Exeter, United Kingdom

## Abstract

Sarcoplasmic reticulum (SR) K^+^ channels are voltage-regulated channels that are thought to be actively gating when the membrane potential across the SR is close to zero as is expected physiologically. A characteristic of SR K^+^ channels is that they gate to subconductance open states but the relevance of the subconductance events and their contribution to the overall current flowing through the channels at physiological membrane potentials is not known. We have investigated the relationship between subconductance and full conductance openings and developed kinetic models to describe the voltage sensitivity of channel gating. Because there may be two subtypes of SR K^+^ channels (TRIC-A and TRIC-B) present in most tissues, to conduct our study on a homogeneous population of SR K^+^ channels, we incorporated SR vesicles derived from *Tric-a* knockout mice into artificial membranes to examine the remaining SR K^+^ channel (TRIC-B) function. The channels displayed very low open probability (Po) at negative potentials (≤0 mV) and opened predominantly to subconductance open states. Positive holding potentials primarily increased the frequency of subconductance state openings and thereby increased the number of subsequent transitions into the full open state, although a slowing of transitions back to the sublevels was also important. We investigated whether the subconductance gating could arise as an artifact of incomplete resolution of rapid transitions between full open and closed states; however, we were not able to produce a model that could fit the data as well as one that included multiple distinct current amplitudes. Our results suggest that the apparent subconductance openings will provide most of the K^+^ flux when the SR membrane potential is close to zero. The relative contribution played by openings to the full open state would increase if negative charge developed within the SR thus increasing the capacity of the channel to compensate for ionic imbalances.

## Introduction

SR K^+^ channels are selective for monovalent cations and their conduction properties are comprehensively described (1–9). The gating behavior of SR K^+^ channels is less well understood and this is partly due to the complex subconductance state gating of these channels.

There is evidence that there may be two subtypes of SR K^+^ channels termed the trimeric intracellular cation channels (TRIC-A and TRIC-B) (10–12). Mice devoid of both TRIC-A and B are not viable and die in cardiac arrest at embryonic day 10.5 (10) highlighting their necessity in the heart. Cardiac myocytes from TRIC double knockout (DKO) mice exhibit regions of swollen sarcoplasmic reticulum (SR) containing Ca^2+^-deposits that are not present in control mice. At embryonic day 8.5 in DKO cardiac myocytes, spontaneous Ca^2+^ transients were reduced, whereas caffeine-induced release from intracellular stores was enhanced in comparison to controls (10). These observations suggest impaired SR Ca^2+^-release and elevated SR Ca^2+^ levels in the TRIC-DKO cardiac myocytes. The importance of TRIC in intracellular Ca^2+^-movements in other cell types including skeletal muscle (10,13), vascular smooth muscle (14,15), and alveolar epithelial cells (16) is also becoming evident. TRIC-A is expressed at particularly high levels in excitable cells, whereas TRIC-B is expressed at lower levels in all cell types. Although TRIC-B is less abundant, it is essential in some tissues. The *Tric-b* KO mouse dies at birth exhibiting severe ultrastructural abnormalities of the alveolar type II epithelial cells (16). Mutation of the *TRIC-B* gene is also associated with the hereditary bone disease, osteogenesis imperfecta (17–19).

Identifying the physiological roles of the TRIC-B isoform in these tissues requires an understanding of the single-channel properties of TRIC-B and the mechanisms governing channel activation. Because both isoforms are present in most tissues it is difficult to study the single-channel properties of each TRIC channel isoform in isolation. We have therefore made use of the *Tric-a* KO mouse, which can survive to adulthood and thus provides the means for studying the gating of native TRIC-B channels from SR membranes devoid of TRIC-A channels (10). Skeletal SR vesicles isolated from *Tric-a* KO mice were therefore incorporated into artificial bilayers under voltage-clamp conditions to study the interrelationships between channel activation, voltage regulation, and subconductance state gating.

The ability of different kinetic models to reveal the mechanisms underlying the voltage sensitivity and subconductance gating of TRIC-B channels was examined. We find that a simplified, linear, six-state gating model best encompasses the complexity of the subconductance gating behavior of the channel. Our results suggest that subconductance gating in SR K^+^ channels may be more important than previously assumed because ∼75% of the K^+^ flux through the channels at physiological membrane potentials (close to 0 mV) may be due to substate gating.

## Materials and Methods

### Isolation of SR vesicles and bilayer techniques

Light SR (LSR) membrane vesicles were isolated from *Tric-a-*knockout mouse skeletal muscle as previously described (12,20). After incorporation of light SR vesicles into planar phosphatidylethanolamine lipid bilayers, TRIC-B current fluctuations were recorded under voltage-clamp conditions in solutions of 210 mM KPIPES, 10 *μ*M free Ca^2+^, pH 7.2 (12). We used neutral phosphatidylethanolamine membranes to minimize the likelihood of developing changes in surface potential that would affect gating and conductance. By omitting charged lipids such as phosphatidylserine we were able to reduce fusion events and improve the chance of incorporating single K^+^ channels into the bilayer; a prerequisite for analysis of kinetic gating of this channel. The *trans* chamber was held at ground and the *cis* chamber was clamped at various potentials relative to ground. Evidence suggests that SR vesicles incorporate into bilayers in a consistent orientation (21,22) such that the *cis* chamber corresponds to the cytosolic face of the SR channels and the *trans* chamber to the luminal side. The consistent voltage-dependence of TRIC-B also confirms this. To check that only single channels were gating in the bilayer, high positive potentials were applied for short periods of time to maximally activate the channel. Experiments were performed at room temperature (22 ± 2°C).

### Data acquisition, analysis, and simulation

**Figure 1:**
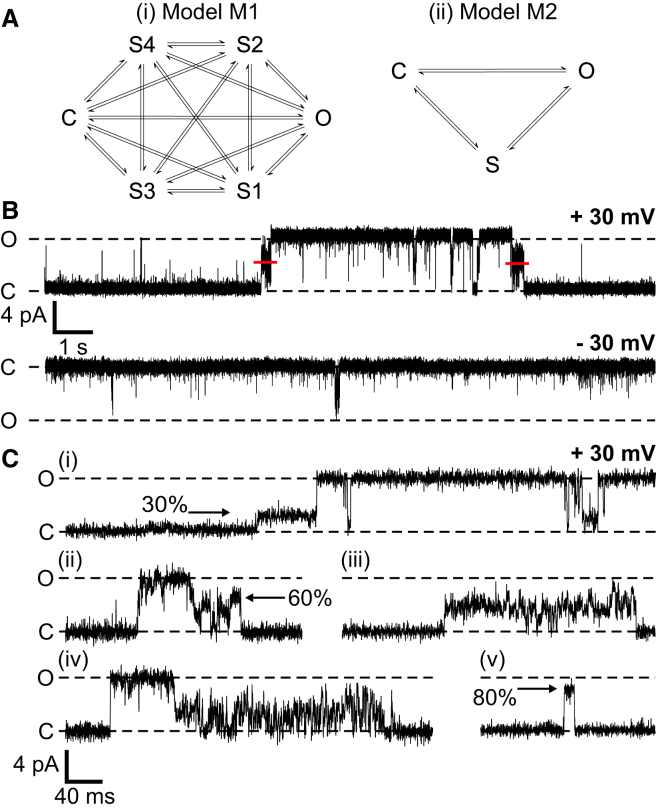
Typical single-channel recordings and gating models. (*A*) Model topologies used in QuB to idealize single-channel recordings. Model M1 (*i*) comprises six states (*full open*, *closed*, and *four subconductance open states* S1–S4 (12)), with all-to-all connectivity between states. Model M2 (*ii*) is a simplified subconductance model consisting of the full open and closed states and a single, merged subconductance state encompassing a noisy combination of all substates S1–S4. (*B*) Representative single-channel recordings illustrating typical opening bursts at +30 and −30 mV. (*C*) Expanded traces show typical subconductance state open events. The red lines show the average current level of the merged subconductance state. To see this figure in color, go online.

Single-channel recordings were digitized at 100 kHz and recorded on a computer hard drive using WinEDR 3.05 software (John Dempster, Strathclyde University, Glasgow, UK). Before idealization, traces were digitally filtered (Gaussian Filter with cut-off frequency of 1 kHz) and resampled at 10 kHz. Single-channel current fluctuations were idealized using the segmental k-means algorithm (23) in the QuB software suite (State University of New York, Buffalo, NY). Idealization was initially obtained using two Markov models, M1 and M2 (Fig. 1 *A*). For M1, a six-state Markov model was constructed with identical fully connected topology (see Fig. 1 *Ai*), where states consisted of the closed, open, and four subconductance states using our initial estimates of the current amplitudes of the conductance states (S4 = 60 pS, S3 = 93 pS, S2 = 121 pS, S1 = 161 pS, Full = 199 pS) as defined previously (12). For M2, the four subconductance states, S1–S4, from M1 were merged together into a single conductance class of amplitude midway between the fully closed and fully open states, with noise level (standard deviation in QuB) equal to half this amplitude (see Fig. 1 *Aii*). Open probability (Po) was measured from recordings of duration 1–3 min using model M2 except where use of model M1 is specified. Po in each state (including subconductance states) was defined as the proportion of the total duration of idealized experimental recording spent in that state.

Mean currents were computed using QuB: each trace was idealized using model M2, and mean current amplitude in each state was computed directly from underlying experimental recordings. Computational details about simulations of mean currents from models are provided in the Supporting Material.

Boltzmann curve fits were computed in GraphPad Prism 4.02 (GraphPad Software) through nonlinear least squares optimization. Transition frequencies were visualized as chord diagrams, plotted using the Circos software package (24). Lifetime distributions were computed from QuB idealizations where only a single channel was gating in the bilayer. Unless specified otherwise, events shorter than 0.6 ms were stripped from the idealized event sequences in QuB, or automatically merged where appropriate (25,26). Individual time constants were fitted with an exponential log probability density function (pdf) in Clampfit, using maximum-likelihood fitting (27) to determine the expected number of time constants for each conductance state. The optimal number of time constants for each distribution was determined using a log-likelihood ratio test (28) at a confidence level of P = 0.95. Missed events during maximum interval likelihood (MIL) rate optimization were automatically accounted for in QuB by computation of a corrected Q matrix in the MIL algorithm used to obtain kinetic rates.

Individual sets of kinetic rates for different models were obtained by fitting idealized recordings from experiments where only a single voltage-dependent channel was gating in the bilayer using the MIL algorithm implemented in QuB (25,26). We additionally used the global fitting capabilities of QuB (25,26) whereby rates are simultaneously fit across a set of idealized experimental recordings to obtain the optimal rates, henceforth referred to as global rates. Monte Carlo simulations of the models were performed with our own codes written in Python and C, using the Direct Method of the stochastic simulation algorithm (Gillespie’s Algorithm (29)). For each model, traces were simulated until the stationary amplitude distribution converged (see Supporting Material for further details).

An example of a fast-gating model was also considered in which the transitions between the full open and full closed channel levels are so rapid that subconductance state events arise as an artifact of filtering the data. For the fast-gating model, simulated recordings were filtered at 10 kHz and resampled at 100 kHz to approximate the data acquisition and digitization process. At this stage, simulated Gaussian noise of equal magnitude to that observed in experimental recordings was added to the resampled simulated recordings, which were then filtered again at 1 kHz and resampled at 10 kHz to replicate the properties of physically acquired traces at the analysis stage.

## Results

### TRIC-B channel gating behavior

TRIC-B channel gating is characterized by a number of prominent features (Fig. 1, *B* and *C*). These include: 1) voltage-dependent opening, 2) trains of subconductance events before and after a full opening burst, and 3) gating to subconductance open states without visiting the fully open state. Heavy filtering and compression of recordings as in (Fig. 1 *B*), top trace, can suggest that there is a single subconductance level at ∼53% of the full open state. However, time-based expansion of traces shows that the channel can dwell in what appear to be multiple distinct sublevels (examples shown in Fig. 1 *Ci*, *ii*, and *v*). The channel also appears to gate in prolonged sublevels without fully opening (Fig. 1 *Ciii*). We have previously shown that TRIC-B appears to gate in four main subconductance open states (12), however, the variable trains of subconductance state events are difficult to quantify because the individual events can be very brief (see Fig. 1 *Ciii* and *iv*). We therefore evaluated two different levels of model complexity as a means to idealize the data (Fig. 1 *A*) and assess the contribution of subconductance state gating to the overall Po and current fluxes through the channel. Model M1 (Fig. 1 *Ai*) comprises six distinct conductance levels, corresponding to the previously reported (12), closed, full open, and four distinct subconductances (S1, S2, S3, and S4). Model M2 (Fig. 1 *Aii*) combines all subconductance openings into a single merged class. Model suitability was assessed using eight representative single-channel experiments. Further details are given in the Supporting Material.

**Figure 2:**
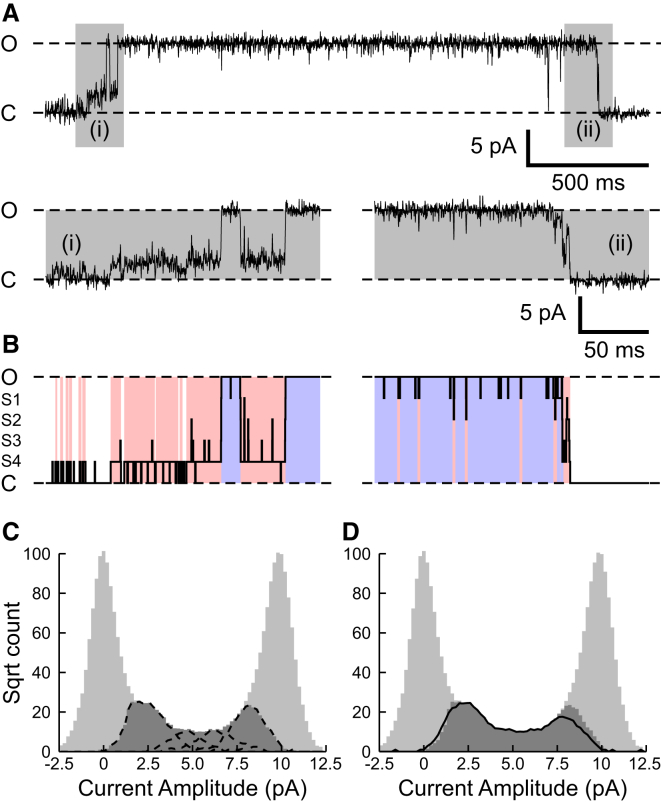
Comparing idealization of single-channel recordings using models M1 and M2. The trace in (*A*) shows a representative opening channel burst at +50 mV. Gray areas inset underneath show typical opening (*i*), and closing (*ii*), subconductance gating on an expanded timescale. Dashed lines, labeled C and O, indicate closed and full open levels, respectively. (*B*) Comparison of idealization of the opening (*left*) and closing (*right*) sequences shown directly above (*Ai* and *ii*), computed using models M1 and M2. Amplitudes labeled S1–S4 indicate approximate amplitudes of the four measured subconductance states. Solid black lines in (*B*) show idealization obtained using model M1. Idealization of the same data using model M2 is represented by colored block regions, where pink regions are classified as subconductance state and blue regions as fully open states. The amplitude histograms compare data points from the entire recording from which burst (*A*) was taken (180 s), idealized using either model M1 (*C*) or M2 (*D*). Light gray bars show the all-points amplitude distribution for the entire recording. Dark gray bars correspond to the amplitude distribution of all points in the recording classified as any one of subconductance states S1–S4 when idealized using model M1. The dashed lines in (*C*) show the amplitude distributions of data points classified as subconductance states in model M1. The solid line in (*D*) shows the amplitude distribution of subconductance states classified by model M2. To see this figure in color, go online.

Fig. 2 depicts a comparison of the idealization and event classification obtained by models M1 and M2 for a single typical opening burst (Fig. 2 *A*) to the full open state. The inset traces underneath show expanded views of the trains of subconductance transitions that occur before and after the sojourn in the full open state. Idealization of the expanded sections of recording shown in Fig. 2A*i* and *ii*, using model M1, are illustrated in (Fig. 2 *B*) as solid black lines. Model M1 states, and transitions in the idealization, obey a close correspondence with those observed in the recording and to idealization by model M2 (*colored background regions*). Events classified as S (*pink regions*) by model M2 may belong to any of the subconductance states classified as S1–S4 by model M1 (or to additional subconductance states). Events classified as fully open are represented by blue regions. Crucially, the idealization produced by model M2 captures the majority of subconductance state gating behavior, distinguishing it from dwell times in the closed or fully open states. Durations of medium to long subconductance events were conserved between models M1 and M2 but the example (Fig. 2) shows that a small number of brief S1 and S4 transitions that are captured by M1, are not classified as substates by M2. Such discrepancies were rare when the recordings from all the channels were analyzed and their distributions compared (see Supporting Material for further analysis).

Conservation of subconductance state classified events between models M1 and M2 is further demonstrated in the all-points amplitude histograms for the recording shown in Fig. 2 *A* (Fig. 2, *C* and *D*). The dark gray areas highlight all points classified as subconductance states S1–S4 by model M1. Dashed lines in Fig. 2 *C* indicate the underlying amplitude distributions of currents classified as the distinct subconductance states S1–S4 by M1. In Fig. 2 *D*, the solid line illustrates the subconductance state amplitude distribution resulting from idealization using model M2. This closely matches the profile of the amplitudes identified by M1, however, this is at the cost of missing some brief subconductance events with durations close to the minimum resolvable duration.

Because model M2 correctly assigns events as open, closed, or subconductance, we used this simplified model to idealize recordings to examine the stability of gating over time. Channel Po fluctuated markedly because the randomly occurring full opening bursts were widely dispersed at all voltages as can be seen in the example Po Diary Plots shown in Fig. S2. Averaging the plots for 17 experiments (Fig. S2) provided no evidence for inactivation with time. Channels were predominantly closed at −30 mV and any openings were usually to subconductance states with rare full open events.

**Figure 3:**
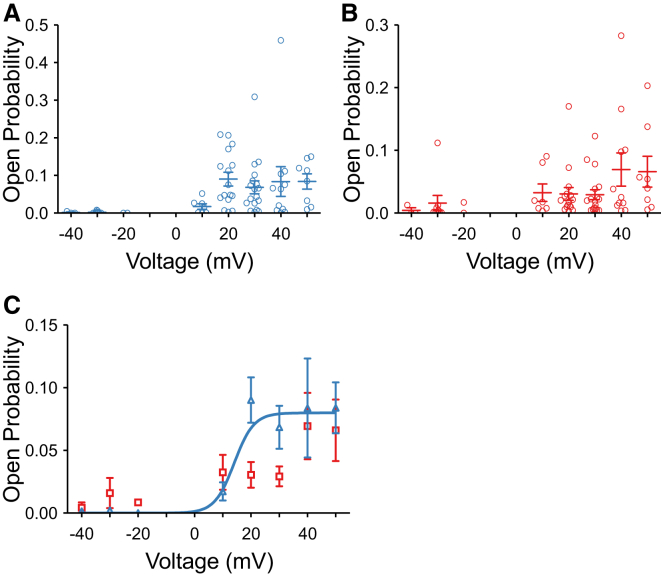
Po-voltage relationships. Po for full (*A*) and subconductance (*B*) openings as a function of voltage is shown for individual experiments. Model M2 was used to idealize the data. In (*C*), the mean data is shown for subconductance Po (*squares*) and full open state Po (*triangles*). The line corresponds to a Boltzmann fit to the Po in the full open state according to the equation:$Po\left(V\right)={\text{Po}}_{\text{max}}{\left\{1+\text{exp}\left(\left[\Delta G+zFV\right]/RT\right)\right\}}^{-1},$ where Δ*G* is the internal free energy of opening, *z* is the effective gating charge, Po_max_ is the maximum Po, and *F*, *R*, and *T* have their usual meanings (fit parameters: Po_max_ = 0.08 ± 0.01, Δ*G =* 9.9 ± 1.3 kJ mol^−1^, *z* = −7.3 ± 0.9). The negative *z* value indicates that negative charge moves in the *trans* (*luminal*) to *cis* (*cytosolic*) direction or that positive gating charge moves in the opposite direction as voltage increments from 0 to +20 mV. Mean values ± SE are shown. The following number of recordings were used at each voltage: −40 mV (3), −30 mV (8), −20 mV (2), 10 mV (7), 20 mV (12), 30 mV (17), 40 mV (10), and 50 mV (8). To see this figure in color, go online.

We investigated Po variation over a range of voltages for experiments where only a single channel was gating in the bilayer (Fig. 3). Approximately 11% of channels gate in a voltage-independent manner (12) and these channels were not included in the Po-voltage plots. Model M2 was used to idealize recordings to compare the voltage-dependence of opening to the full (Fig. 3 *A*) and the subconductance states (Fig. 3 *B*). In Fig. 3 *C*, a comparison of the mean data for full openings and subconductance states is shown. The figure illustrates large variability in Po across the channels but shows that voltage-dependent TRIC-B channels exhibit very low Po at negative potentials and predominantly subconductance events. There is a steep relationship between Po and voltage between +10 and +20 mV and thereafter Po plateaus. The critical voltage activation range for subconductance gating appears similar to that for full conductance openings and may even occur at slightly lower voltages (0 mV to +10 mV) but this is difficult to determine because of low Po, variable channel behavior, and the difficulties of resolving small amplitude subconductance events at voltages <10 mV.

### Lifetime analysis

**Figure 4:**
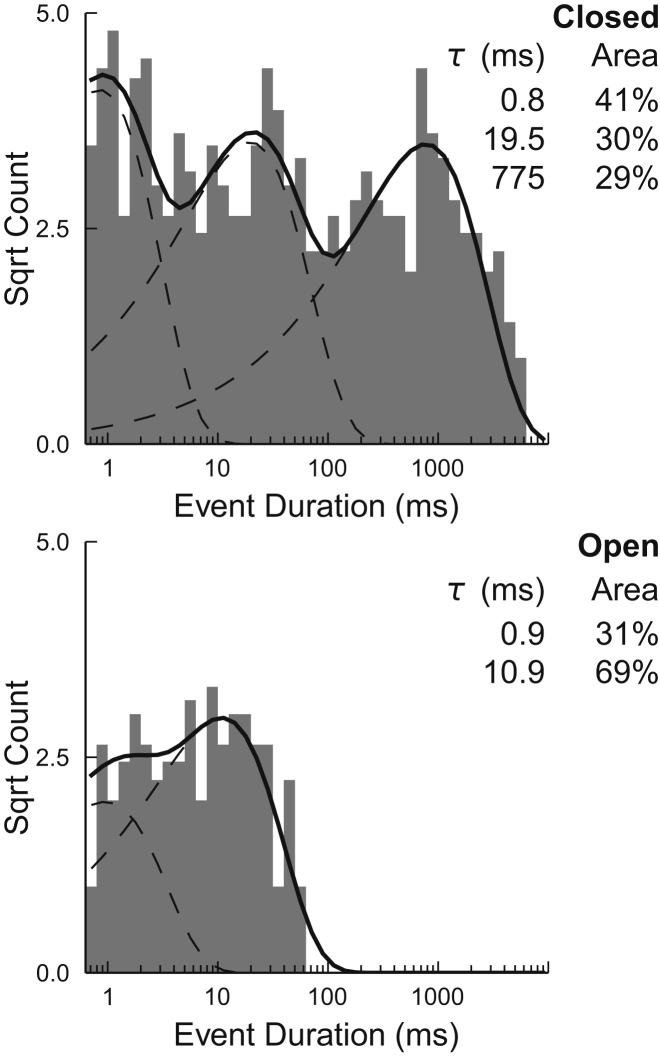
Representative open and closed lifetime distributions. Representative lifetime distributions are shown for events from a single-channel recording, idealized using model M1. Histograms of lifetime distributions (*gray bars*) are shown for the closed (*upper*) and full open state (*lower*). Durations are plotted on a logarithmic scale (10 bins per decade) and the square root of the event count is shown. Fitted time constants and their respective areas are shown for each state. The dashed lines show pdfs of individual time constants, and the solid lines show the total fitted distribution for each state. Table S1 details the data from eight experiments where only a single voltage-dependent channel was gating.

Model M1 was used to assess the lifetime durations of the open, closed, and subconductance states of voltage-dependent single channels at +30 mV. At negative potentials, there were too few events for analysis. Lifetime distributions and pdfs from a typical channel for the full open and closed states are shown in Fig. 4. In this example, the best fit to the data was obtained with three closed and two open states, however, four closed states and three open states were observed in some experiments. The time constants and percentage areas for the lifetime distributions from eight representative voltage-dependent single-channel experiments are tabulated in Table S1. The mean dwell times for subconductance open states, S1, S2, S3, and S4 were 1.7 ± 0.1, 2.3 ± 0.2, 2.1 ± 0.2, and 1.9 ± 0.4 ms, respectively (SE; *n* = 8). Attempts to apply lifetime analysis to the subconductance dwell times indicated that multiple tau components may be required to describe the lifetime distributions. However, because all components were very close to or below the minimum resolvable event duration, this level of detail was inappropriate for our current limits of resolution.

The multiple components required to describe the full open and closed states plus the subconductance events indicates that a high level of model complexity is required to capture channel kinetics. Certain gating motifs, however, were observed with every channel. Transitions to and from the full open and closed states almost always appear to pass through a subconductance state or a train of subconductance events (see Figs. 1 and 2). Similar findings have been reported previously for native frog skeletal SR K^+^ channels (2). We found only a handful of events (see Table S2) that appeared not to transition through subconductance states before entering full open or closed states. With these rare events, if the filter cut-off frequency was increased, the suggestion of a sublevel event could be observed indicating that we may simply be missing subconductance events due to inadequate resolution. Fig. S1 shows an example of such an event.

**Figure 5:**
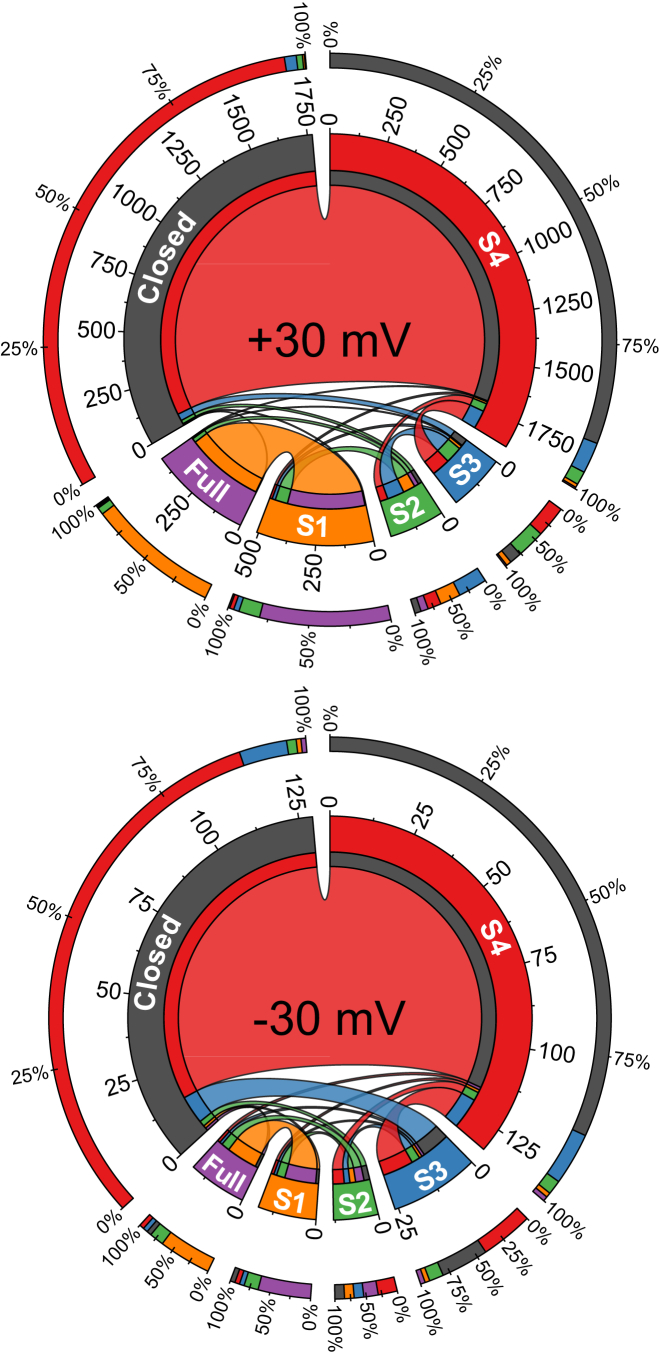
Mean transition frequencies between states for voltage-dependent channels. The chord diagrams show the preferred transition routes through the full open, closed, and subconductance states, S1–S4, that are observed at ±30 mV. The mean data for eight voltage-dependent channels is shown. The transition counts are displayed around the central circle where the labeled segments correspond to each of the six states in model M1. States are colored as follows: Closed, gray; S4, red; S3, blue; S2, green; S1, orange; full, magenta. The inner rings show the proportion of transitions out of a state to the other states in the model. Ribbons between pairs of state segments correspond to transitions between the connected states, with ribbon width at a state segment corresponding to the number of transitions out of that state. Outer rings show percentage contributions of each state to the total incoming transitions into any state segment. Note that at −30 mV, there are at least 10 times fewer transitions overall than at +30 mV. At both potentials, a much greater proportion of transitions occur between S4 and the closed state than between any other states, however, at +30 mV, more transitions from S3 and S2 are to S1, S2 or the full open state. To see this figure in color, go online.

The transition frequencies between all states were examined to determine if there were any dominant transition routes. Chord diagrams that provide a visual means of identifying the preferred transition routes into and out of the various states at ±30 are shown in Fig. 5. The diagrams depict the mean data from eight channels and emphasize that there are at least 10 times fewer transitions from each state at −30 mV than at +30 mV. At both −30 mV and +30 mV the most frequent transitions are between the closed and S4 states. Note also that, at −30 mV, a large proportion of transitions from S3 also drop back to the closed state, whereas, at +30 mV, transitions from S3 to S2 are more frequent. At +30 mV, there is also a shift to a greater number of transitions between the full open and S1 states.

### Kinetic gating models

**Figure 6:**
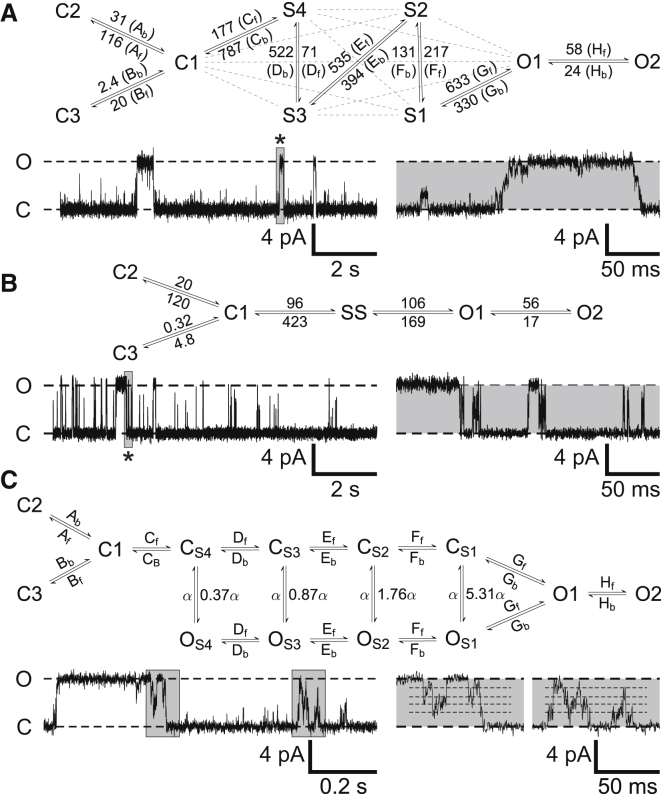
Model simulations. (*A*) Model M1 after infrequent transition routes are excluded (M1-reduced). The model is adapted by adding two further closed states (C2, C3) and an extra open state (O2), to reflect the minimum number of closed and open time constants obtained from lifetime analysis. The number of Markov states for each individual subconductance state S1–S4 was kept constant at one. The model scheme is shown with only the transitions whose fitted rates were consistently larger than a threshold of 10^−3^ s^−1^, across *n* = 8 voltage-dependent channels fitted at +30 mV. The all-to-all connectivity reduces to a linear scheme when connections that are not consistently frequent across all traces are discarded. Discarded connections are shown as gray dotted lines. These transitions did sometimes occur across the eight traces, but were not consistently present in all the channels (for example, see Table S4). Rates were obtained through a simultaneous global fit of the simplified linear scheme (M1-reduced) to eight channel recordings in QuB. These rates (s^−1^) are given next to their corresponding connections, and are labeled A through H to correspond to the equivalently labeled rates shown in (*C*). An example of simulated openings is shown, and the asterisk indicates where an opening burst highlighted in gray is shown on an expanded timescale (*right*) to show typical features. (*B*) Model M2 after adaptation (M2-adapted) to include the same number of full open and closed states as the M1-reduced model (*above*). It was assumed that transitions O–C occur too infrequently to be included. Rates shown are from a simultaneous global fit in QuB to the data from eight voltage-dependent channels. An example of the model output is shown and the asterisk indicates where an opening burst highlighted in gray is shown on an expanded timescale (*right*) to show typical features. Time and current scales are equivalent to those in (*A*). The simulated traces exhibit superficial similarity to real recordings; however, the model produces a smooth range of subconductance amplitudes around a common mean unlike the subconductance events in experimental recordings. This is obvious in the amplitude histograms of the simulated data (*black line*) shown in Fig. S6. (*C*) Topology of a candidate fast gating scheme, which does not include any explicitly defined subconductance states and only contains fully open (*O*) or fully closed states (*C*). Rapid transitions between coupled pairs of closed and full open states labeled C_S4_/O_S4_, C_S3_/O_S3_, C_S2_/O_S2_, and C_S1_/O_S1_, result in observed subconductance state events of amplitude S4, S3, S2, and S1, respectively, after simulated events are low-pass filtered and resampled. Flickery gating arises from rates labeled *α*, where *α* is a fast rate (e.g., 50,000 s^−1^). Transition rates between all remaining states are equivalent to those used in the M1-reduced model and are labeled A–H next to the transitions. A sample of the model output is shown with insets to the right showing subconductance gating on an expanded timescale. Again, amplitude histograms of the simulated data correspond well to experimental recordings (Fig. S7).

A pattern emerged that was true for both positive and negative voltages: transitions out of any state appear to lead to states that are closest in current amplitude to that state. For example, from S2, the most likely transition route is to S1 or S3. From S3, the channel is three times more likely to enter S4 or S2 rather than any other state. At −30 mV, there were too few events to examine the transitions any further. In four of the eight experiments used, there were no openings at −30 mV. At +30 mV, however, we can modify model M1 (Fig. 1) to incorporate the fact that we observe at least three closed states and at least two full open states (Fig. S3), and then take into account the likelihood of the various connections between states (see Fig. 6 *A*). In this reduced, linear gating M1 model (henceforth termed M1-reduced), the solid lines show only those transitions that were consistently observed in all eight channels and therefore highlight the preferred transition routes. Examples of single-channel traces simulated by the M1-reduced model are shown in Fig. 6 *A* to illustrate typical features and show agreement with experimental data. The amplitude distributions obtained from simulations using rate constants derived from individual recorded channels closely matches the experimental data (Fig. S5).

### Alternative mechanisms for observed substate gating

We considered that the subconductance events could be artifacts arising from filtering rapid transitions between full open and closed states. Thus, if events are too brief to resolve, a burst of openings may then appear as a subconductance event, the current amplitude of which will depend on open and closed rates. Similar suggestions have been made for various K^+^ channels exhibiting subconductance openings (6,30–34). We constructed a model example of a fast-gating mechanism that includes no explicitly defined subconductance states (see Fig. 6 *C*) and examined whether such a model was capable of generating currents that exhibit qualitatively similar sublevels to TRIC-B. The rates were adjusted to generate subconductance states of similar amplitude to the experimentally observed amplitudes, S1–S4. These closed and open states are labeled C_S1–S4_ and O_S1–S4_ according to the amplitude of the subconductance events that are observed following low-pass filtering and resampling (as for the experimental data). In this candidate model, the closing rate, *α*, is chosen to be 50,000 s^−1^ from O_s1_, O_s2_, O_s3_, and O_s4_ (but these rates could be varied). As in real recordings, full transitions to either the open or closed state were not directly observed in the model output, and instead manifested themselves as small, fast, and noisy subconductance bursts (similar to S1 and S4 behavior in model M1).

Fig. 6 *C* shows a sample of the fast-gating model output highlighting that this model could also reproduce the frequent brief subconductance events that do not subsequently lead to full openings. When the simulated data are analyzed in QuB according to idealization scheme M1 (as for the experimental data), amplitude histograms are generated exhibiting distributions similar to those obtained from experiment (Fig. S7).

### Discerning the best model

Model M2 idealized data without severely misclassifying events as sublevel, full open, or closed states (Fig. 2). Lifetimes of the events classified as closed, sublevel, and full open states resulting from M1 or M2 were also similar (Fig. S9). However, simulations using model M2-adapted (Fig. 6 *B*) do not mimic the experimental single-channel current fluctuations as well as the M1-reduced (Fig. 6 *A*) or fast-gating (Fig. 6 *C*) models because M2 cannot account for variations in the subconductance states (Fig. 5) that are a feature of the experimental data. The average full and subconductance currents corresponding to the mean overall fluxes in these states also provide a reasonable approximation of the data. Model M2 is, therefore, ideal for a coarse-grained analysis of TRIC-B Po, for simulation of mean currents, and rates of transitions between the merged subconductance state and the open and closed states but cannot reproduce the intricacies of TRIC-B subconductance gating.

Simulations of the M1-adapted, M1-reduced, and the fast-gating models all produced single-channel current fluctuations that appeared very similar to the experimental data. Average current distributions obtained from simulations using rate constants derived individually from each experimental trace also closely matched the experimental data very well for most channels (see Figs. S4–S7 and Table S3). The Earth Mover's Distance (EMD) (35) was used as an additional measure of the difference (distance) between experimental and simulated amplitude distributions corresponding to the work (total change in area) required to change the model distribution to exactly match experimental data. Our analysis showed that the output of the M1-reduced model was very similar to that of the M1-adapted model, which maintains all connections between various states. This indicates that, although we may lose some detailed kinetics that are not fully resolvable after data filtering and acquisition, simplification to the M1-reduced model effectively encapsulates key aspects of the gating kinetics. It is likely that states S1–S4 exhibit more than one tau component in their lifetime distributions, although we cannot accurately determine these brief time constants. To better reproduce the full gating kinetics, any model would require more than one Markov state per subconductance.

The fast-gating model yields slightly higher EMD values to those obtained for the M1-reduced and M1-adapted models suggesting that it replicates the data slightly less effectively, especially when simulating currents using globally fitted rates (Table S3). Thus, the large variation in transition rates between channels causes difficulties for producing a one-model-fits-all without further resolution of the time constants in the experimental data. Open channel noise in the fast-gating model is determined by the fast rate *α* and the imposed level of filtering, constructed from a model of the baseline experimental noise, and is kept constant for all simulations. Baseline noise was adjusted to match individual recordings as in all simulations of our other models. This was constant for all fast-gating model simulations; however, when combined with the intrinsic open channel noise arising from fast gating, the model overestimated substate Po for globally fitted rates. As a result, the fast-gating model does not fit experimental recordings with higher open-channel noise as well as the other models, in which per state open-channel noise distributions are explicitly specified in QuB. We did compare the substate noise observed in experimental recordings to the noise resulting from a range of filtering levels applied to substates in the fast-gating model (Fig. S11) showing that it is unable to completely reproduce the exact noise characteristics observed experimentally.

EMD values for each model were further used with multidimensional scaling (MDS) to compare the performance of each model in relation to the data. MDS is an established tool for visualizing relationships between data by dimensionality reduction (36). The details of our analysis are described in the Supporting Material. Fig. S10 shows how the results of the EMD analysis of TRIC-B gating can be transformed using MDS by extending pair-wise analysis of EMDs between the amplitude histograms observed in experiments and those obtained by model simulation. Fig. S10 shows that differences between the extended M1-adapted and the linear M1-reduced models are negligible and that these models are closely aligned around the mean of the experimental data. This emphasizes further that the M1-reduced model contains all the important transitions required to provide a comprehensive working model of TRIC-B at +30 mV. The M2 model and the fast-gating models are clearly offset further away from the experimental mean indicating that both provide inferior descriptions of the data to the M1-reduced model.

### Voltage-dependent transitions

**Figure 7:**
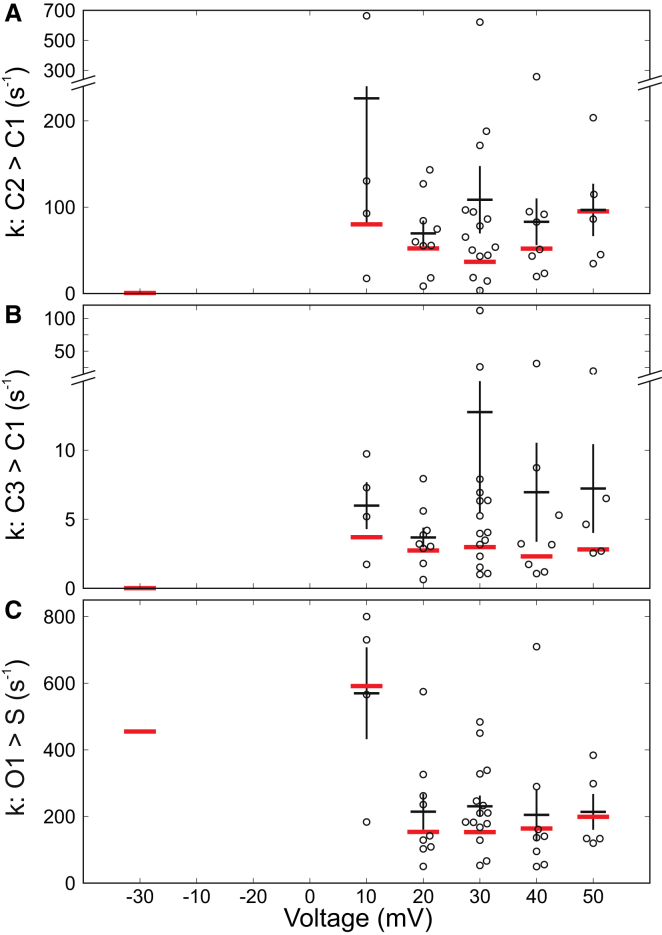
Voltage-dependent rates in model M2. Rates were fitted across voltages from −30 to +50 mV, using MIL as implemented in QuB. The graphs of the three rates that appear voltage sensitive (C2 > C1, C3 > C1, and O1 > S) are shown in (*A*), (*B*), and (*C*), respectively. Individual recordings at each voltage (*black circles*) are shown and the horizontal black bars are the mean rates at each voltage (SE). The global fit, where all recordings obtained under a given condition were fitted simultaneously (also in QuB), are plotted as red bars. At −30 mV, only four recordings were available with openings. The number of openings was insufficient to fit rates for individual recordings, therefore global fits only are shown at −30 mV. The following number of recordings were used at each voltage: −30 mV (4), 10 mV (4), 20 mV (9), 30 mV (15), 40 mV (8), and 50 mV (5). To see this figure in color, go online.

There are two challenges to extracting the voltage-sensitive pathways. First, the single-channel events are poorly resolved over the voltage range where TRIC-B gating is most sensitive (0–20 mV) and therefore transitions between substates cannot be described. For this reason, model M2 must be used to investigate the main voltage-sensitive steps leading into and out of the subconductance levels. Second, at negative voltages, very few events are observed. Therefore, if one or two long events were observed, this could markedly bias the calculation of transition rates at −30 mV. With these caveats in mind, rates in model M2 were fitted to a full range of experimental data, across voltages from −30 mV to +50 mV, using MIL as implemented in QuB. Too few events occurred at −30 mV to obtain meaningful average rate constants and so only the global fit to the data is shown at this voltage. We found three rates that appeared voltage sensitive and these are shown in Fig. 7. An increase in the rate of transitions from C3 and C2 into C1 when voltage switches from −30 mV to positive potentials was observed. We expect that this would reduce the number of long closings and be the main cause of the increase in frequency of openings. Transition rates C2 > C1 and C3 > C1 will be greatly overestimated at −30 mV because experiments where there are no openings at −30 mV cannot be included thus emphasizing the strong voltage-dependence of these transitions. The transition O1 > S was also sensitive to voltage and was slowed at voltages ≥+20 mV. This would lead to longer full openings above +10 mV. It is likely that additional voltage-sensitive transitions would be revealed if there were not so few openings at −30 mV.

### Voltage-dependence of full and subconductance currents

**Figure 8:**
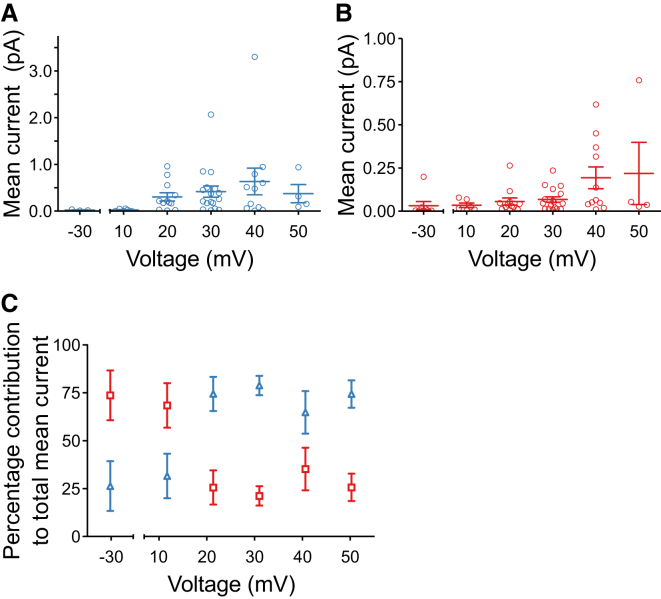
Contribution of the full open and subconductance states to the overall current flowing through the channel. The relationship between mean current and voltage is shown for the full open state (*A*), and for openings to any subconductance level (*B*). Mean current contributions due to full and subconductance open states for individual experiments are plotted as open circles. Mean values ± SE are shown (*n* = 8 (−30 mV), 7 (10 mV), 12 (20 mV), 17 (30 mV), 10 (40 mV), and 4 (50 mV)). Percentage contributions of the full open and combined subconductance states to the total current were then computed for each experiment and were averaged across individual experiments at the various holding potentials (*C*). To see this figure in color, go online.

To investigate if subconductance gating of TRIC-B would contribute significantly to physiological K^+^ currents across the SR or whether such events would be trivial in comparison to the full openings, we calculated the mean currents resulting from gating in subconductance or full open states. Fig. 8 compares the mean single-channel full open state current (Fig. 8 *A*) and the mean single-channel subconductance state current (Fig. 8 *B*) as a function of holding potential for those experiments where only a single channel was gating in the bilayer (so that openings to subconductance states could be correctly assigned). In Fig. 8 *C*, the percentage contribution of subconductance and full open states to the total current is shown as a function of membrane potential. Extrapolation to physiologically relevant holding potentials (near 0 mV) indicates that subconductance gating of TRIC-B will be responsible for most of the current (∼75%).

## Discussion

We show that the gating of SR K^+^ channels is extremely variable from channel to channel but that the main characteristics can be described in a simplified linear gating model that includes four subconducting open states. The Po in the full open state is steeply dependent on voltage over the range 0 to +20 mV but subconductance Po does not entirely mirror this relationship. At +10 mV, the probability of the channels dwelling in a sublevel is still higher than that of opening to the full open state but this is reversed at +20 mV. In terms of the mean K^+^ flux through each channel, most of the current (∼75%) will be due to sublevel openings at ≤+10 mV but at ≥+20 mV, ∼75% of current will be due to full open events.

Subconductance openings are especially apparent at −30 mV where the channel opens predominantly to lower amplitude sublevels (S3 and S4). At +30 mV, not only are there more of these low amplitude openings and more full openings but the proportion of openings to higher amplitudes is also increased. Fig. 3, *B* and *C*, suggests that subconductance activity is sensitive to voltage in the range 0 to +10 mV (but current amplitudes are too small to clearly discern sublevels at <+10 mV), whereas voltage change between +10 mV and +20 mV produces the greatest increase in the probability of opening to the full open state. This suggests that a greater amount of transmembrane charge movement is required for TRIC-B to open to the full open state than is necessary for the channel to open to sublevels only.

Subconductance openings in certain voltage-dependent tetrameric ion channels have been suggested to arise from the distinct, noninstantaneous movements of individual subunits of the channel (30,37) under voltage. The movement of the individual subunits gives rise to conformational changes within the conduction pathway such that a full conductance opening is only possible when all four subunits have moved. For TRIC-B, a trimeric protein, such a neat explanation of voltage-dependence would require observation of just two clear sublevels. We observe more apparent sublevels and so can only make loose analogy, but because higher positive holding potentials favor full open states and S1 rather than the lower sublevel openings, the sublevels could represent the incompletely synchronized movements of different subunits or different regions of the channel as it is coerced to open with positive holding potentials. Because open-closed transitions occur via sublevels in 99.99% of transitions, and as transitions between apparent sublevels are very fast (for example, Fig. 1 *Ciii*), we considered that the subconductance events could arise due to very rapid, incompletely resolved transitions between the closed and full open state. The ionic selectivity of the full open and the noisy sublevel of various SR K^+^ channels (data generally filtered at ∼100 Hz) is similar for a range of monovalent cations (3,5,38,39) which would support this theory. Block of the rabbit cardiac SR K^+^ channel by succinyl choline also pointed toward a gating mechanism to explain the subconductance behavior because the degree of block was reported to be greater for the full open state than for the noisy subconductance state (4). This would be expected if the sublevel was caused by rapid open and closed transitions because the lower Po of the sublevel would reduce the likelihood of a pore blocker entering the conduction pathway. However, in these earlier experiments the recordings were filtered at 100 Hz and hence a single noisy subconductance level only was observed. Only gross changes in subconductance amplitude or duration could be detected under these conditions and so these experiments do not solve this issue. Changing temperature also did not provide clear insight into the underlying mechanisms (8). Over the range 16–37°C, no change in the relative amplitude of the substate to full open state was detected, nor any obvious differences in the durations of time spent in the full open state relative to the subconductance state (8).

In the absence of compelling evidence for the underlying structural changes that lead to subconductance events, we compared the ability of different gating models to portray the interdependence of the subconductance and full open states. Our candidate fast gating model provided a convincing mimic of experimental single-channel recordings but the amplitude histograms, EMD analysis (Table S3 and Figs. S4–S8) and the use of MDS (Fig. S10) showed greater deviation from experimental data than the linear M1-reduced model that retains four subconductance open states. Slight improvements to the fast-gating model should be possible by fine-tuning the fast rate *α*, by combining the gating scheme with a more realistic model of open channel noise, or by the inclusion of more open and closed states that are not observed visually. Our attempts at adjusting the fast rate *α* or the noise characteristics (Fig. S11) did not achieve significant improvements to the model output and so our best fit to the data at +30 mV remains the linear M1-reduced model.

It is important to note that the computed EMD values between each model and its corresponding experimental data were lower than those observed within the experimental data (Table S3). In other words, when the mean EMD for each experimental recording compared to the other five experiments was calculated, the EMD values ranged from 0.355 to 0.417, which are much higher than the EMD between any of the models and the data. This interchannel level of complexity reduces the likelihood of converging to a gating scheme that accurately describes all aspects of TRIC-B gating. Nonetheless, we show that the M2 model provides a good approximation of transitions between the merged subconductance state and the full open and closed states. The linear M1-reduced model enables integration of the most frequent transitions between subconducting open states, S1–S4, into the model and provides significantly improved simulation of experimental data. It is clear from global fitting of model M2 that important voltage-dependent rates are C3 > C1, C2 > C1 and O1 >S. These transitions are a key element of the close correspondence between the linear gating simplification of model M1 and experimental data. However, simplified substate transitions in M1-reduced are drawn from data analyzed at +30 mV, and therefore the model may not be generalizable across all voltages. Improved resolution of the substate gating is necessary before we can comment on the voltage-sensitive transitions between the different subconducting open states.

## Conclusions

From the relationship between voltage and subconductance Po (Fig. 3 *B*), although we cannot measure channel openings at 0 mV in symmetrical conditions, we can extrapolate the data to suggest that TRIC-B is normally gating to subconductance open states at potentials close to 0 mV with only rare openings to the full open state. In the cell, as there is thought to be no large resting membrane potential across the SR (40), TRIC-B would be expected to be gating in a low Po mode and primarily to subconductance open states unless there was buildup of negative charge in the SR relative to the cytosol. The openings of many channels, even to subconductance levels, would allow equilibration of monovalent cations across the SR membrane (15) and would also contribute charge compensating current at any time that there was Ca^2+^ flux across the SR although, obviously, the SR K^+^ flux would be more limited than if channels opened with high Po to the full open state. We have focused on the voltage-dependent channels in this study although ∼11% of channels are voltage-independent. Because voltage-independent channels exhibited high Po at the voltages monitored (12), and since these channels may physiologically constitute a proportion of the channels in the cell, this adds to the likelihood that, at least some TRIC-B channels will be open at SR membrane potentials near 0 mV. The SR membrane composition is more complex than our simple neutral bilayer and it will be important to evaluate how the interrelationship between subconducting and full open states is altered at 37°C and within a physiological environment, particularly with a phosphatidylserine component to the membrane, because negative charge has been shown to affect SR K^+^ channel function (4,41,42). Other factors including temperature, pH, and divalent cations (1,21,22) may also be important. For example, rapid and large changes in cytosolic and luminal [Ca^2+^] take place during excitation-contraction coupling in both cardiac and skeletal muscle and could potentially influence channel gating. Widely inconsistent effects of cytosolic and luminal Ca^2+^ on SR K^+^ channel gating have been reported (21,43,44). This may be due to the difficulty of distinguishing between subconductance gating and block of the channel by Ca^2+^, especially when events are poorly resolved, thus showing the need to investigate SR K^+^ channel behavior at high resolution and when only a single channel is present in the bilayer.

## Author Contributions

T.I., M.N., and H.T. produced and characterized the TRIC-A knockout mice and provided tissue. E.V. and F.O’B. isolated SR membrane vesicles. A.M., E.V., and F.O’B. performed experiments, analyzed data, and produced figures. A.M., K.T.-A., and R.S. developed models of gating. A.M. performed simulations. A.M. and R.S. wrote the article. All authors discussed the results and commented on the article.

## Supporting Citations

References (45–48) appear in the Supporting Material.

## Supporting Material

Document S1. Supporting Materials and Methods, Supporting Results, 11 figures, and four tables

Document S2. Article plus Supporting Material
