# Supplementary material for: Subconductance Gating and Voltage Sensitivity of Sarcoplasmic Reticulum K+ Channels: A Modeling Approach
Source: Biophys J. 2015 Jul 21;109(2):265–76. doi: 10.1016/j.bpj.2015.06.020 (PMC4623209; doi:10.1016/j.bpj.2015.06.020)
Supplement: Document S1. Supporting Materials and Methods, Supporting Results, 11 figures, and four tables [file mmc1.pdf]

Sub-conductance gating and voltage-sensitivity of sarcoplasmic reticulum K^+ channels: a modeling approach

Supporting Material

Antoni Matyjaszkiewicz[1,2], Elisa Venturi[3], Fiona O'Brien[3], Tsunaki Iida[4], Miyuki Nishi[4], Hiroshi Takeshima[4], Krasimira Tsaneva-Atanasova[5] and Rebecca Sitsapesan[3,*].

[1]Bristol Centre for Complexity Sciences, University of Bristol, UK
[2]Department of Engineering Mathematics, University of Bristol, UK
[3]Department of Pharmacology, University of Oxford, UK
[4]Graduate School of Pharmaceutical Sciences, Kyoto University, Japan
[5]College of Engineering, Mathematics and Physical Sciences University of Exeter

Table of Contents

Supporting Methods and Results **3**
Suitability of M1 and M2 3
Computation of simulated data and use of mean currents 4
Computing the Earth Mover's Distance (EMD) 5
Further analysis involving the EMD: multidimensional scaling 5
Fast gating model: effects of filtering on sub-state noise 7
Supporting References **8**
Supporting Figures **9**
Figure S1. Transition pathways between open and closed channel states 9
Figure S2. Steady-state channel gating behaviour 10
Figure S3. Model M1-adapted (adapted to include multiple full open and closed states) and typical examples of simulated data 11
Figure S4. Amplitude histograms for individual experimental recordings, with corresponding simulations of model M1-adapted 12
Figure S5. Amplitude histograms for individual experimental recordings, with corresponding simulations of model M1-reduced 13
Figure S6. Amplitude histograms for individual experimental recordings, with corresponding simulations of model M2-adapted 14
Figure S7. Amplitude histograms for individual experimental recordings, with corresponding simulations of the candidate fast-gating model 15
Figure S8. Mean amplitude histograms of model simulations with rates fitted globally to experimental data 16
Figure S9. Comparison of lifetime distributions arising from idealisation in QuB with M1 or M2 using the kernel density method 17
Figure S10. Application of multidimensional scaling to EMDs between models and data 18
Figure S11. Comparison of filtering effects on experimentally observed sub-states, and sub-states generated by the fast-gating model. 19
Supporting Tables **20**
Table S1. Lifetime analysis for full open and closed states of single voltage-dependent channels 20
Table S2. Observed direct transitions between the closed and full open state 20
Table S3. Comparison of experimentally observed mean single-channel currents with simulated mean currents derived from the various models 21
Table S4. Transition rates for model M1-adapted 22

Supporting Methods and Results

Suitability of M1 and M2

Model suitability was assessed using 8 representative recordings where only single channels were gating in the bilayer. TRIC-B channels exhibit variable gating kinetics (1) so we used only channels exhibiting voltage-dependent behaviour. Fitted events from idealisations derived using model M1 in QuB (2) were checked visually to assess whether sub-state events from QuB matched those that could be classified by eye. For holding potentials of +30 mV and above, where the amplitude of openings was sufficiently large to resolve distinct sub-conductance states, visual inspection revealed a close match to events fitted in QuB. However, smaller amplitude sub-conductance state openings, for example, those observed at holding potentials of 10 or 20 mV, were not easily differentiable from background noise.

The more coarse-grained idealisations fitted using model M2 were compared visually both to the data and to idealisations produced by model M1. An initial comparison of Po obtained from amplitude histograms indicated that model M2 was able to correctly identify regions where the channel was fully open or closed, and distinguish these from regions of sub-conductance gating. Without stripping events below 0.6 ms, idealisations produced by M2 had a mean overlap of 99.0% ± 0.7 (SD; n=8) with idealisations produced using M1. Overlap was defined as the per-sample correspondence, for each experimental recording, between the idealisations produced by each model. A digitised sample point of an experimental recording classified as the same state by both M1 and M2 would count as an overlap, but a sample classified as one state (open, for example) by M1 and as another state (closed, for example) by M2 would not count as an overlap between the two models. After removal of events < 0.6 ms, as in all further analysis, idealisations produced by M2 had a mean overlap of 99.3% ± 0.6 (SD; n=8) with idealisations produced using M1. Considering sublevel events only, on average 95.0% ± 3.5 (SD; n=8) of sublevel events classified by M2 overlapped with those classified by M1.

Lifetime distributions resulting from idealisations computed by both models were compared using the two-sample Kolmogorov-Smirnov (K-S) test (3). P=0.05 was chosen as a suitable level at which to reject the null hypothesis (that the two distributions, M1 and M2, come from the same underlying distribution). M1 idealisations were pre-processed to make them comparable to those produced by M2, since M1 contains multiple sub-conductance states while M2 contains only a single merged sub-conductance state. M1 sublevel events (S4 to S1) were all assigned to a single merged state, as in M2, and consecutive events of the new merged sublevel were combined into single longer events. Computed K-S distance values, D, and the corresponding critical distance for P=0.05 for each set of events are shown, for two representative experimental recordings in Fig. S9. The idealisations of all voltage-dependent channels were compared in this manner. Sparse lifetime distributions with fewer than 250 events were not included in the comparison. For all of the distributions analysed, D was lower than the critical value for P=0.05, meaning that the lifetime distributions arising from idealisations computed using M1 and M2 were not significantly different at this level. Additional visual inspection of the distributions (smoothed using the kernel density estimation method introduced by Rosales et al. (4) to reduce binning errors in regions with few samples) indicated that all distributions considered here had similar shape and number of components (Fig. S9). Model M2 is therefore comparable in classification performance to Model M1 in discriminating between currents belonging to full open, closed or sub-conducting open states but not between individual sub-conductance open states.

Computation of simulated data and use of mean currents

The mean currents obtained from voltage-dependent experimental recordings (from QuB) were used for comparing models and were calculated as described in the main Methods. For simulated data, state amplitudes and standard deviation were known *a priori* and were input to each model. The mean currents were computed directly from amplitude histograms by calculating the centroid of each amplitude distribution as follows: We can define a finite amplitude distribution x over the points x_i, representing currents, with corresponding weights w_i, such that

$$x = \{(x_1, w_1), (x_2, w_2), \dots, (x_n, w_n)\},$$

where n is the number of points in the distribution. The total weight of the distribution is

$$w_\Sigma = \sum_{j=1}^{n} w_j.$$

The mean current is then the centroid $\bar{x}$ of the distribution,

$$\bar{x} = \frac{\sum_{j=1}^{n} w_j x_j}{w_\Sigma}.$$

Model simulations were performed as described in the main text. Convergence criteria were the relative change in the centroid of each distribution, and the relative sum of squared residuals, between consecutive iterations of the simulation (30 seconds of simulated data for the fast-gating model, and 60 seconds of simulated data for all the remaining models per iteration) since both the mean current and the shape of the distribution were used to assess model correspondence to experimental data. The smallest resolvable difference in digitised current is 10^{-3} pA. This value was chosen as an appropriate relative error tolerance for changes in mean current (centroid) computed from each distribution. Likewise, a value of 10^{-6} pA^2 was chosen as the relative error tolerance for the sum of squared residual differences. Convergence occurred in under 1000 iterations, corresponding to a total of between 500 and 1000 min of simulated recordings for a given condition, depending on the model type. For the M1-adapted, M1-reduced, and M2 models, where state amplitudes and corresponding noise distributions are explicitly parameterised, it is possible to analytically compute amplitude distributions by calculating the equilibrium Po distribution for a given set of rates. Given a transition rate matrix, $\boldsymbol{Q}$, the transition probability matrix is defined as $\boldsymbol{P} = e^{\boldsymbol{Q}\Delta \boldsymbol{t}}$, with entries $p_{i,j} = \Pr(X_{n+1} = j \mid X_n = i)$, and $\Delta \boldsymbol{t}$ the sampling interval of the simulation (5, 6). For a given probability matrix, $\boldsymbol{P}$, the equilibrium Po distribution, $\boldsymbol{\pi}$, a row vector whose entries correspond to the equilibrium Po of each Markov state in the model, is, by definition, unchanged under multiplication with $\boldsymbol{P}$, so $\boldsymbol{\pi P} = \boldsymbol{\pi}$ (5, 6). This is related to the standard eigenvector problem and so we can compute $\boldsymbol{\pi}$ by calculating the eigenvalues and left eigenvectors of $\boldsymbol{P}$, so that

$$\boldsymbol{\pi} = \frac{\boldsymbol{v}}{\sum_i v_i}$$

where $\boldsymbol{v}$ is a left eigenvector of $\boldsymbol{P}$ with a corresponding eigenvalue of 1. The analytical current amplitude distribution is then simply a sum of Gaussian functions whose amplitudes correspond to the equilibrium Po, and whose means and standard deviations correspond to the current amplitude and noise for each state, as parameterised from each experimental recording. The analytical distributions were used to confirm that the simulated data were converging correctly. The simulated data, (rather than the analytically derived amplitude distributions) were used when comparing models since the

properties of *observed* 'sub-conductance levels' cannot be calculated analytically in the case of the fast-gating model

Computing the Earth Mover's Distance (EMD)

The same simulated amplitude distributions as those discussed above were used when computing Earth Movers Distance (EMD) to experimental recordings for each model. EMD was computed as described in (7).

In order to compute EMD we need to calculate the cumulative distribution function (CDF) of x defined as:

$$\mathrm{CDF}_x(t) = \begin{cases} 0 & \text{if } t \in (-\infty,\ x_1) \\ \sum_{i=1}^{k} w_i & \text{if } t \in [x_k,\ x_{k+1}), 1 \le k \le n-1. \\ w_\Sigma = \sum_{i=1}^{n} w_i & \text{if } t \in [x_n, \infty) \end{cases}$$

If we compute two CDFs, one for a model and one for a dataset, we can compute the EMD between the two distributions as

$$\mathrm{EMD(data, model)} = \frac{E}{w_\Sigma},$$

where E is the total work performed in the optimal transfer of area (mass) between the two CDFs. In our case, the problem is one-dimensional (a univariate distribution) and thus:

$$E = \sum_{k=1}^{n} (x_{k+1} - x_k)|\mathrm{CDF_{data}}(x_k) - \mathrm{CDF_{model}}(x_k)|.$$

This notation assumes that the $\mathrm{CDF_{data}}(x)$ and $\mathrm{CDF_{model}}(x)$ are defined over the same shared set of co-ordinates $X = [x_1,\ \ldots\ , x_n]$ i.e., have the same support, which they do in our data.

Further analysis involving the EMD: multidimensional scaling

We use multidimensional scaling (MDS) to compare the performance of each proposed model in relation to the data. MDS allows us to represent the gating behaviour of TRIC-B channel as points in an abstract geometric space. MDS is a well-established tool in data visualisation and data mining (8). It allows for reduction in dimensionality of the data and visualisation of relations between the objects under investigation while preserving as much information as possible. Since the EMD is a metric in the space of TRIC-B channel gating behaviour (captured by the pdfs corresponding to amplitude histograms), we use classical MDS implemented in MATLAB (The MathWorks, Inc.) as the command ‘cmdscale’ (http://uk.mathworks.com/help/stats/cmdscale.html).

We first computed the EMDs between all pdfs (experimental, as well as model simulations), which correspond to individual channels, for all pairwise combinations of data-data, model-data, and model-model amplitude histograms. Then we used the computed EMDs to construct a distance matrix **D**. Specifically we generated an $n*n$ distance matrix containing all possible pairwise interactions (where n = n(data) + n(model)*n(model fit type)). Each row of this matrix was assigned to a different amplitude histogram (and hence belongs to a specific channel) and contains EMDs between this amplitude histogram and all the other amplitude histograms. For instance, cell (2,3) contains the EMD

between second and third amplitude histograms in our dataset. Since the EMD is a metric, matrix **D** has zeros on the diagonal and is symmetric.

Next, we used the MDS to transform matrix **D** into coordinates of points in the "dissimilarities" space. In particular, following MDS, each amplitude histogram is represented as a single point in two-dimensional space. Here we used only the first two dimensions of the "dissimilarities" space, which turn out to be sufficient for the purpose of our analysis since the first two eigenvalues computed in the MDS were within one order of magnitude, while the remaining eigenvalues were significantly smaller. Points corresponding to data-derived and model-generated amplitude histograms in these first two dimensions are shown in Figure S10.

In summary, MDS allows us to extend pair-wise analysis of distances between amplitude histograms observed in experiments and simulated by the models and thus gain further insight into the performance of our modelling. Furthermore, using MDS guarantees that the Euclidean distances between elements in the "dissimilarity" space are a good approximation of the EMDs between amplitude histograms.

Projecting the amplitude histograms corresponding to all models and data in the two most significant dimensions after MDS illustrates some notable distinctions between model types and fits (Figure S10).

Firstly, key qualitative differences between models are preserved, and can be visualised in the new reduced space generated by MDS. Specifically, one can see that 'individually' fitted model rates (squares, Figure S10) i.e., those where a set of rates were fitted to a single experimental recording, are reasonably close to corresponding data points (blue circles, Figure S10). This is particularly true for the M1 full (red points) and linear (yellow) models, less so for the M2 model (purple), and barely true for the fast-gating model (green). This is consistent with the fact that the simulated data from the M2 and fast gating models exhibit clear visual differences in amplitude distributions to the data (for example, compare the amplitude distributions in Figures S4 – S7 for individually fitted rates, and Figure S8 for globally fitted rates). In the case of model M2, this is due to all sub-state amplitudes being merged into one; for the fast-gating model, this is a result of sub-state noise being invariant of the data to which the model rates were fitted. Variations in the differing abilities of the models to reproduce observed data are also highlighted when we look at the average Euclidean distances between models and data in the reduced co-ordinate space. For all models, the distance between individual fits (squares) and their corresponding data points (circles) is much smaller than the distance between global fits (stars) and their corresponding data points (circles). Full and linear models are the closest to the data, with the fast-gating model over four times further away on average.

Conversely, the points corresponding to globally fitted rates (stars in Figure S10) are much closer to the mean data distribution (black diamond). Finally, some important qualitative distinctions between model types can be made by considering the points corresponding to globally fitted rates for each model type. The globally fitted linear M1 model is almost exactly equivalent to the full M1 model, indicating that the reduced kinetic scheme performs favourably in reproducing observed behaviour, compared to the original scheme. These two models are also closest to the data mean in the space generated by MDS, crossing directly over it, which the other models do not. The M2 model appears to be flexible in reproducing a range of different observed data, however it is not as close to the experimental points on average as the M1 models. The fast gating models' points are tightly clustered. This makes sense as the model is less flexible than the others; it is unable to take into account per-data variation in sub-state noise, meaning it cannot reproduce all the variations in sub-state amplitude

distributions observed across our data. However, on average it is still not much further from the mean of the data than the M2 model.

We can conclude that although M2 is good for data classification, and simulation of average currents, it does not accurately capture the complexities of observed sub-state gating kinetics. The M1 models are only good for classifying data when there is a good signal-to-noise ratio. However they are able to reproduce observed data, after fitting of kinetic rates, more closely than the other models. In addition, the reduction of the full M1 scheme to a linear kinetic scheme doesn't appear to increase its distance from the observed data. The fast gating model does accurately reproduce some kinetic aspects of the observed data (since inter-sub-state kinetics are based on those fitted by the linear M1 model). However it cannot reproduce all of the variation in observed experimental data, particularly that relating to sub-state noise. It is the furthest of all our models from the experimental data.

Fast gating model: effects of filtering on sub-state noise

Eight single channel recordings obtained at +30 mV were analysed, and compared to simulated data obtained from the fast gating model (Figure 6C). Analysis of a representative recording is shown in Figure S11. Similar trends were observed across all 8 recordings analysed.

Experimental recordings were idealised using model M1, after filtering at 1 kHz, as discussed in the main material. Each raw experimental recording was then filtered using a Gaussian filter at multiple cut-off thresholds in the range 250–1500 Hz in 250 Hz increments. The data points corresponding to each sub-state in the previously obtained idealisation were then extracted from the filtered data (only events lasting longer than 1 ms were included to reduce the filter's effects on short events). The fast-gating model was simulated as discussed in the main material, with fast rates calibrated to generate appropriate amplitudes for each of the four sub-states, and then filtered in the same way as the experimental data. Distributions of simulated (blue) and experimentally obtained (red) data under a range of filtering thresholds are shown in Figure S11.

For the small amplitude sub-state S4, the amplitude distributions for both model and data have a low median amplitude, with the distributions exhibiting a tail into higher current amplitudes. The large amplitude sub-state exhibits the converse behaviour: a high median with a tail into smaller amplitude currents. This is consistent with what would be expected from a flickery opening/closing. The current distribution of the mid amplitude sub-state S3 exhibits little bias in either direction in simulated data, which is to be expected since transitions are occurring in an approximately equal ratio of closed:open in order to generate the intermediate current amplitude that is observed. Changes in the noise distributions for experimental data are consistent with what is seen in simulations of the fast-gating model, meaning that we cannot entirely discount the possibility of a fast gating mode between the closed and open state of the channel being the mechanism behind observed sub-conductances. Furthermore, increasing the filtering cut-off threshold clearly leads to greater spread and hence variability in current amplitudes observed in both the data and model. However, the fast-gating model is unable to completely reproduce the exact noise characteristics observed in experimental data (Figure S11).

Supporting References

1. Venturi, E., A. Matyjaszkiewicz, S.J. Pitt, K. Tsaneva-Atanasova, M. Nishi, D. Yamazaki, H. Takeshima, and R. Sitsapesan. 2013. TRIC-B channels display labile gating: evidence from the TRIC-A knockout mouse model. Pflugers Arch. 465: 1135–48.

2. Qin, F. 2004. Restoration of single-channel currents using the segmental k-means method based on hidden Markov modeling. Biophys. J. 86: 1488–501.

3. Massey, F.J. 1951. The Kolmogorov-Smirnov Test for Goodness of Fit. J. Am. Stat. Assoc. 46: 68–78.

4. Rosales, R.A., W.J. Fitzgerald, and S.B. Hladky. 2002. Kernel estimates for one- and two-dimensional ion channel dwell-time densities. Biophys. J. 82: 29–35.

5. Qin, F. 2007. Principles of single-channel kinetic analysis. Methods Mol. Biol. 403: 253–86.

6. Colquhoun, D., and A.G. Hawkes. 2009. A Q-Matrix Cookbook. In: Sakmann B, E Neher, editors. Single Channel Recording. Springer. pp. 589–633.

7. Cohen, S., and L. Guibas. 1997. The earth mover's distance: lower bounds and invariance under translation. DTIC Document.

8. Borg, I., and P.J.F. Groenen. 2005. Modern Multidimensional Scaling. Springer-Verlag New York.

Supporting Figures

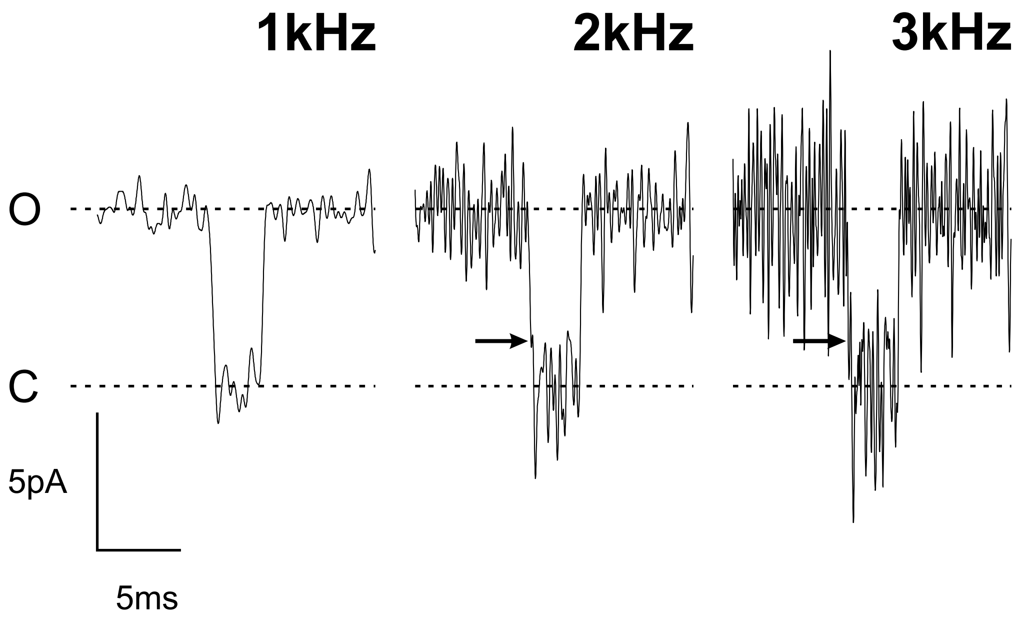

Figure S1. Transition pathways between open and closed channel states

Occasionally we observed opening or closing events that did not, when filtered at 1 kHz, appear to transition through a sub-conducting state (see Table S2). However, upon increasing filter cut-off frequency, we then found evidence for possible sub-conductance events. Representative current fluctuations are shown for an open-closed transition that initially appears not to have passed through any intermediate sub-conductance. The holding potential was +30 mV. Arrows indicate the possible sub-conductance levels that emerge as the filtering level is altered from 1 kHz (left), to 2 kHz (middle) and 3 kHz (right).

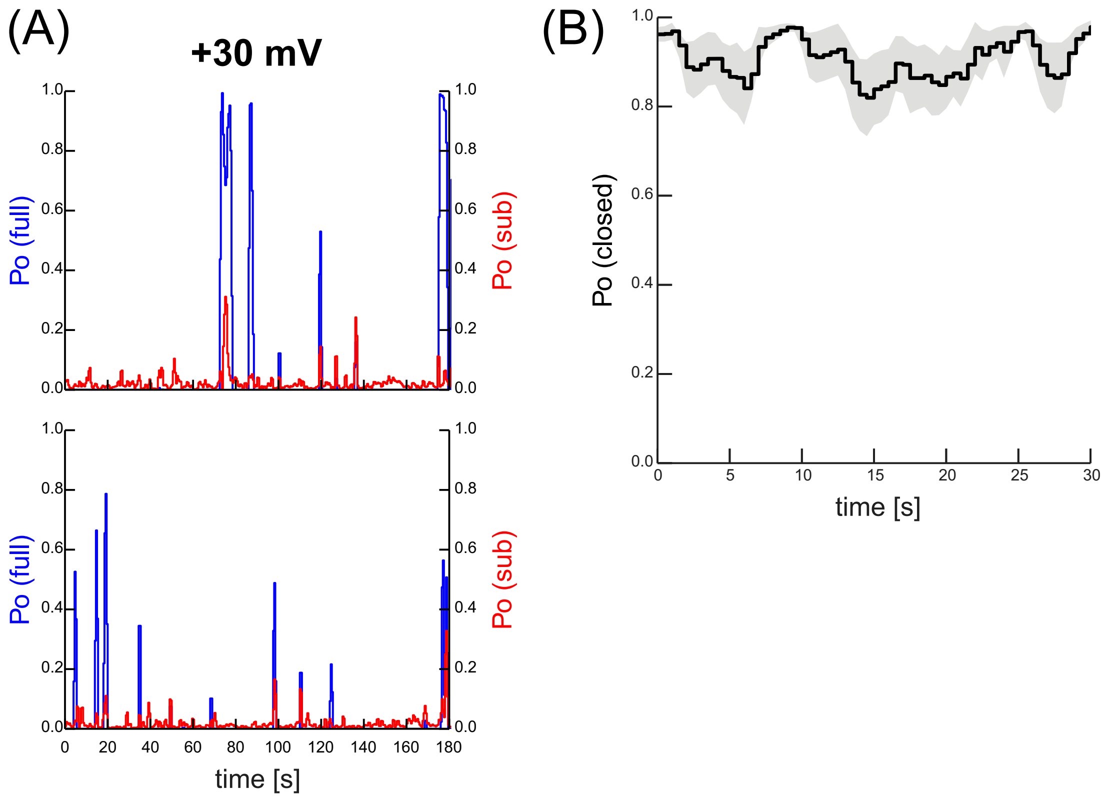

Figure S2. Steady-state channel gating behaviour

(A) Data from two typical experiments are shown in which the holding potential was held constant for 180 s at +30 mV. Single-channel records were divided into 1 s segments and the Po of each segment was calculated. The data were idealised using model M2 so that the Po for both the full open state, shown in blue, and the merged sub-conductance states (red) was obtained. (B) Mean stability plot of the closed state, for n=17 single-channel recordings at +30 mV. As in (A), the average Po for the full and sub states was computed over time using 1 s long segments. The probability of the closed state was then computed as 1 minus the Po in full and sub states. The mean pClosed across 17 recordings, of length greater than 30 s, is plotted as a black line. The shaded region indicates the region of +/- SEM from the mean. There are no significant signs of inactivation over this time period. We observed similar results for other voltages, as well as over longer periods of time (up to 100 s, although fewer recordings of this length were available).

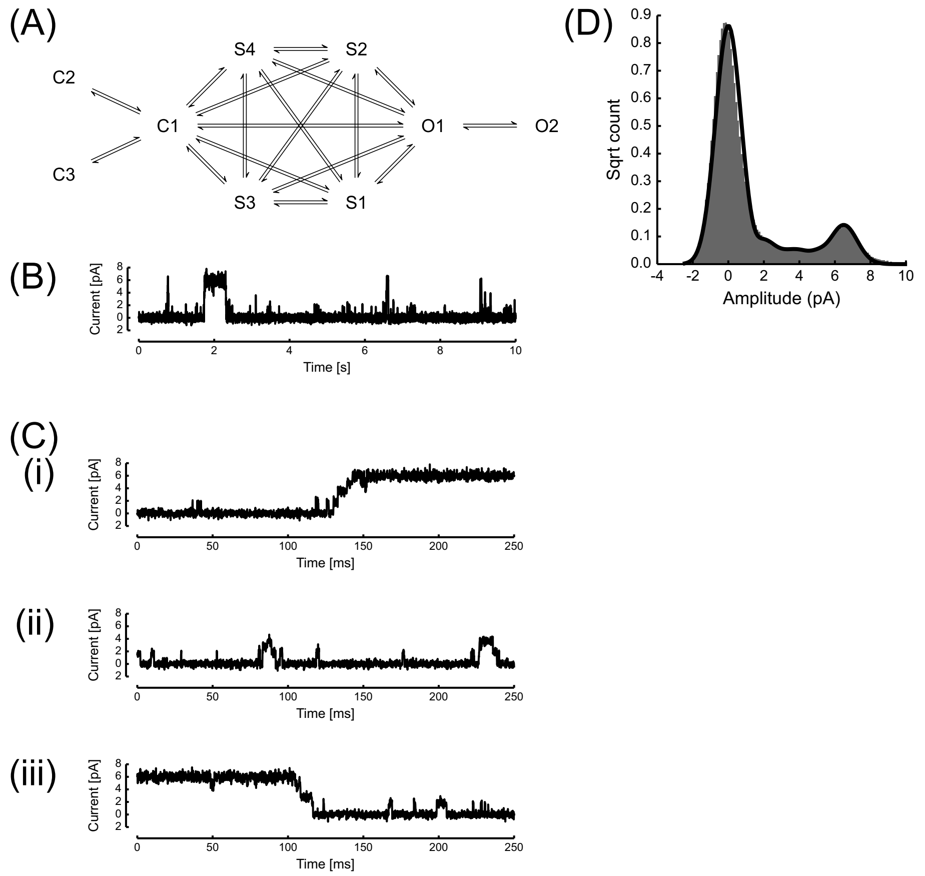

Figure S3. Model M1-adapted (adapted to include multiple full open and closed states) and typical examples of simulated data

(A) M1-adapted model topology, prior to removal of infrequent transitions. The four specified sub-conductance states in M1 were maintained in an all-to-all connected configuration with closed state C1 and fully open state O1. The closed states C2 and C3 and open state O2 were added to reflect the minimum number of time constants required to describe the open and closed lifetime distributions. Rates are omitted for clarity and are summarised in Table S4. (B) A typical example of 10 s of simulated data illustrating brief sub-conductance events that do not reach the full open level plus a typical opening burst to the full open state that lasts for several hundred milliseconds. (C) Simulated openings shown on an expanded time-scale to show the typical sub-conductance state gating that precedes the full opening burst (i), sub-conductance gating that does not transition into the fully open state (ii), and sub-conductance gating as the channel closes from the fully open state (iii). (D) Amplitude histogram of the simulated data (black line), using rate constants obtained from the same single experimental recording as in Fig. S4, S5, S6, S7 (iii), overlaid onto the current amplitude distribution of the corresponding experimental recording (grey bars). Sample counts plotted as square root.

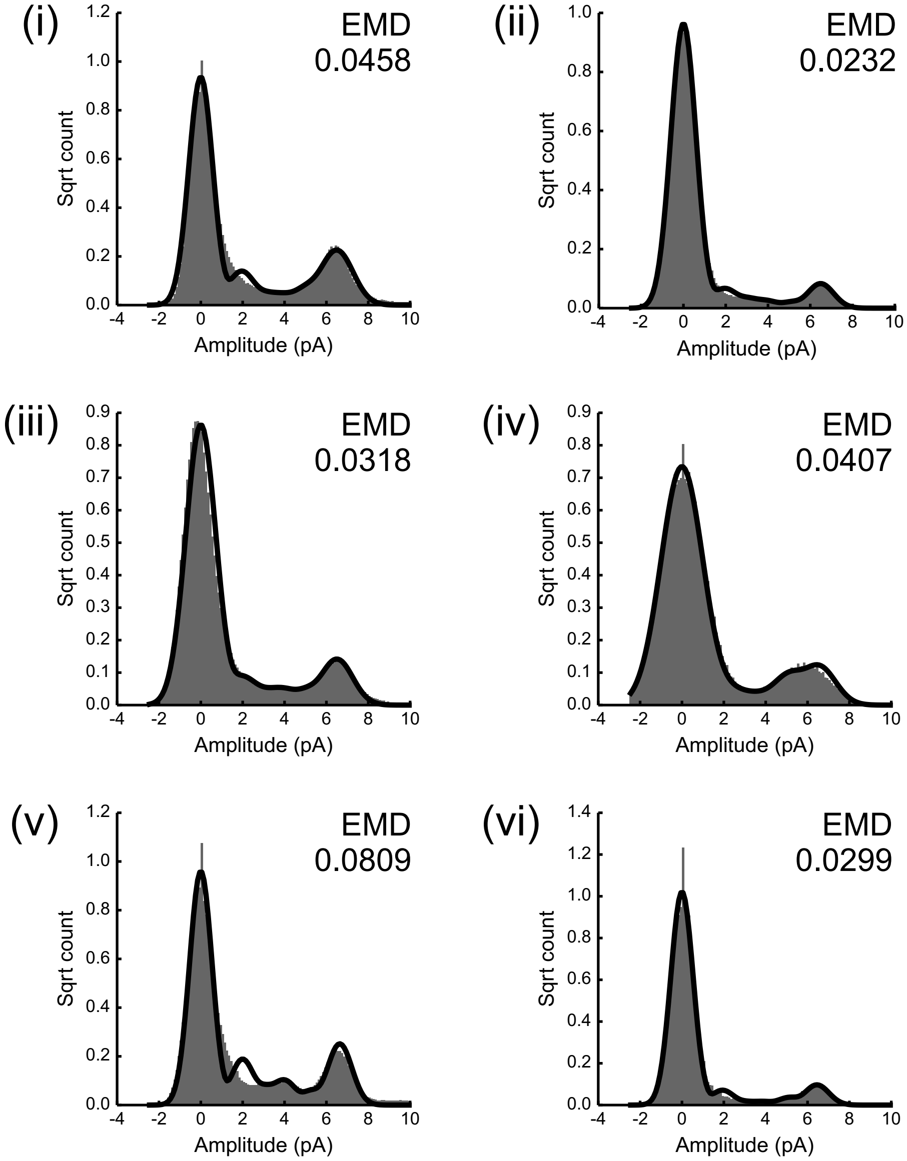

Figure S4. Amplitude histograms for individual experimental recordings, with corresponding simulations of model M1-adapted

Amplitude distributions of experimental data (grey bars) are shown for recordings of six representative voltage-dependent single channels, labelled (i) to (vi). For each experimental recording an individual set of kinetic rates was obtained for model M1-adapted, and amplitude histograms of the corresponding model simulations (black lines) are overlaid on each experimental histogram. Sample counts are plotted as square root. For each experimental recording, the EMD between the experimentally derived histogram and the corresponding model prediction is shown (Also see table S3). Note that amplitude histogram (iii) is the histogram shown in Fig. S3 (D).

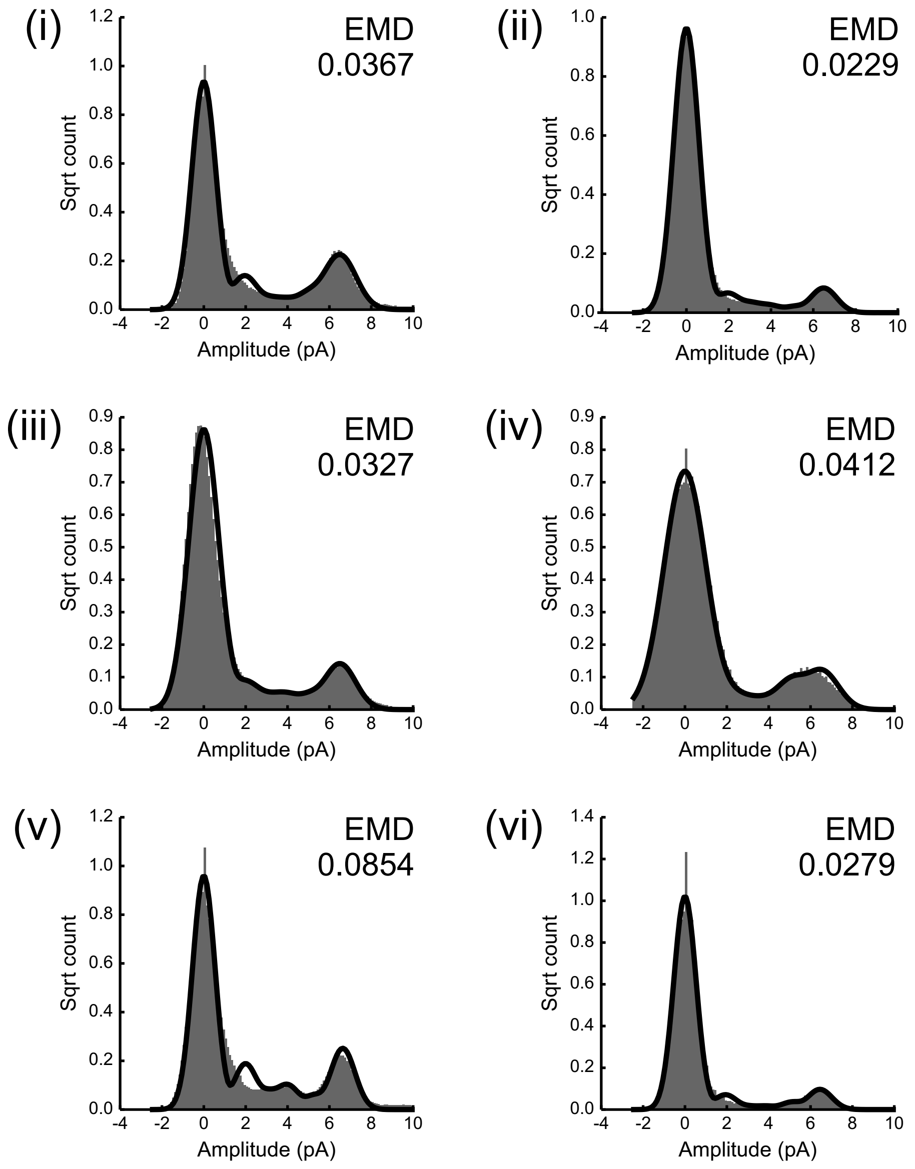

Figure S5. Amplitude histograms for individual experimental recordings, with corresponding simulations of model M1-reduced

Amplitude distributions of experimental data (grey bars) are shown for recordings of six representative voltage-dependent single channels, labelled (i) to (vi). For each experimental recording an individual set of kinetic rates was obtained for model M1-reduced, and amplitude histograms of the corresponding model simulations (black lines) are overlaid on each experimental histogram. Sample counts are plotted as square root. For each experimental recording, the EMD between the experimentally derived histogram and the corresponding model prediction is shown (also see table S3).

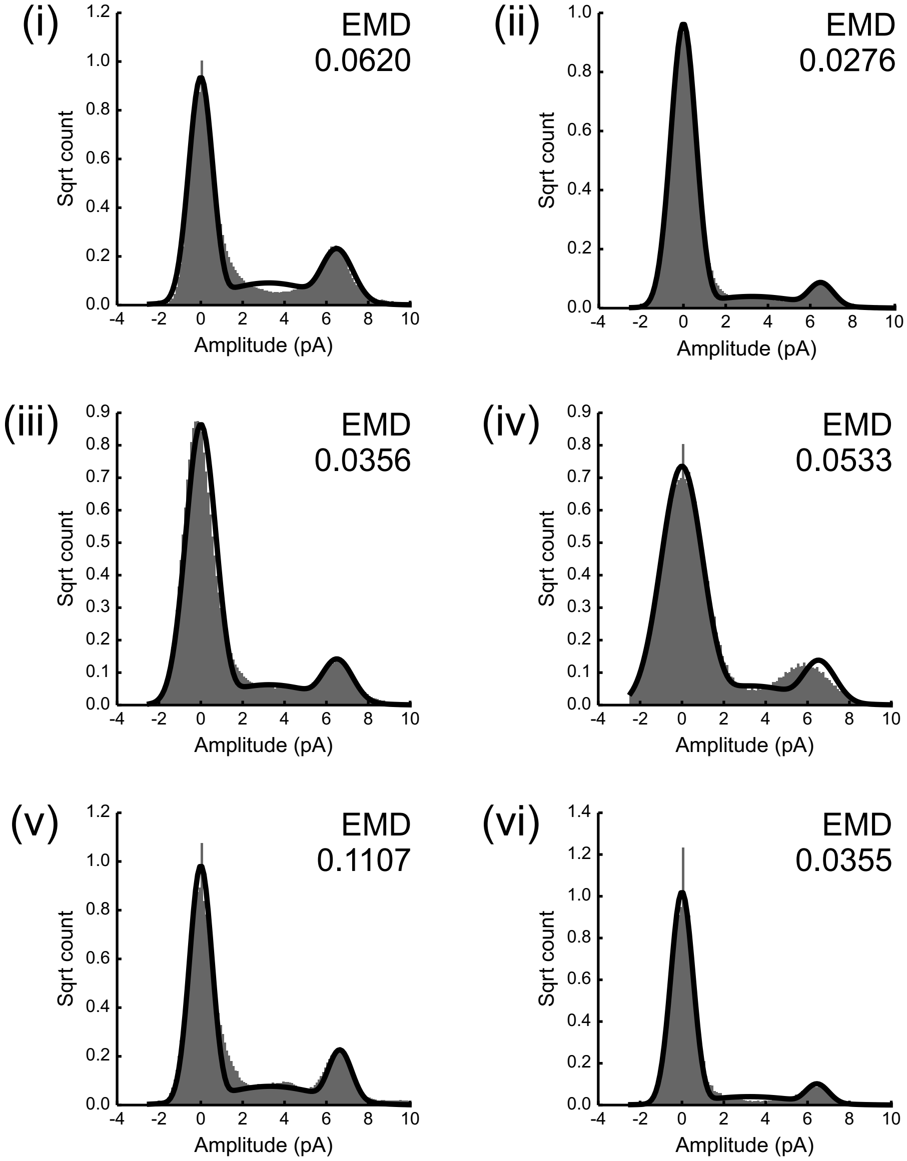

Figure S6. Amplitude histograms for individual experimental recordings, with corresponding simulations of model M2-adapted

Amplitude distributions of experimental data (grey bars) are shown for recordings of six representative voltage-dependent single channels, labelled (i) to (vi). For each experimental recording an individual set of kinetic rates was obtained for model M2-adapted, and amplitude histograms of the corresponding model simulations (black lines) are overlaid on each experimental histogram. Sample counts are plotted as square root. For each experimental recording, the EMD between the experimentally derived histogram and the corresponding model prediction is shown (also see Table S3).

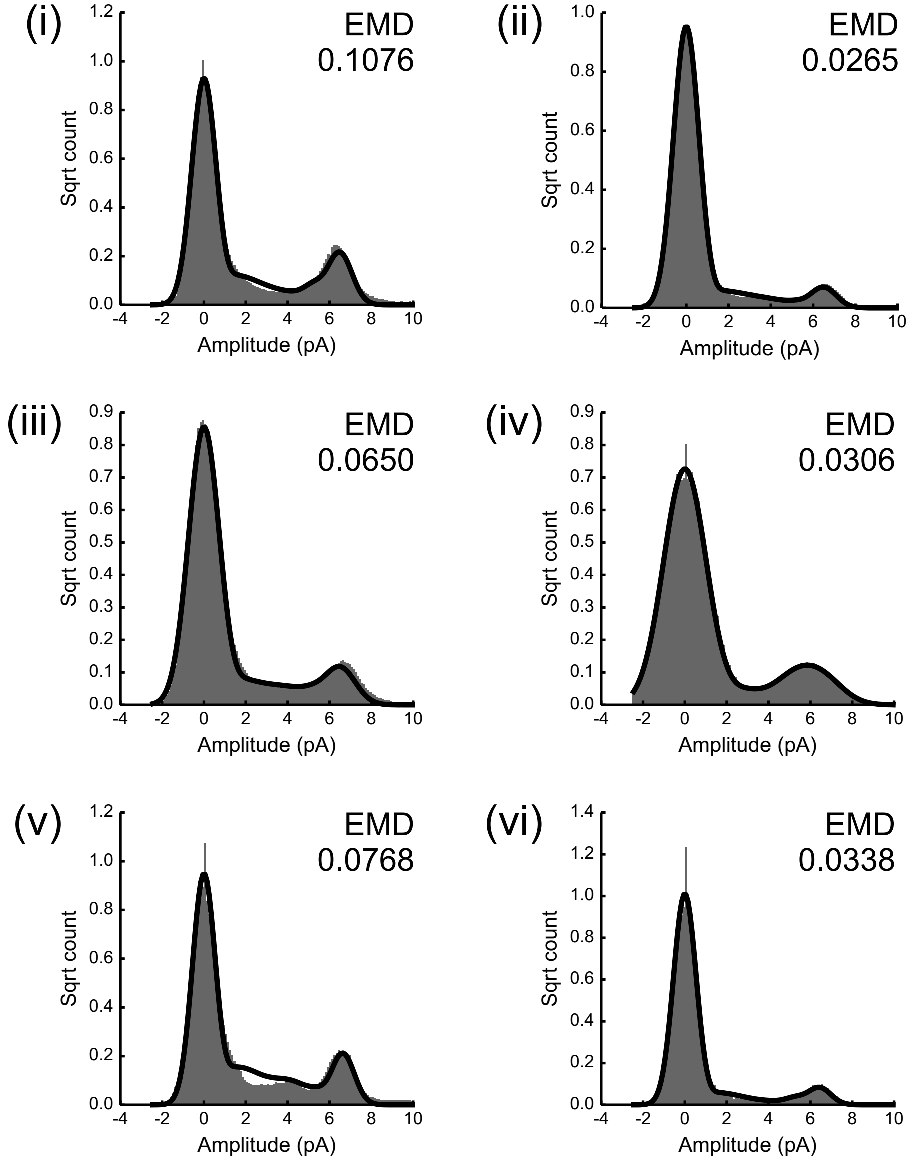

Figure S7. Amplitude histograms for individual experimental recordings, with corresponding simulations of the candidate fast-gating model

Amplitude distributions of experimental data (grey bars) are shown for recordings of six representative voltage-dependent single channels, labelled (i) to (vi). For each experimental recording an individual set of kinetic rates was obtained for the fast-gating model (corresponding to rates in M1-reduced, as discussed in the main material), and amplitude histograms of the corresponding model simulations (black lines) are overlaid on each experimental histogram. Sample counts are plotted as square root. For each experimental recording, the EMD between the experimentally derived histogram and the corresponding model prediction is shown (also see table S3).

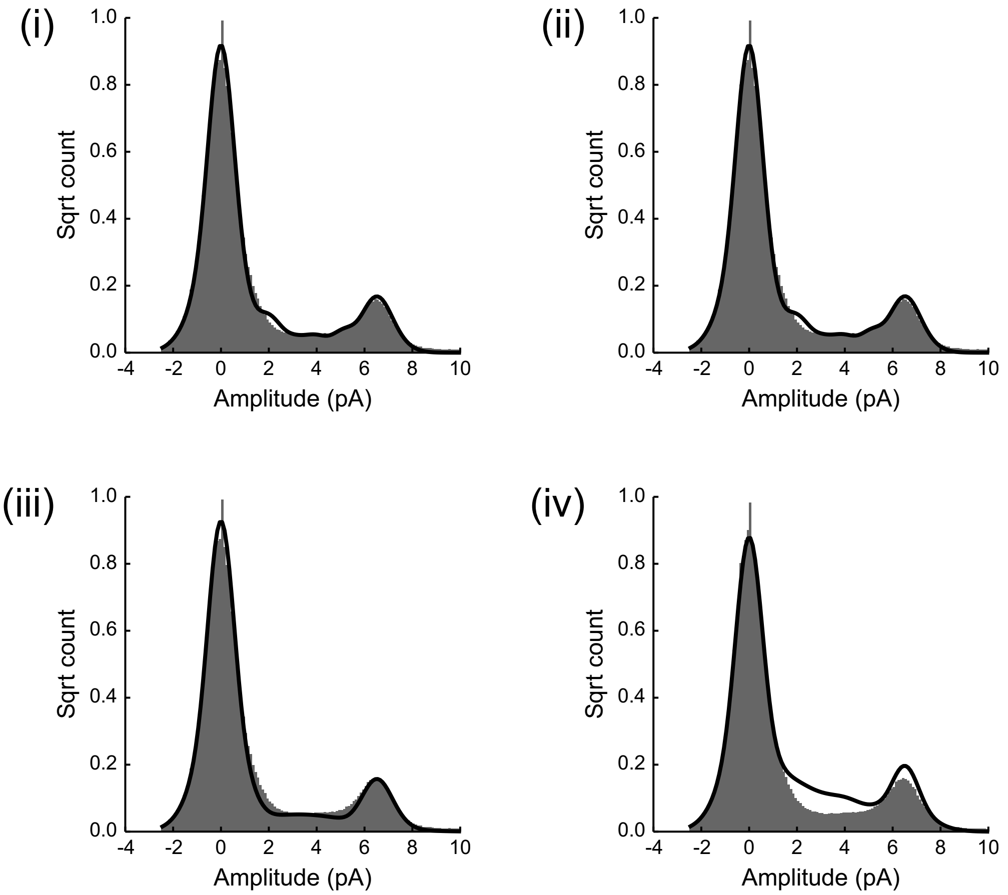

Figure S8. Mean amplitude histograms of model simulations with rates fitted globally to experimental data

The average outputs of the four kinetic models, using globally fitted rates to the six representative experimental recordings at +30 mV that were used throughout EMD analysis, are shown as simulated amplitude histograms (black lines) compared to mean amplitude histograms derived from the experimental data (grey bars). The kinetic models used were: (i) M1-adapted (Fig. S3), (ii) M1-reduced (Fig. 6A), (iii) M2-adapted (Fig. 6B)) and (iv) Fast-gating model (Fig. 6C).

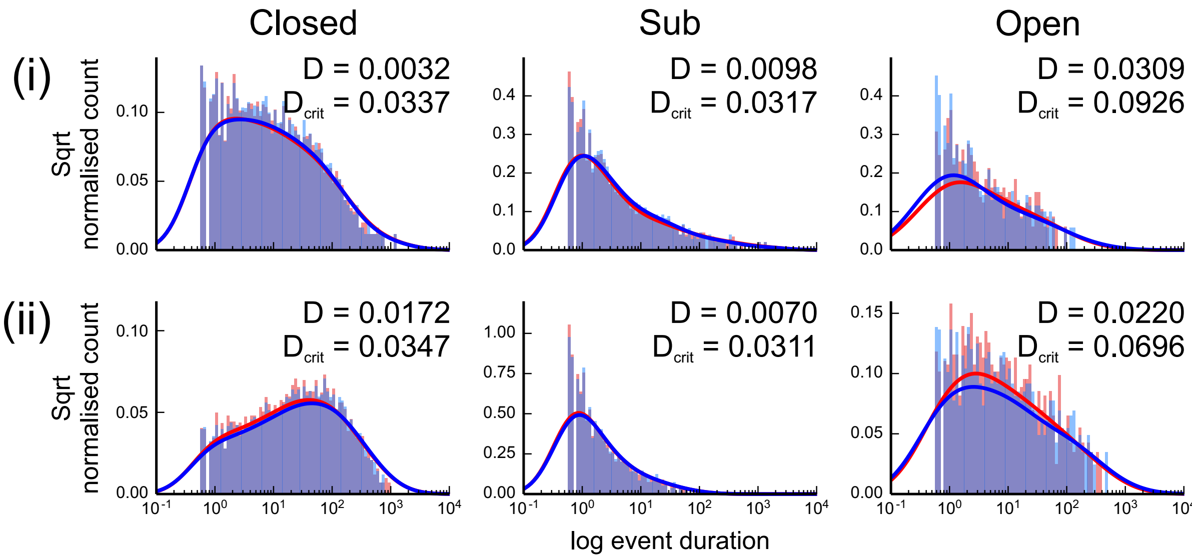

Figure S9. Comparison of lifetime distributions arising from idealisation in QuB with M1 or M2 using the kernel density method

Lifetime histograms are plotted for (left to right) the closed, sub-conductance open, and full open states from idealisations with M1 (orange bars) or M2 (blue bars) for two typical voltage-dependent channels ((i) and (ii)). Solid lines show the smoothed lifetime distribution estimated using the kernel density method (4), for M1 (red line) and M2 (blue line).

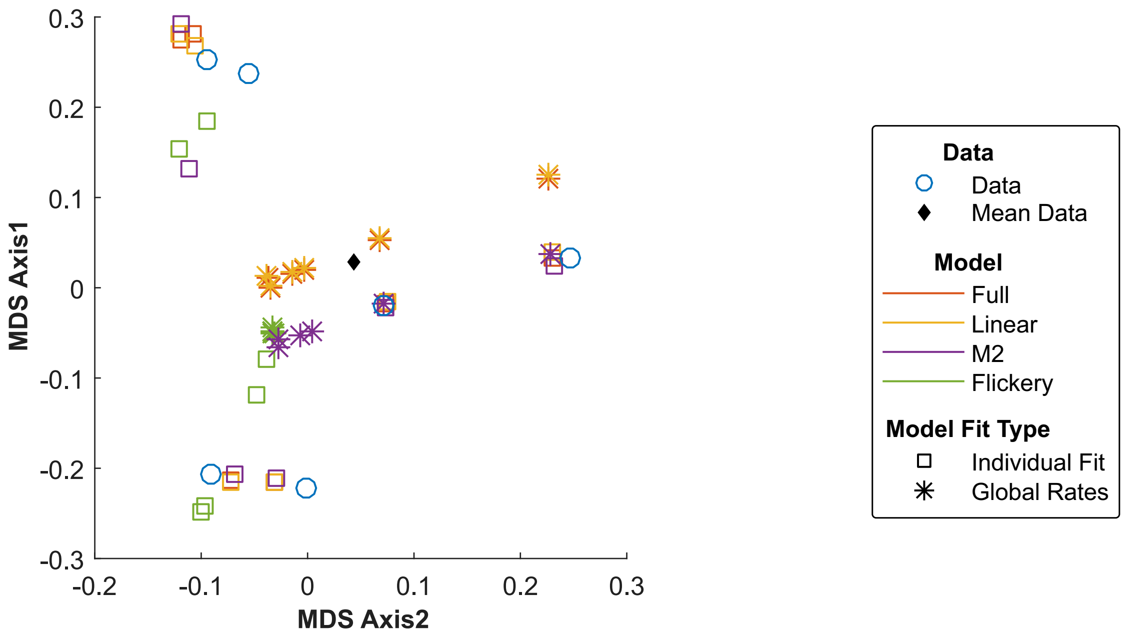

Figure S10. Application of multidimensional scaling to EMDs between models and data

The first two components of the configuration matrix after application of classic multidimensional scaling to EMDs between all model fits and data. Plot shows points corresponding to six representative experimental recordings (blue circles), the point corresponding to the mean distribution of experimental data (black diamond), and points corresponding to the amplitude distributions generated by various model fits. Symbols represent the manner in which a model's rates were fitted to data (squares: individually fitted rates; stars: globally fitted rates), and colours represent the model structure (red – full M1; yellow – reduced linear M1; purple – M2; Green – fast gating model).

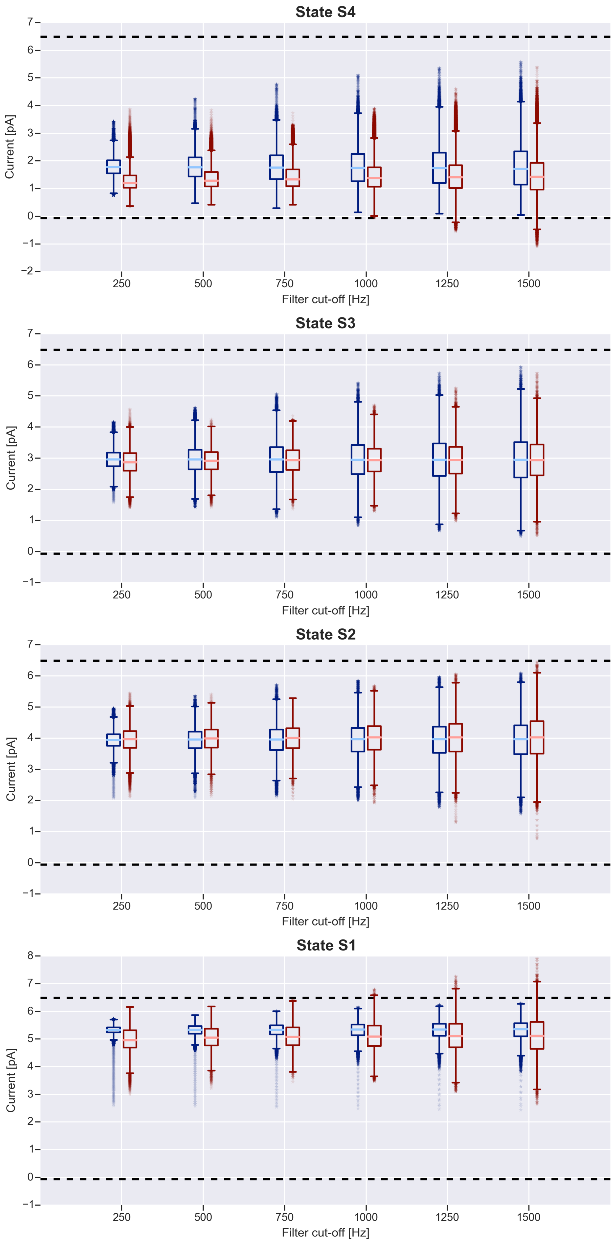

Figure S11. Comparison of filtering effects on experimentally observed sub-states, and sub-states generated by the fast-gating model.

A representative comparison of sub-state noise observed in a single experimental recording (red) to noise resulting from filtering applied to sub-states in the fast gating model (blue) is shown. The method in which this analysis was generated is discussed above. For each sub-state, data from the model are plotted in blue and experimental data are plotted in red. Boxes indicate the upper and lower quartiles of the data points at each filtering threshold. Pale lines across the centre of each box indicate the median current amplitude for a given state at a given filtering level. Whiskers extend from each box to 1.5 times the interquartile range, to indicate the approximate spread of data. Outliers are plotted as stars. Black dashed lines indicate the mean amplitude of the closed state (~0 pA), and the fully open state (~6.5 pA).

Supporting Tables

Table S1. Lifetime analysis for full open and closed states of single voltage-dependent channels

Probability density functions (pdfs) were fitted to the lifetime histograms of full open and closed states for 8 representative voltage-dependent single channels at +30 mV. The corresponding time constants and areas are tabulated for each channel. There were not enough events at -30 mV for lifetime analysis.

Closed State

Tau 1 (ms)	Area 1	Tau 2 (ms)	Area 2	Tau 3 (ms)	Area 3	Tau 4 (ms)	Area 4
1.08	13.2 %	31.95	26.5 %	231.82	60.3 %		
2.23	46.7 %	31.38	35.9 %	437.30	17.4 %		
0.80	38.5 %	10.00	42.3 %	138.99	19.3 %		
0.84	40.9 %	09.51	29.7 %	775.25	29.3 %		
0.70	12.0 %	7.87	10.4 %	81.59	40.7 %	176.13	36.8 %
0.92	13.5 %	7.54	18.5 %	38.17	43.5 %	104.58	24.5 %
0.50	21.6%	8.83	28.3%	62.42	30.5%	623.06	19.6%
1.14	29.0%	10.42	34.0%	60.55	20.5%	1122.75	16.4%

Full Open State

Tau 1 (ms)	Area 1	Tau 2 (ms)	Area 2	Tau 3 (ms)	Area 3
1.92	100.0 %				
1.97	30.3%	46.41	69.7%		
3.76	38.9%	149.88	61.1%		
0.95	31.0 %	10.85	69.0 %		
1.36	36.9 %	19.35	63.1 %		
0.92	40.8 %	16.52	59.2 %		
1.24	19.9 %	20.12	71.2 %	82.86	8.9 %
1.34	50.5 %	8.78	35.5 %	61.36	14.0 %

Table S2. Observed direct transitions between the closed and full open state

The total number of detected transitions across 8 experiments, where only a single channel is gating in the bilayer, when idealised with M1 or M2 are shown. The number of direct transitions between open and closed states (without apparent intermediate sub-conductance state transitions) is also shown. Only approximately 0.01% of all transitions were potentially direct openings or closings (passing through no intermediate sub-conductance states).

	Total number of transitions	Direct open-closed transitions	Percentage of total
**Model M1**	90839	12	0.013 %
**Model M2**	63538	7	0.011 %

Table S3. Comparison of experimentally observed mean single-channel currents with simulated mean currents derived from the various models

QuB was used to obtain the mean current (combined full open and sub-conductance state currents) for each of six representative voltage-dependent channels at +30 mV. The mean value of these currents across the six experiments was 0.289 pA. Simulated data from each of the four model types indicated in the first column was obtained. All models were adapted to incorporate the 3 closed and 2 full open states derived from lifetime analysis. Simulated recordings were obtained for each model using both individually and globally fitted rates to the six voltage-dependent channels, and the mean current was measured from the resulting amplitude distributions by computing their centroids (as described above) and is given in column 1. The second column gives the difference between the predicted model current and the mean experimental current (0.289 pA) expressed both as an absolute current magnitude (pA) and as a percentage of the experimental mean current.

To further characterise model performance, we used the Earth Movers Distance (EMD), to provide a measure of the difference (distance) between experimental and simulated amplitude distributions. This corresponds to the 'work' (total change in area) required to change the model distribution to exactly match experimental data. First, for each of the six experimental recordings, the mean EMD against all five other recordings was computed. The EMD between the six experimental recordings ranged from 0.355 to 0.417, with a mean value of 0.359. Secondly, the EMD between simulated amplitude distributions and experimental recordings was computed, and is shown in the rightmost column of this table. EMD for simulated versus experimentally derived amplitude distributions is much lower than that computed within the experimental data (0.359). This means that the amount of 'work' required to transform simulated amplitude distributions to those of corresponding experimental recordings is, on average, lower than that required to transform the amplitude distribution of one experimental recording to that of another. Therefore current amplitude distributions obtained through simulation of our models fall well within the range of variation that would be expected in our data.

	Model	Mean model current (pA)	Mean model current – mean experimental current	Mean model EMD
**Individually fitted rates**	M1-Adapted	0.2917	0.0030 (1.04 %)	0.0421
	Reduced, Linear M1	0.2909	0.0022 (0.77 %)	0.0411
	M2-Adapted	0.2692	-0.0194 (-6.73 %)	0.0541
	Fast-Gating	0.2498	-0.0388 (-13.45 %)	0.0567
**Globally fitted rates**	M1-Adapted	0.2924	0.0038 (1.31 %)	0.1778
	Reduced, Linear M1	0.2946	0.0059 (2.06 %)	0.1783
	M2-Adapted	0.2262	-0.0625 (-21.66 %)	0.1711
	Fast-Gating	0.2554	-0.0333 (-11.53 %)	0.2493

Table S4. Transition rates for model M1-adapted

The transition rates for the model shown in Fig. S3 (M1-adapted to include multiple closed and full open states) were obtained by simultaneous global fit of the data from n=8 voltage-dependent experimental recordings in QuB. The rows in the table give the rates out of a given Markov state and the columns give the rates entering a state. Zero rates (non-existent connections) are omitted for clarity. Those rates that correspond to transitions which either do not occur or have an extremely low probability of occurring across all channels, are shown in blue text. Rates are in units of sec^{-1}.

						To				
		**C1**	**C2**	**C3**	**S4**	**S3**	**S2**	**S1**	**O1**	**O2**
	**C1**		125	11.2	191	0.44	6.1 E-22	1.6 E-29	8.3 E-11	
	**C2**	12.7								
	**C3**	1.77								
	**S4**	1228				58.4	23	5.89	0.37	
**From**	**S3**	13.9			317		149	52.9	0.52	
	**S2**	8.9 E-38			151	195		163	40.7	
	**S1**	6.3			1.1 E-15	66.6	172		538	
	**O1**	5.3 E-19			1.98	9.3 E-7	9.04	112		70.1
	**O2**								31.4	
