# Supplementary material for: Subconductance Gating and Voltage Sensitivity of Sarcoplasmic Reticulum K+ Channels: A Modeling Approach
Source: Biophys J. 2015 Jul 21;109(2):265–76. doi: 10.1016/j.bpj.2015.06.020 (PMC4623209; doi:10.1016/j.bpj.2015.06.020)
Supplement: Document S2. Article plus Supporting Material [file mmc2.pdf]

Biophysical Journal Volume 109 July 2015 265–276

Article

Subconductance Gating and Voltage Sensitivity of Sarcoplasmic Reticulum K^+ Channels: A Modeling Approach

Antoni Matyjaszkiewicz,[1,2,*] Elisa Venturi,[3] Fiona O'Brien,[3] Tsunaki Iida,[4] Miyuki Nishi,[4] Hiroshi Takeshima,[4] Krasimira Tsaneva-Atanasova,[5] and Rebecca Sitsapesan[3,*]
[1]Bristol Centre for Complexity Sciences and [2]Department of Engineering Mathematics, University of Bristol, Bristol, United Kingdom; [3]Department of Pharmacology, University of Oxford, Oxford, United Kingdom; [4]Graduate School of Pharmaceutical Sciences, Kyoto University, Yoshidahonmachi, Sakyo Ward, Kyoto, Japan; and [5]College of Engineering, Mathematics and Physical Sciences, University of Exeter, Exeter, United Kingdom

ABSTRACT Sarcoplasmic reticulum (SR) K^+ channels are voltage-regulated channels that are thought to be actively gating when the membrane potential across the SR is close to zero as is expected physiologically. A characteristic of SR K^+ channels is that they gate to subconductance open states but the relevance of the subconductance events and their contribution to the overall current flowing through the channels at physiological membrane potentials is not known. We have investigated the relationship between subconductance and full conductance openings and developed kinetic models to describe the voltage sensitivity of channel gating. Because there may be two subtypes of SR K^+ channels (TRIC-A and TRIC-B) present in most tissues, to conduct our study on a homogeneous population of SR K^+ channels, we incorporated SR vesicles derived from *Tric-a* knockout mice into artificial membranes to examine the remaining SR K^+ channel (TRIC-B) function. The channels displayed very low open probability (Po) at negative potentials ($\leq$0 mV) and opened predominantly to subconductance open states. Positive holding potentials primarily increased the frequency of subconductance state openings and thereby increased the number of subsequent transitions into the full open state, although a slowing of transitions back to the sublevels was also important. We investigated whether the subconductance gating could arise as an artifact of incomplete resolution of rapid transitions between full open and closed states; however, we were not able to produce a model that could fit the data as well as one that included multiple distinct current amplitudes. Our results suggest that the apparent subconductance openings will provide most of the K^+ flux when the SR membrane potential is close to zero. The relative contribution played by openings to the full open state would increase if negative charge developed within the SR thus increasing the capacity of the channel to compensate for ionic imbalances.

INTRODUCTION

SR K^+ channels are selective for monovalent cations and their conduction properties are comprehensively described (1–9). The gating behavior of SR K^+ channels is less well understood and this is partly due to the complex subconductance state gating of these channels.

There is evidence that there may be two subtypes of SR K^+ channels termed the trimeric intracellular cation channels (TRIC-A and TRIC-B) (10–12). Mice devoid of both TRIC-A and B are not viable and die in cardiac arrest at embryonic day 10.5 (10) highlighting their necessity in the heart. Cardiac myocytes from TRIC double knockout (DKO) mice exhibit regions of swollen sarcoplasmic reticulum (SR) containing Ca^{2+}-deposits that are not present in control mice. At embryonic day 8.5 in DKO cardiac myocytes, spontaneous Ca^{2+} transients were reduced, whereas caffeine-induced release from intracellular stores was enhanced in comparison to controls (10). These observations suggest impaired SR Ca^{2+}-release and elevated SR Ca^{2+} levels in the TRIC-DKO cardiac myocytes. The importance of TRIC in intracellular Ca^{2+}-movements in other cell types including skeletal muscle (10,13), vascular smooth muscle (14,15), and alveolar epithelial cells (16) is also becoming evident. TRIC-A is expressed at particularly high levels in excitable cells, whereas TRIC-B is expressed at lower levels in all cell types. Although TRIC-B is less abundant, it is essential in some tissues. The *Tric-b* KO mouse dies at birth exhibiting severe ultrastructural abnormalities of the alveolar type II epithelial cells (16). Mutation of the *TRIC-B* gene is also associated with the hereditary bone disease, osteogenesis imperfecta (17–19).

Identifying the physiological roles of the TRIC-B isoform in these tissues requires an understanding of the single-channel properties of TRIC-B and the mechanisms governing channel activation. Because both isoforms are present in most tissues it is difficult to study the single-channel properties of each TRIC channel isoform in isolation. We have therefore made use of the *Tric-a* KO mouse, which can survive to adulthood and thus provides the means for studying

Submitted December 23, 2014, and accepted for publication June 11, 2015.
*Correspondence: rebecca.sitsapesan@pharm.ox.ac.uk or antoni.matyjaszkiewicz@bristol.ac.uk
This is an open access article under the CC BY license (http://creativecommons.org/licenses/by/4.0/).
Editor: Godfrey Smith.
© 2015 The Authors
0006-3495/15/07/0265/12 $2.00
CrossMark
http://dx.doi.org/10.1016/j.bpj.2015.06.020

the gating of native TRIC-B channels from SR membranes devoid of TRIC-A channels (10). Skeletal SR vesicles isolated from *Tric-a* KO mice were therefore incorporated into artificial bilayers under voltage-clamp conditions to study the interrelationships between channel activation, voltage regulation, and subconductance state gating.

The ability of different kinetic models to reveal the mechanisms underlying the voltage sensitivity and subconductance gating of TRIC-B channels was examined. We find that a simplified, linear, six-state gating model best encompasses the complexity of the subconductance gating behavior of the channel. Our results suggest that subconductance gating in SR K^+ channels may be more important than previously assumed because ~75% of the K^+ flux through the channels at physiological membrane potentials (close to 0 mV) may be due to substate gating.

MATERIALS AND METHODS

Isolation of SR vesicles and bilayer techniques

Light SR (LSR) membrane vesicles were isolated from *Tric-a*-knockout mouse skeletal muscle as previously described (12,20). After incorporation of light SR vesicles into planar phosphatidylethanolamine lipid bilayers, TRIC-B current fluctuations were recorded under voltage-clamp conditions in solutions of 210 mM KPIPES, 10 μM free Ca^{2+}, pH 7.2 (12). We used neutral phosphatidylethanolamine membranes to minimize the likelihood of developing changes in surface potential that would affect gating and conductance. By omitting charged lipids such as phosphatidylserine we were able to reduce fusion events and improve the chance of incorporating single K^+ channels into the bilayer; a prerequisite for analysis of kinetic gating of this channel. The *trans* chamber was held at ground and the *cis* chamber was clamped at various potentials relative to ground. Evidence suggests that SR vesicles incorporate into bilayers in a consistent orientation (21,22) such that the *cis* chamber corresponds to the cytosolic face of the SR channels and the *trans* chamber to the luminal side. The consistent voltage-dependence of TRIC-B also confirms this. To check that only single channels were gating in the bilayer, high positive potentials were applied for short periods of time to maximally activate the channel. Experiments were performed at room temperature (22 ± 2°C).

Data acquisition, analysis, and simulation

Single-channel recordings were digitized at 100 kHz and recorded on a computer hard drive using WinEDR 3.05 software (John Dempster, Strathclyde University, Glasgow, UK). Before idealization, traces were digitally filtered (Gaussian Filter with cut-off frequency of 1 kHz) and resampled at 10 kHz. Single-channel current fluctuations were idealized using the segmental k-means algorithm (23) in the QuB software suite (State University of New York, Buffalo, NY). Idealization was initially obtained using two Markov models, M1 and M2 (Fig. 1 *A*). For M1, a six-state Markov model was constructed with identical fully connected topology (see Fig. 1 *Ai*), where states consisted of the closed, open, and four subconductance states using our initial estimates of the current amplitudes of the conductance states (S4 = 60 pS, S3 = 93 pS, S2 = 121 pS, S1 = 161 pS, Full = 199 pS) as defined previously (12). For M2, the four subconductance states, S1–S4, from M1 were merged together into a single conductance class of amplitude midway between the fully closed and fully open states, with noise level (standard deviation in QuB) equal to half this amplitude (see Fig. 1 *Aii*). Open probability (Po) was measured from recordings of duration 1–3 min using model M2 except where use of model M1 is specified. Po in each state (including subconductance states) was defined as the proportion of the total duration of idealized experimental recording spent in that state.

Mean currents were computed using QuB: each trace was idealized using model M2, and mean current amplitude in each state was computed directly from underlying experimental recordings. Computational details about simulations of mean currents from models are provided in the Supporting Material.

Boltzmann curve fits were computed in GraphPad Prism 4.02 (GraphPad Software) through nonlinear least squares optimization. Transition frequencies were visualized as chord diagrams, plotted using the Circos software package (24). Lifetime distributions were computed from QuB idealizations where only a single channel was gating in the bilayer. Unless specified otherwise, events shorter than 0.6 ms were stripped from the idealized event sequences in QuB, or automatically merged where appropriate (25,26). Individual time constants were fitted with an exponential log probability density function (pdf) in Clampfit, using maximum-likelihood fitting (27) to determine the expected number of time constants for each conductance state. The optimal number of time constants for each distribution was determined using a log-likelihood ratio test (28) at a confidence level of P = 0.95. Missed events during maximum interval likelihood (MIL) rate optimization were automatically accounted for in QuB by computation of a corrected Q matrix in the MIL algorithm used to obtain kinetic rates.

Individual sets of kinetic rates for different models were obtained by fitting idealized recordings from experiments where only a single voltage-dependent channel was gating in the bilayer using the MIL algorithm implemented in QuB (25,26). We additionally used the global fitting capabilities of QuB (25,26) whereby rates are simultaneously fit across a set of idealized experimental recordings to obtain the optimal rates, henceforth referred to as global rates. Monte Carlo simulations of the models were performed with our own codes written in Python and C, using the Direct Method of the stochastic simulation algorithm (Gillespie's Algorithm (29)). For each model, traces were simulated until the stationary amplitude distribution converged (see Supporting Material for further details).

An example of a fast-gating model was also considered in which the transitions between the full open and full closed channel levels are so rapid that subconductance state events arise as an artifact of filtering the data. For the fast-gating model, simulated recordings were filtered at 10 kHz and resampled at 100 kHz to approximate the data acquisition and digitization process. At this stage, simulated Gaussian noise of equal magnitude to that observed in experimental recordings was added to the resampled simulated recordings, which were then filtered again at 1 kHz and resampled at 10 kHz to replicate the properties of physically acquired traces at the analysis stage.

RESULTS

TRIC-B channel gating behavior

TRIC-B channel gating is characterized by a number of prominent features (Fig. 1, *B* and *C*). These include: 1) voltage-dependent opening, 2) trains of subconductance events before and after a full opening burst, and 3) gating to subconductance open states without visiting the fully open state. Heavy filtering and compression of recordings as in (Fig. 1 *B*), top trace, can suggest that there is a single subconductance level at ~53% of the full open state. However, time-based expansion of traces shows that the channel can dwell in what appear to be multiple distinct sublevels (examples shown in Fig. 1 *Ci*, *ii*, and *v*). The channel also appears to gate in prolonged sublevels without fully opening (Fig. 1 *Ciii*). We have previously shown that TRIC-B

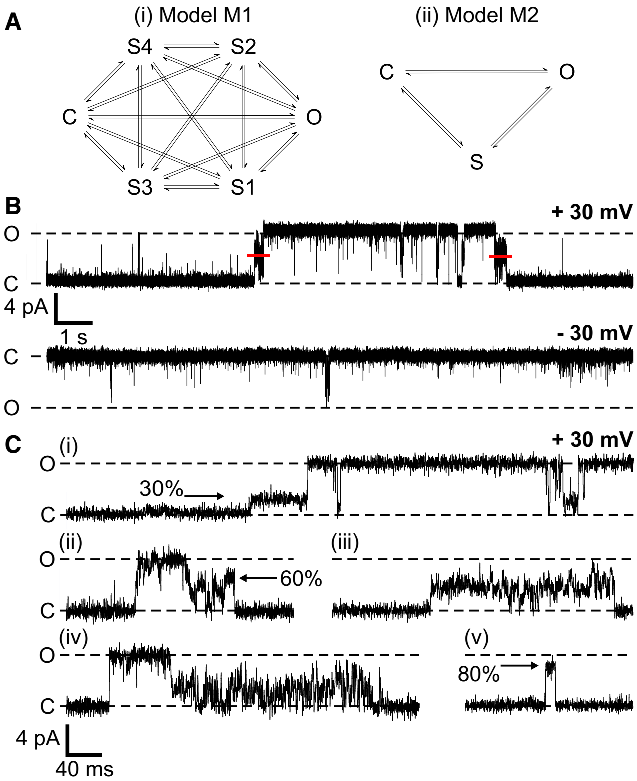

FIGURE 1 Typical single-channel recordings and gating models. (*A*) Model topologies used in QuB to idealize single-channel recordings. Model M1 (*i*) comprises six states (*full open*, *closed*, and *four subconductance open states* S1–S4 (12)), with all-to-all connectivity between states. Model M2 (*ii*) is a simplified subconductance model consisting of the full open and closed states and a single, merged subconductance state encompassing a noisy combination of all substates S1–S4. (*B*) Representative single-channel recordings illustrating typical opening bursts at +30 and −30 mV. (*C*) Expanded traces show typical subconductance state open events. The red lines show the average current level of the merged subconductance state. To see this figure in color, go online.

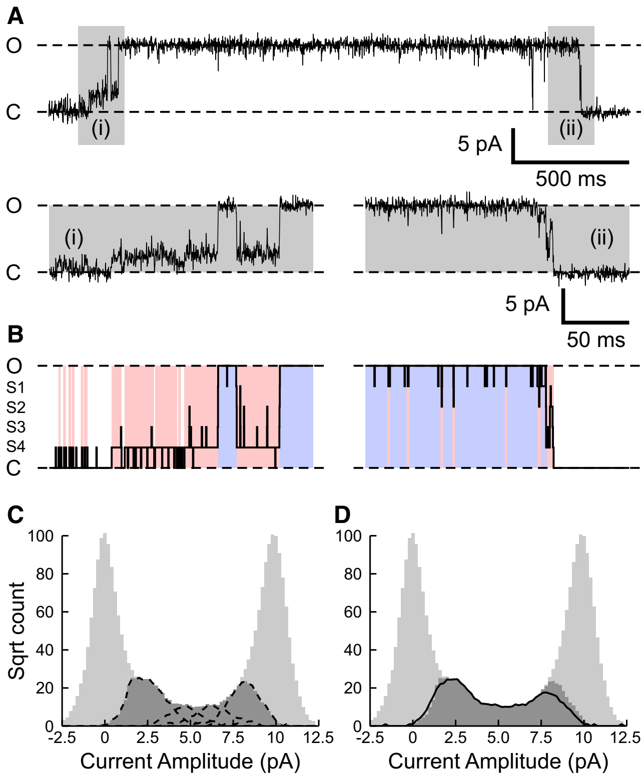

FIGURE 2 Comparing idealization of single-channel recordings using models M1 and M2. The trace in (*A*) shows a representative opening channel burst at +50 mV. Gray areas inset underneath show typical opening (*i*), and closing (*ii*), subconductance gating on an expanded timescale. Dashed lines, labeled C and O, indicate closed and full open levels, respectively. (*B*) Comparison of idealization of the opening (*left*) and closing (*right*) sequences shown directly above (*Ai* and *ii*), computed using models M1 and M2. Amplitudes labeled S1–S4 indicate approximate amplitudes of the four measured subconductance states. Solid black lines in (*B*) show idealization obtained using model M1. Idealization of the same data using model M2 is represented by colored block regions, where pink regions are classified as subconductance state and blue regions as fully open states. The amplitude histograms compare data points from the entire recording from which burst (*A*) was taken (180 s), idealized using either model M1 (*C*) or M2 (*D*). Light gray bars show the all-points amplitude distribution for the entire recording. Dark gray bars correspond to the amplitude distribution of all points in the recording classified as any one of subconductance states S1–S4 when idealized using model M1. The dashed lines in (*C*) show the amplitude distributions of data points classified as subconductance states in model M1. The solid line in (*D*) shows the amplitude distribution of subconductance states classified by model M2. To see this figure in color, go online.

appears to gate in four main subconductance open states (12), however, the variable trains of subconductance state events are difficult to quantify because the individual events can be very brief (see Fig. 1 *Ciii* and *iv*). We therefore evaluated two different levels of model complexity as a means to idealize the data (Fig. 1 *A*) and assess the contribution of subconductance state gating to the overall Po and current fluxes through the channel. Model M1 (Fig. 1 *Ai*) comprises six distinct conductance levels, corresponding to the previously reported (12), closed, full open, and four distinct subconductances (S1, S2, S3, and S4). Model M2 (Fig. 1 *Aii*) combines all subconductance openings into a single merged class. Model suitability was assessed using eight representative single-channel experiments. Further details are given in the Supporting Material.

Fig. 2 depicts a comparison of the idealization and event classification obtained by models M1 and M2 for a single typical opening burst (Fig. 2 *A*) to the full open state. The inset traces underneath show expanded views of the trains of subconductance transitions that occur before and after the sojourn in the full open state. Idealization of the expanded sections of recording shown in Fig. 2A*i* and *ii*, using model M1, are illustrated in (Fig. 2 *B*) as solid black lines. Model M1 states, and transitions in the idealization, obey a close correspondence with those observed in the recording and to idealization by model M2 (*colored background regions*). Events classified as S (*pink regions*) by model M2 may belong to any of the subconductance states classified as S1–S4 by model M1 (or to additional

subconductance states). Events classified as fully open are represented by blue regions. Crucially, the idealization produced by model M2 captures the majority of subconductance state gating behavior, distinguishing it from dwell times in the closed or fully open states. Durations of medium to long subconductance events were conserved between models M1 and M2 but the example (Fig. 2) shows that a small number of brief S1 and S4 transitions that are captured by M1, are not classified as substates by M2. Such discrepancies were rare when the recordings from all the channels were analyzed and their distributions compared (see Supporting Material for further analysis).

Conservation of subconductance state classified events between models M1 and M2 is further demonstrated in the all-points amplitude histograms for the recording shown in Fig. 2 *A* (Fig. 2, *C* and *D*). The dark gray areas highlight all points classified as subconductance states S1–S4 by model M1. Dashed lines in Fig. 2 *C* indicate the underlying amplitude distributions of currents classified as the distinct subconductance states S1–S4 by M1. In Fig. 2 *D*, the solid line illustrates the subconductance state amplitude distribution resulting from idealization using model M2. This closely matches the profile of the amplitudes identified by M1, however, this is at the cost of missing some brief subconductance events with durations close to the minimum resolvable duration.

Because model M2 correctly assigns events as open, closed, or subconductance, we used this simplified model to idealize recordings to examine the stability of gating over time. Channel Po fluctuated markedly because the randomly occurring full opening bursts were widely dispersed at all voltages as can be seen in the example Po Diary Plots shown in Fig. S2. Averaging the plots for 17 experiments (Fig. S2) provided no evidence for inactivation with time. Channels were predominantly closed at −30 mV and any openings were usually to subconductance states with rare full open events.

We investigated Po variation over a range of voltages for experiments where only a single channel was gating in the bilayer (Fig. 3). Approximately 11% of channels gate in a voltage-independent manner (12) and these channels were not included in the Po-voltage plots. Model M2 was used to idealize recordings to compare the voltage-dependence of opening to the full (Fig. 3 *A*) and the subconductance states (Fig. 3 *B*). In Fig. 3 *C*, a comparison of the mean data for full openings and subconductance states is shown. The figure illustrates large variability in Po across the channels but shows that voltage-dependent TRIC-B channels exhibit very low Po at negative potentials and predominantly subconductance events. There is a steep relationship between Po and voltage between +10 and +20 mV and thereafter Po plateaus. The critical voltage activation range for subconductance gating appears similar to that for full conductance openings and may even occur at slightly lower voltages (0 mV to +10 mV) but this is difficult to determine because of low Po, variable channel behavior, and the difficulties of resolving small amplitude subconductance events at voltages <10 mV.

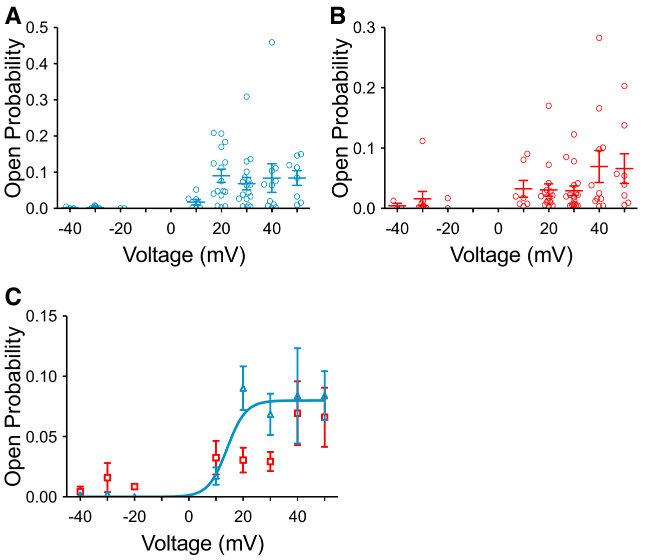

FIGURE 3 Po-voltage relationships. Po for full (*A*) and subconductance (*B*) openings as a function of voltage is shown for individual experiments. Model M2 was used to idealize the data. In (*C*), the mean data is shown for subconductance Po (*squares*) and full open state Po (*triangles*). The line corresponds to a Boltzmann fit to the Po in the full open state according to the equation:

$$Po(V) = Po_{max}\{1 + \exp([\Delta G + zFV]/RT)\}^{-1},$$

where ΔG is the internal free energy of opening, z is the effective gating charge, Po_{max} is the maximum Po, and F, R, and T have their usual meanings (fit parameters: $Po_{max} = 0.08 \pm 0.01$, $\Delta G = 9.9 \pm 1.3$ kJ mol^{-1}, $z = -7.3 \pm 0.9$). The negative z value indicates that negative charge moves in the trans (luminal) to cis (cytosolic) direction or that positive gating charge moves in the opposite direction as voltage increments from 0 to +20 mV. Mean values ± SE are shown. The following number of recordings were used at each voltage: −40 mV (3), −30 mV (8), −20 mV (2), 10 mV (7), 20 mV (12), 30 mV (17), 40 mV (10), and 50 mV (8). To see this figure in color, go online.

Lifetime analysis

Model M1 was used to assess the lifetime durations of the open, closed, and subconductance states of voltage-dependent single channels at +30 mV. At negative potentials, there were too few events for analysis. Lifetime distributions and pdfs from a typical channel for the full open and closed states are shown in Fig. 4. In this example, the best fit to the data was obtained with three closed and two open states, however, four closed states and three open states were observed in some experiments. The time constants and percentage areas for the lifetime distributions from eight representative voltage-dependent single-channel experiments are tabulated in Table S1. The mean dwell times

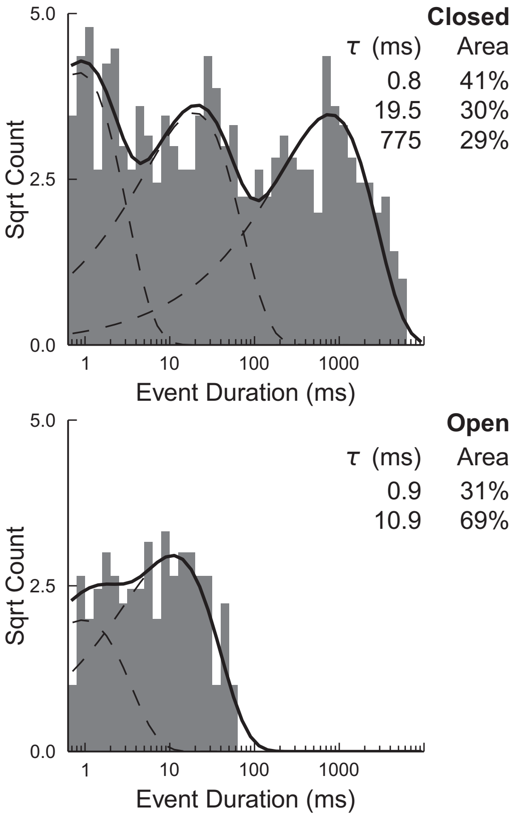

FIGURE 4 Representative open and closed lifetime distributions. Representative lifetime distributions are shown for events from a single-channel recording, idealized using model M1. Histograms of lifetime distributions (*gray bars*) are shown for the closed (*upper*) and full open state (*lower*). Durations are plotted on a logarithmic scale (10 bins per decade) and the square root of the event count is shown. Fitted time constants and their respective areas are shown for each state. The dashed lines show pdfs of individual time constants, and the solid lines show the total fitted distribution for each state. Table S1 details the data from eight experiments where only a single voltage-dependent channel was gating.

for subconductance open states, S1, S2, S3, and S4 were 1.7 ± 0.1, 2.3 ± 0.2, 2.1 ± 0.2, and 1.9 ± 0.4 ms, respectively (SE; $n = 8$). Attempts to apply lifetime analysis to the subconductance dwell times indicated that multiple tau components may be required to describe the lifetime distributions. However, because all components were very close to or below the minimum resolvable event duration, this level of detail was inappropriate for our current limits of resolution.

The multiple components required to describe the full open and closed states plus the subconductance events indicates that a high level of model complexity is required to capture channel kinetics. Certain gating motifs, however, were observed with every channel. Transitions to and from the full open and closed states almost always appear to pass through a subconductance state or a train of subconductance events (see Figs. 1 and 2). Similar findings have been reported previously for native frog skeletal SR K^+ channels (2). We found only a handful of events (see Table S2) that appeared not to transition through subconductance states before entering full open or closed states. With these rare events, if the filter cut-off frequency was increased, the suggestion of a sublevel event could be observed indicating that we may simply be missing subconductance events due to inadequate resolution. Fig. S1 shows an example of such an event.

The transition frequencies between all states were examined to determine if there were any dominant transition routes. Chord diagrams that provide a visual means of identifying the preferred transition routes into and out of the various states at ±30 are shown in Fig. 5. The diagrams depict the mean data from eight channels and emphasize that there are at least 10 times fewer transitions from each state at −30 mV than at +30 mV. At both −30 mV and +30 mV the most frequent transitions are between the closed and S4 states. Note also that, at −30 mV, a large proportion of transitions from S3 also drop back to the closed state, whereas, at +30 mV, transitions from S3 to S2 are more frequent. At +30 mV, there is also a shift to a greater number of transitions between the full open and S1 states.

Kinetic gating models

A pattern emerged that was true for both positive and negative voltages: transitions out of any state appear to lead to states that are closest in current amplitude to that state. For example, from S2, the most likely transition route is to S1 or S3. From S3, the channel is three times more likely to enter S4 or S2 rather than any other state. At −30 mV, there were too few events to examine the transitions any further. In four of the eight experiments used, there were no openings at −30 mV. At +30 mV, however, we can modify model M1 (Fig. 1) to incorporate the fact that we observe at least three closed states and at least two full open states (Fig. S3), and then take into account the likelihood of the various connections between states (see Fig. 6 *A*). In this reduced, linear gating M1 model (henceforth termed M1-reduced), the solid lines show only those transitions that were consistently observed in all eight channels and therefore highlight the preferred transition routes. Examples of single-channel traces simulated by the M1-reduced model are shown in Fig. 6 *A* to illustrate typical features and show agreement with experimental data. The amplitude distributions obtained from simulations using rate constants derived from individual recorded channels closely matches the experimental data (Fig. S5).

Alternative mechanisms for observed substate gating

We considered that the subconductance events could be artifacts arising from filtering rapid transitions between full open and closed states. Thus, if events are too brief to resolve, a burst of openings may then appear as a

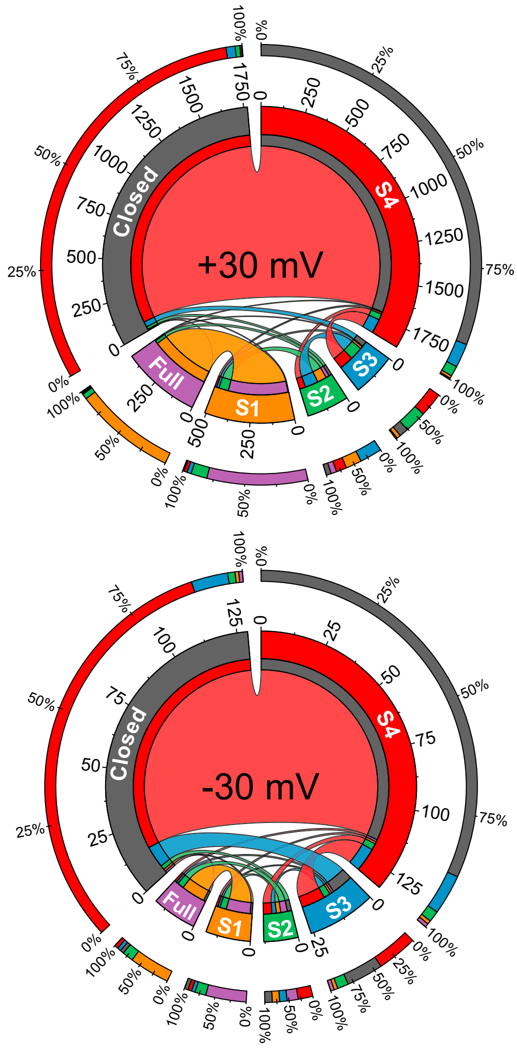

FIGURE 5 Mean transition frequencies between states for voltage-dependent channels. The chord diagrams show the preferred transition routes through the full open, closed, and subconductance states, S1–S4, that are observed at ±30 mV. The mean data for eight voltage-dependent channels is shown. The transition counts are displayed around the central circle where the labeled segments correspond to each of the six states in model M1. States are colored as follows: Closed, gray; S4, red; S3, blue; S2, green; S1, orange; full, magenta. The inner rings show the proportion of transitions out of a state to the other states in the model. Ribbons between pairs of state segments correspond to transitions between the connected states, with ribbon width at a state segment corresponding to the number of transitions out of that state. Outer rings show percentage contributions of each state to the total incoming transitions into any state segment. Note that at −30 mV, there are at least 10 times fewer transitions overall than at +30 mV. At both potentials, a much greater proportion of transitions occur between S4 and the closed state than between any other states, however, at +30 mV, more transitions from S3 and S2 are to S1, S2 or the full open state. To see this figure in color, go online.

subconductance event, the current amplitude of which will depend on open and closed rates. Similar suggestions have been made for various K^+ channels exhibiting subconductance openings (6,30–34). We constructed a model example of a fast-gating mechanism that includes no explicitly defined subconductance states (see Fig. 6 *C*) and examined whether such a model was capable of generating currents that exhibit qualitatively similar sublevels to TRIC-B. The rates were adjusted to generate subconductance states of similar amplitude to the experimentally observed amplitudes, S1–S4. These closed and open states are labeled C_{S1-S4} and O_{S1-S4} according to the amplitude of the subconductance events that are observed following low-pass filtering and resampling (as for the experimental data). In this candidate model, the closing rate, α, is chosen to be 50,000 s^{-1} from O_{s1}, O_{s2}, O_{s3}, and O_{s4} (but these rates could be varied). As in real recordings, full transitions to either the open or closed state were not directly observed in the model output, and instead manifested themselves as small, fast, and noisy subconductance bursts (similar to S1 and S4 behavior in model M1).

Fig. 6 *C* shows a sample of the fast-gating model output highlighting that this model could also reproduce the frequent brief subconductance events that do not subsequently lead to full openings. When the simulated data are analyzed in QuB according to idealization scheme M1 (as for the experimental data), amplitude histograms are generated exhibiting distributions similar to those obtained from experiment (Fig. S7).

Discerning the best model

Model M2 idealized data without severely misclassifying events as sublevel, full open, or closed states (Fig. 2). Lifetimes of the events classified as closed, sublevel, and full open states resulting from M1 or M2 were also similar (Fig. S9). However, simulations using model M2-adapted (Fig. 6 *B*) do not mimic the experimental single-channel current fluctuations as well as the M1-reduced (Fig. 6 *A*) or fast-gating (Fig. 6 *C*) models because M2 cannot account for variations in the subconductance states (Fig. 5) that are a feature of the experimental data. The average full and subconductance currents corresponding to the mean overall fluxes in these states also provide a reasonable approximation of the data. Model M2 is, therefore, ideal for a coarse-grained analysis of TRIC-B Po, for simulation of mean currents, and rates of transitions between the merged subconductance state and the open and closed states but cannot reproduce the intricacies of TRIC-B subconductance gating.

Simulations of the M1-adapted, M1-reduced, and the fast-gating models all produced single-channel current fluctuations that appeared very similar to the experimental data. Average current distributions obtained from simulations using rate constants derived individually from each experimental trace also closely matched the experimental data very well for most channels (see Figs. S4–S7 and Table S3). The Earth Mover's Distance (EMD) (35) was used as an additional measure of the difference (distance) between experimental and simulated amplitude distributions corresponding

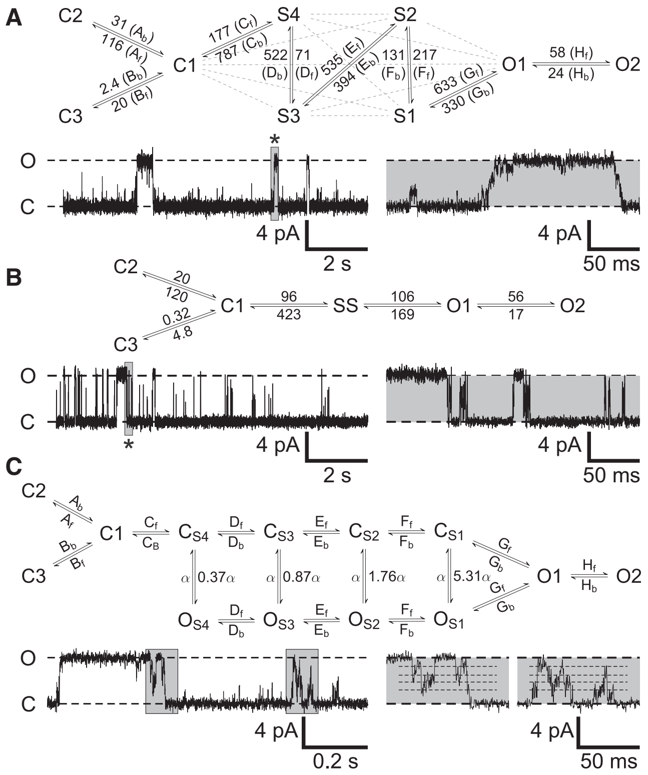

FIGURE 6 Model simulations. (*A*) Model M1 after infrequent transition routes are excluded (M1-reduced). The model is adapted by adding two further closed states (C2, C3) and an extra open state (O2), to reflect the minimum number of closed and open time constants obtained from lifetime analysis. The number of Markov states for each individual subconductance state S1–S4 was kept constant at one. The model scheme is shown with only the transitions whose fitted rates were consistently larger than a threshold of 10^{-3} s^{-1}, across $n = 8$ voltage-dependent channels fitted at +30 mV. The all-to-all connectivity reduces to a linear scheme when connections that are not consistently frequent across all traces are discarded. Discarded connections are shown as gray dotted lines. These transitions did sometimes occur across the eight traces, but were not consistently present in all the channels (for example, see Table S4). Rates were obtained through a simultaneous global fit of the simplified linear scheme (M1-reduced) to eight channel recordings in QuB. These rates (s^{-1}) are given next to their corresponding connections, and are labeled A through H to correspond to the equivalently labeled rates shown in (*C*). An example of simulated openings is shown, and the asterisk indicates where an opening burst highlighted in gray is shown on an expanded timescale (*right*) to show typical features. (*B*) Model M2 after adaptation (M2-adapted) to include the same number of full open and closed states as the M1-reduced model (*above*). It was assumed that transitions O–C occur too infrequently to be included. Rates shown are from a simultaneous global fit in QuB to the data from eight voltage-dependent channels. An example of the model output is shown and the asterisk indicates where an opening burst highlighted in gray is shown on an expanded timescale (*right*) to show typical features. Time and current scales are equivalent to those in (*A*). The simulated traces exhibit superficial similarity to real recordings; however, the model produces a smooth range of subconductance amplitudes around a common mean unlike the subconductance events in experimental recordings. This is obvious in the amplitude histograms of the simulated data (*black line*) shown in Fig. S6. (*C*) Topology of a candidate fast gating scheme, which does not include any explicitly defined subconductance states and only contains fully open (*O*) or fully closed states (*C*). Rapid transitions between coupled pairs of closed and full open states labeled C_{S4}/O_{S4}, C_{S3}/O_{S3}, C_{S2}/O_{S2}, and C_{S1}/O_{S1}, result in observed subconductance state events of amplitude S4, S3, S2, and S1, respectively, after simulated events are low-pass filtered and resampled. Flickery gating arises from rates labeled α, where α is a fast rate (e.g., 50,000 s^{-1}). Transition rates between all remaining states are equivalent to those used in the M1-reduced model and are labeled A–H next to the transitions. A sample of the model output is shown with insets to the right showing subconductance gating on an expanded timescale. Again, amplitude histograms of the simulated data correspond well to experimental recordings (Fig. S7).

to the work (total change in area) required to change the model distribution to exactly match experimental data. Our analysis showed that the output of the M1-reduced model was very similar to that of the M1-adapted model, which maintains all connections between various states. This indicates that, although we may lose some detailed kinetics that are not fully resolvable after data filtering and acquisition, simplification to the M1-reduced model effectively encapsulates key aspects of the gating kinetics. It is likely that states S1–S4 exhibit more than one tau component in their lifetime distributions, although we cannot accurately determine these brief time constants. To better reproduce the full gating kinetics, any model would require more than one Markov state per subconductance.

The fast-gating model yields slightly higher EMD values to those obtained for the M1-reduced and M1-adapted models suggesting that it replicates the data slightly less effectively, especially when simulating currents using globally fitted rates (Table S3). Thus, the large variation in transition rates between channels causes difficulties for producing a one-model-fits-all without further resolution of the time constants in the experimental data. Open channel noise in the fast-gating model is determined by the fast rate α and the imposed level of filtering, constructed from a model of the baseline experimental noise, and is kept constant for all simulations. Baseline noise was adjusted to match individual recordings as in all simulations of our other models. This was constant for all fast-gating model simulations; however, when combined with the intrinsic open channel noise arising from fast gating, the model overestimated substate Po for globally fitted rates. As a result, the fast-gating model does not fit experimental recordings with higher open-channel noise as well as the other models, in which per state open-channel noise distributions are explicitly specified in QuB. We did compare the substate noise observed in experimental recordings to the noise resulting from a range of filtering levels applied to substates in the fast-gating model (Fig. S11) showing that it is unable to completely reproduce the exact noise characteristics observed experimentally.

EMD values for each model were further used with multidimensional scaling (MDS) to compare the performance of each model in relation to the data. MDS is an established tool for visualizing relationships between data by dimensionality reduction (36). The details of our analysis are described in the Supporting Material. Fig. S10 shows how

the results of the EMD analysis of TRIC-B gating can be transformed using MDS by extending pair-wise analysis of EMDs between the amplitude histograms observed in experiments and those obtained by model simulation. Fig. S10 shows that differences between the extended M1-adapted and the linear M1-reduced models are negligible and that these models are closely aligned around the mean of the experimental data. This emphasizes further that the M1-reduced model contains all the important transitions required to provide a comprehensive working model of TRIC-B at +30 mV. The M2 model and the fast-gating models are clearly offset further away from the experimental mean indicating that both provide inferior descriptions of the data to the M1-reduced model.

Voltage-dependent transitions

There are two challenges to extracting the voltage-sensitive pathways. First, the single-channel events are poorly resolved over the voltage range where TRIC-B gating is most sensitive (0–20 mV) and therefore transitions between substates cannot be described. For this reason, model M2 must be used to investigate the main voltage-sensitive steps leading into and out of the subconductance levels. Second, at negative voltages, very few events are observed. Therefore, if one or two long events were observed, this could markedly bias the calculation of transition rates at −30 mV. With these caveats in mind, rates in model M2 were fitted to a full range of experimental data, across voltages from −30 mV to +50 mV, using MIL as implemented in QuB. Too few events occurred at −30 mV to obtain meaningful average rate constants and so only the global fit to the data is shown at this voltage. We found three rates that appeared voltage sensitive and these are shown in Fig. 7. An increase in the rate of transitions from C3 and C2 into C1 when voltage switches from −30 mV to positive potentials was observed. We expect that this would reduce the number of long closings and be the main cause of the increase in frequency of openings. Transition rates C2 > C1 and C3 > C1 will be greatly overestimated at −30 mV because experiments where there are no openings at −30 mV cannot be included thus emphasizing the strong voltage-dependence of these transitions. The transition O1 > S was also sensitive to voltage and was slowed at voltages ≥+20 mV. This would lead to longer full openings above +10 mV. It is likely that additional voltage-sensitive transitions would be revealed if there were not so few openings at −30 mV.

Voltage-dependence of full and subconductance currents

To investigate if subconductance gating of TRIC-B would contribute significantly to physiological K^+ currents across the SR or whether such events would be trivial in comparison to the full openings, we calculated the mean currents resulting from gating in subconductance or full open states. Fig. 8 compares the mean single-channel full open state current (Fig. 8 *A*) and the mean single-channel subconductance state current (Fig. 8 *B*) as a function of holding potential for those experiments where only a single channel was gating in the bilayer (so that openings to subconductance states could be correctly assigned). In Fig. 8 *C*, the percentage contribution of subconductance and full open states to the total current is shown as a function of membrane potential. Extrapolation to physiologically relevant holding potentials (near 0 mV) indicates that subconductance gating of TRIC-B will be responsible for most of the current (~75%).

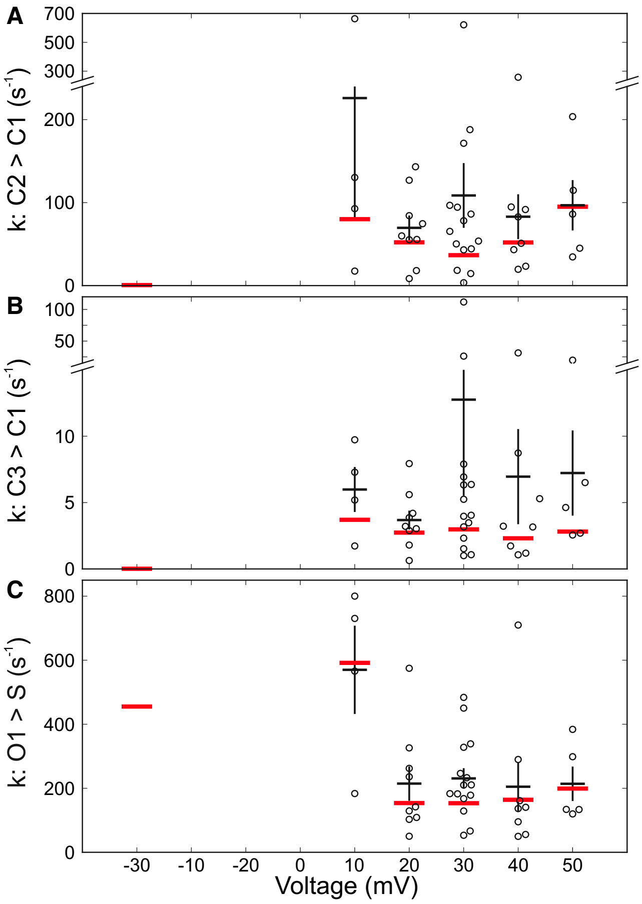

FIGURE 7 Voltage-dependent rates in model M2. Rates were fitted across voltages from −30 to +50 mV, using MIL as implemented in QuB. The graphs of the three rates that appear voltage sensitive (C2 > C1, C3 > C1, and O1 > S) are shown in (*A*), (*B*), and (*C*), respectively. Individual recordings at each voltage (*black circles*) are shown and the horizontal black bars are the mean rates at each voltage (SE). The global fit, where all recordings obtained under a given condition were fitted simultaneously (also in QuB), are plotted as red bars. At −30 mV, only four recordings were available with openings. The number of openings was insufficient to fit rates for individual recordings, therefore global fits only are shown at −30 mV. The following number of recordings were used at each voltage: −30 mV (4), 10 mV (4), 20 mV (9), 30 mV (15), 40 mV (8), and 50 mV (5). To see this figure in color, go online.

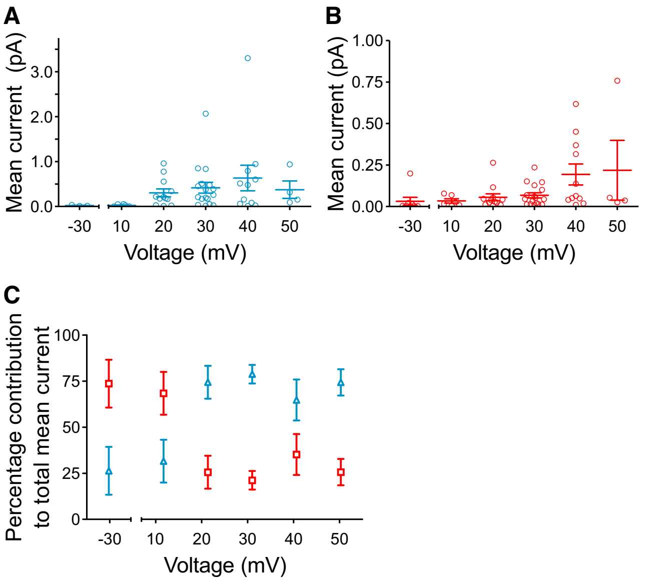

FIGURE 8 Contribution of the full open and subconductance states to the overall current flowing through the channel. The relationship between mean current and voltage is shown for the full open state (*A*), and for openings to any subconductance level (*B*). Mean current contributions due to full and subconductance open states for individual experiments are plotted as open circles. Mean values ± SE are shown (n = 8 (−30 mV), 7 (10 mV), 12 (20 mV), 17 (30 mV), 10 (40 mV), and 4 (50 mV)). Percentage contributions of the full open and combined subconductance states to the total current were then computed for each experiment and were averaged across individual experiments at the various holding potentials (*C*). To see this figure in color, go online.

DISCUSSION

We show that the gating of SR K$^+$ channels is extremely variable from channel to channel but that the main characteristics can be described in a simplified linear gating model that includes four subconducting open states. The Po in the full open state is steeply dependent on voltage over the range 0 to +20 mV but subconductance Po does not entirely mirror this relationship. At +10 mV, the probability of the channels dwelling in a sublevel is still higher than that of opening to the full open state but this is reversed at +20 mV. In terms of the mean K$^+$ flux through each channel, most of the current (~75%) will be due to sublevel openings at ≤+10 mV but at ≥+20 mV, ~75% of current will be due to full open events.

Subconductance openings are especially apparent at −30 mV where the channel opens predominantly to lower amplitude sublevels (S3 and S4). At +30 mV, not only are there more of these low amplitude openings and more full openings but the proportion of openings to higher amplitudes is also increased. Fig. 3, *B* and *C*, suggests that subconductance activity is sensitive to voltage in the range 0 to +10 mV (but current amplitudes are too small to clearly discern sublevels at <+10 mV), whereas voltage change between +10 mV and +20 mV produces the greatest increase in the probability of opening to the full open state. This suggests that a greater amount of transmembrane charge movement is required for TRIC-B to open to the full open state than is necessary for the channel to open to sublevels only.

Subconductance openings in certain voltage-dependent tetrameric ion channels have been suggested to arise from the distinct, noninstantaneous movements of individual subunits of the channel (30,37) under voltage. The movement of the individual subunits gives rise to conformational changes within the conduction pathway such that a full conductance opening is only possible when all four subunits have moved. For TRIC-B, a trimeric protein, such a neat explanation of voltage-dependence would require observation of just two clear sublevels. We observe more apparent sublevels and so can only make loose analogy, but because higher positive holding potentials favor full open states and S1 rather than the lower sublevel openings, the sublevels could represent the incompletely synchronized movements of different subunits or different regions of the channel as it is coerced to open with positive holding potentials. Because open-closed transitions occur via sublevels in 99.99% of transitions, and as transitions between apparent sublevels are very fast (for example, Fig. 1 *Ciii*), we considered that the subconductance events could arise due to very rapid, incompletely resolved transitions between the closed and full open state. The ionic selectivity of the full open and the noisy sublevel of various SR K$^+$ channels (data generally filtered at ~100 Hz) is similar for a range of monovalent cations (3,5,38,39) which would support this theory. Block of the rabbit cardiac SR K$^+$ channel by succinyl choline also pointed toward a gating mechanism to explain the subconductance behavior because the degree of block was reported to be greater for the full open state than for the noisy subconductance state (4). This would be expected if the sublevel was caused by rapid open and closed transitions because the lower Po of the sublevel would reduce the likelihood of a pore blocker entering the conduction pathway. However, in these earlier experiments the recordings were filtered at 100 Hz and hence a single noisy subconductance level only was observed. Only gross changes in subconductance amplitude or duration could be detected under these conditions and so these experiments do not solve this issue. Changing temperature also did not provide clear insight into the underlying mechanisms (8). Over the range 16–37°C, no change in the relative amplitude of the substate to full open state was detected, nor any obvious differences in the durations of time spent in the full open state relative to the subconductance state (8).

In the absence of compelling evidence for the underlying structural changes that lead to subconductance events, we compared the ability of different gating models to portray the interdependence of the subconductance and full open states. Our candidate fast gating model provided

a convincing mimic of experimental single-channel recordings but the amplitude histograms, EMD analysis (Table S3 and Figs. S4–S8) and the use of MDS (Fig. S10) showed greater deviation from experimental data than the linear M1-reduced model that retains four subconductance open states. Slight improvements to the fast-gating model should be possible by fine-tuning the fast rate α, by combining the gating scheme with a more realistic model of open channel noise, or by the inclusion of more open and closed states that are not observed visually. Our attempts at adjusting the fast rate α or the noise characteristics (Fig. S11) did not achieve significant improvements to the model output and so our best fit to the data at +30 mV remains the linear M1-reduced model.

It is important to note that the computed EMD values between each model and its corresponding experimental data were lower than those observed within the experimental data (Table S3). In other words, when the mean EMD for each experimental recording compared to the other five experiments was calculated, the EMD values ranged from 0.355 to 0.417, which are much higher than the EMD between any of the models and the data. This interchannel level of complexity reduces the likelihood of converging to a gating scheme that accurately describes all aspects of TRIC-B gating. Nonetheless, we show that the M2 model provides a good approximation of transitions between the merged subconductance state and the full open and closed states. The linear M1-reduced model enables integration of the most frequent transitions between subconducting open states, S1–S4, into the model and provides significantly improved simulation of experimental data. It is clear from global fitting of model M2 that important voltage-dependent rates are C3 > C1, C2 > C1 and O1 >S. These transitions are a key element of the close correspondence between the linear gating simplification of model M1 and experimental data. However, simplified substate transitions in M1-reduced are drawn from data analyzed at +30 mV, and therefore the model may not be generalizable across all voltages. Improved resolution of the substate gating is necessary before we can comment on the voltage-sensitive transitions between the different subconducting open states.

CONCLUSIONS

From the relationship between voltage and subconductance Po (Fig. 3 *B*), although we cannot measure channel openings at 0 mV in symmetrical conditions, we can extrapolate the data to suggest that TRIC-B is normally gating to subconductance open states at potentials close to 0 mV with only rare openings to the full open state. In the cell, as there is thought to be no large resting membrane potential across the SR (40), TRIC-B would be expected to be gating in a low Po mode and primarily to subconductance open states unless there was buildup of negative charge in the SR relative to the cytosol. The openings of many channels, even to subconductance levels, would allow equilibration of monovalent cations across the SR membrane (15) and would also contribute charge compensating current at any time that there was Ca^{2+} flux across the SR although, obviously, the SR K^+ flux would be more limited than if channels opened with high Po to the full open state. We have focused on the voltage-dependent channels in this study although ~11% of channels are voltage-independent. Because voltage-independent channels exhibited high Po at the voltages monitored (12), and since these channels may physiologically constitute a proportion of the channels in the cell, this adds to the likelihood that, at least some TRIC-B channels will be open at SR membrane potentials near 0 mV. The SR membrane composition is more complex than our simple neutral bilayer and it will be important to evaluate how the interrelationship between subconducting and full open states is altered at 37°C and within a physiological environment, particularly with a phosphatidylserine component to the membrane, because negative charge has been shown to affect SR K^+ channel function (4,41,42). Other factors including temperature, pH, and divalent cations (1,21,22) may also be important. For example, rapid and large changes in cytosolic and luminal [Ca^{2+}] take place during excitation-contraction coupling in both cardiac and skeletal muscle and could potentially influence channel gating. Widely inconsistent effects of cytosolic and luminal Ca^{2+} on SR K^+ channel gating have been reported (21,43,44). This may be due to the difficulty of distinguishing between subconductance gating and block of the channel by Ca^{2+}, especially when events are poorly resolved, thus showing the need to investigate SR K^+ channel behavior at high resolution and when only a single channel is present in the bilayer.

SUPPORTING MATERIAL

Supporting Materials and Methods, Supporting Results, 11 figures, and four tables are available at http://www.biophysj.org/biophysj/supplemental/S0006-3495(15)00602-5.

AUTHOR CONTRIBUTIONS

T.I., M.N., and H.T. produced and characterized the TRIC-A knockout mice and provided tissue. E.V. and F.O'B. isolated SR membrane vesicles. A.M., E.V., and F.O'B. performed experiments, analyzed data, and produced figures. A.M., K.T.-A., and R.S. developed models of gating. A.M. performed simulations. A.M. and R.S. wrote the article. All authors discussed the results and commented on the article.

ACKNOWLEDGMENTS

This work was supported by the British Heart Foundation, the Japan Society for the Promotion of Science (Core to Core Program), and the Engineering and Physical Sciences Research Council (A.M. by grant EP/E501214/1, KT-A by grant EP/L000296/1).

SUPPORTING CITATIONS

References (45–48) appear in the Supporting Material.

REFERENCES

1. Coronado, R., R. L. Rosenberg, and C. Miller. 1980. Ionic selectivity, saturation, and block in a K+-selective channel from sarcoplasmic reticulum. *J. Gen. Physiol.* 76:425–446.
2. Labarca, P. P., and C. Miller. 1981. A K+-selective, three-state channel from fragmented sarcoplasmic reticulum of frog leg muscle. *J. Membr. Biol.* 61:31–38.
3. Tomlins, B., A. J. Williams, and R. A. Montgomery. 1984. The characterization of a monovalent cation-selective channel of mammalian cardiac muscle sarcoplasmic reticulum. *J. Membr. Biol.* 80:191–199.
4. Gray, M. A., R. A. P. Montgomery, and A. J. Williams. 1985. Asymmetric block of a monovalent cation-selective channel of rabbit cardiac sarcoplasmic reticulum by succinyl choline. *J. Membr. Biol.* 88:85–95.
5. Tomlins, B., and A. J. Williams. 1986. Solubilisation and reconstitution of the rabbit skeletal muscle sarcoplasmic reticulum K+ channel into liposomes suitable for patch clamp studies. *Pflugers Arch.* 407:341–347.
6. Hill, Jr., J. A., R. Coronado, and H. C. Strauss. 1990. Open-channel subconductance state of K+ channel from cardiac sarcoplasmic reticulum. *Am. J. Physiol.* 258:H159–H164.
7. Rousseau, E., H. Chabot, …, B. Muller. 1992. Reconstitution and regulation of cation-selective channels from cardiac sarcoplasmic reticulum. *Mol. Cell. Biochem.* 114:109–117.
8. Shen, W. K., R. L. Rasmusson, …, H. C. Strauss. 1993. Voltage and temperature dependence of single K+ channels isolated from canine cardiac sarcoplasmic reticulum. *Biophys. J.* 65:747–754.
9. Picher, M., A. Decrouy, and E. Rousseau. 1996. Conducting and voltage-dependent behaviors of potassium ion channels reconstituted from diaphragm sarcoplasmic reticulum: comparison with the cardiac isoform. *Biochim. Biophys. Acta.* 1279:93–103.
10. Yazawa, M., C. Ferrante, …, H. Takeshima. 2007. TRIC channels are essential for Ca2+ handling in intracellular stores. *Nature.* 448:78–82.
11. Pitt, S. J., K.-H. Park, …, R. Sitsapesan. 2010. Charade of the SR K+-channel: two ion-channels, TRIC-A and TRIC-B, masquerade as a single K+-channel. *Biophys. J.* 99:417–426.
12. Venturi, E., A. Matyjaszkiewicz, …, R. Sitsapesan. 2013. TRIC-B channels display labile gating: evidence from the TRIC-A knockout mouse model. *Pflugers Arch.* 465:1135–1148.
13. Zhao, X., D. Yamazaki, …, J. Ma. 2010. Ca2+ overload and sarcoplasmic reticulum instability in tric-a null skeletal muscle. *J. Biol. Chem.* 285:37370–37376.
14. Yamazaki, D., Y. Tabara, …, H. Takeshima. 2011. TRIC-A channels in vascular smooth muscle contribute to blood pressure maintenance. *Cell Metab.* 14:231–241.
15. Tao, S., D. Yamazaki, …, H. Takeshima. 2013. Facilitated hyperpolarization signaling in vascular smooth muscle-overexpressing TRIC-A channels. *J. Biol. Chem.* 288:15581–15589.
16. Yamazaki, D., S. Komazaki, …, H. Takeshima. 2009. Essential role of the TRIC-B channel in Ca2+ handling of alveolar epithelial cells and in perinatal lung maturation. *Development.* 136:2355–2361.
17. Shaheen, R., A. M. Alazami, …, F. S. Alkuraya. 2012. Study of autosomal recessive osteogenesis imperfecta in Arabia reveals a novel locus defined by TMEM38B mutation. *J. Med. Genet.* 49:630–635.
18. Volodarsky, M., B. Markus, …, O. S. Birk. 2013. A deletion mutation in TMEM38B associated with autosomal recessive osteogenesis imperfecta. *Hum. Mutat.* 34:582–586.
19. Rubinato, E., A. Morgan, …, F. Faletra. 2014. A novel deletion mutation involving TMEM38B in a patient with autosomal recessive osteogenesis imperfecta. *Gene.* 545:290–292.
20. Sitsapesan, R., R. A. Montgomery, …, A. J. Williams. 1991. Sheep cardiac sarcoplasmic reticulum calcium-release channels: modification of conductance and gating by temperature. *J. Physiol.* 434:469–488.
21. Miller, C. 1978. Voltage-gated cation conductance channel from fragmented sarcoplasmic reticulum: steady-state electrical properties. *J. Membr. Biol.* 40:1–23.
22. Labarca, P., R. Coronado, and C. Miller. 1980. Thermodynamic and kinetic studies of the gating behavior of a K+-selective channel from the sarcoplasmic reticulum membrane. *J. Gen. Physiol.* 76: 397–24.
23. Qin, F. 2004. Restoration of single-channel currents using the segmental k-means method based on hidden Markov modeling. *Biophys. J.* 86:1488–1501.
24. Krzywinski, M., J. Schein, …, M. A. Marra. 2009. Circos: an information aesthetic for comparative genomics. *Genome Res.* 19:1639–1645.
25. Qin, F., A. Auerbach, and F. Sachs. 1996. Estimating single-channel kinetic parameters from idealized patch-clamp data containing missed events. *Biophys. J.* 70:264–280.
26. Qin, F., A. Auerbach, and F. Sachs. 1997. Maximum likelihood estimation of aggregated Markov processes. *Proc. Biol. Sci.* 264:375–383.
27. Colquhoun, D., and F. J. Sigworth. 2009. Fitting and statistical analysis of single-channel records. *In* Single-Channel Recording. B. Sakmann and E. Neher, editors. Springer, NY, pp. 483–587.
28. Blatz, A. L., and K. L. Magleby. 1986. Correcting single channel data for missed events. *Biophys. J.* 49:967–980.
29. Gillespie, D. T. 1976. A general method for numerically simulating the stochastic time evolution of coupled chemical reactions. *J. Comput. Phys.* 22:403–434.
30. Chapman, M. L., H. M. VanDongen, and A. M. VanDongen. 1997. Activation-dependent subconductance levels in the drk1 K channel suggest a subunit basis for ion permeation and gating. *Biophys. J.* 72:708–719.
31. Shang, L., S. V. Ranson, and S. J. Tucker. 2009. Kir5.1 underlies long-lived subconductance levels in heteromeric Kir4.1/Kir5.1 channels from *Xenopus tropicalis*. *Biochem. Biophys. Res. Commun.* 388:501–505.
32. Zheng, J., and F. J. Sigworth. 1998. Intermediate conductances during deactivation of heteromultimeric *Shaker* potassium channels. *J. Gen. Physiol.* 112:457–474.
33. Bezanilla, F. 2005. The origin of subconductance levels in voltage-gated K+ channels. *J. Gen. Physiol.* 126:83–86.
34. Ferguson, W. B., O. B. McManus, and K. L. Magleby. 1993. Opening and closing transitions for BK channels often occur in two steps via sojourns through a brief lifetime subconductance state. *Biophys. J.* 65:702–714.
35. Cohen, S., and L. Guibas. 1997. The Earth Mover's Distance: Lower Bounds and Invariance under Translation. DTIC Document.
36. Borg, I., and P. J. F. Groenen. 2005. Modern Multidimensional Scaling. Springer-Verlag, NY.
37. Chapman, M. L., and A. M. J. VanDongen. 2005. K channel subconductance levels result from heteromeric pore conformations. *J. Gen. Physiol.* 126:87–103.
38. Fox, J. A. 1985. Conductance and selectivity properties of a substate of the rabbit sarcoplasmic reticulum channel. *Biophys. J.* 47:573–576.
39. Hill, Jr., J. A., R. Coronado, and H. C. Strauss. 1989. Potassium channel of cardiac sarcoplasmic reticulum is a multi-ion channel. *Biophys. J.* 55:35–45.
40. Garcia, A. M., and C. Miller. 1984. Channel-mediated monovalent cation fluxes in isolated sarcoplasmic reticulum vesicles. *J. Gen. Physiol.* 83:819–839.
41. Anzai, K., C. Takano, …, Y. Kirino. 1994. Asymmetrical lipid charge changes the subconducting state of the potassium channel from sarcoplasmic reticulum. *Biochem. Biophys. Res. Commun.* 199:1081–1087.
42. Bell, J. E., and C. Miller. 1984. Effects of phospholipid surface charge on ion conduction in the K+ channel of sarcoplasmic reticulum. *Biophys. J.* 45:279–287.

43. Uehara, A., M. Yasukochi, and I. Imanaga. 1994. Calcium modulation of single SR potassium channel currents in heart muscle. *J. Mol. Cell. Cardiol.* 26:195–202.

44. Liu, Q. Y., and H. C. Strauss. 1991. Blockade of cardiac sarcoplasmic reticulum K+ channel by Ca2+: two-binding-site model of blockade. *Biophys. J.* 60:198–203.

45. Massey, F. J. 1951. The Kolmogorov-Smirnov test for goodness of fit. *J. Am. Stat. Assoc.* 46:68–78.

46. Rosales, R. A., W. J. Fitzgerald, and S. B. Hladky. 2002. Kernel estimates for one- and two-dimensional ion channel dwell-time densities. *Biophys. J.* 82:29–35.

47. Qin, F. 2007. Principles of single-channel kinetic analysis. *Methods Mol. Biol.* 403:253–286.

48. Colquhoun, D., and A. G. Hawkes. 2009. A Q-matrix cookbook. *In* Single-Channel Recording. B. Sakmann and E. Neher, editors. Springer, NY, pp. 589–633.

Sub-conductance gating and voltage-sensitivity of sarcoplasmic reticulum K^+ channels: a modeling approach

Supporting Material

Antoni Matyjaszkiewicz[1,2], Elisa Venturi[3], Fiona O'Brien[3], Tsunaki Iida[4], Miyuki Nishi[4], Hiroshi Takeshima[4], Krasimira Tsaneva-Atanasova[5] and Rebecca Sitsapesan[3,*].

[1]Bristol Centre for Complexity Sciences, University of Bristol, UK
[2]Department of Engineering Mathematics, University of Bristol, UK
[3]Department of Pharmacology, University of Oxford, UK
[4]Graduate School of Pharmaceutical Sciences, Kyoto University, Japan
[5]College of Engineering, Mathematics and Physical Sciences University of Exeter

Table of Contents

Supporting Methods and Results **3**
Suitability of M1 and M2 3
Computation of simulated data and use of mean currents 4
Computing the Earth Mover's Distance (EMD) 5
Further analysis involving the EMD: multidimensional scaling 5
Fast gating model: effects of filtering on sub-state noise 7
Supporting References **8**
Supporting Figures **9**
Figure S1. Transition pathways between open and closed channel states 9
Figure S2. Steady-state channel gating behaviour 10
Figure S3. Model M1-adapted (adapted to include multiple full open and closed states) and typical examples of simulated data 11
Figure S4. Amplitude histograms for individual experimental recordings, with corresponding simulations of model M1-adapted 12
Figure S5. Amplitude histograms for individual experimental recordings, with corresponding simulations of model M1-reduced 13
Figure S6. Amplitude histograms for individual experimental recordings, with corresponding simulations of model M2-adapted 14
Figure S7. Amplitude histograms for individual experimental recordings, with corresponding simulations of the candidate fast-gating model 15
Figure S8. Mean amplitude histograms of model simulations with rates fitted globally to experimental data 16
Figure S9. Comparison of lifetime distributions arising from idealisation in QuB with M1 or M2 using the kernel density method 17
Figure S10. Application of multidimensional scaling to EMDs between models and data 18
Figure S11. Comparison of filtering effects on experimentally observed sub-states, and sub-states generated by the fast-gating model. 19
Supporting Tables **20**
Table S1. Lifetime analysis for full open and closed states of single voltage-dependent channels 20
Table S2. Observed direct transitions between the closed and full open state 20
Table S3. Comparison of experimentally observed mean single-channel currents with simulated mean currents derived from the various models 21
Table S4. Transition rates for model M1-adapted 22

Supporting Methods and Results

Suitability of M1 and M2

Model suitability was assessed using 8 representative recordings where only single channels were gating in the bilayer. TRIC-B channels exhibit variable gating kinetics (1) so we used only channels exhibiting voltage-dependent behaviour. Fitted events from idealisations derived using model M1 in QuB (2) were checked visually to assess whether sub-state events from QuB matched those that could be classified by eye. For holding potentials of +30 mV and above, where the amplitude of openings was sufficiently large to resolve distinct sub-conductance states, visual inspection revealed a close match to events fitted in QuB. However, smaller amplitude sub-conductance state openings, for example, those observed at holding potentials of 10 or 20 mV, were not easily differentiable from background noise.

The more coarse-grained idealisations fitted using model M2 were compared visually both to the data and to idealisations produced by model M1. An initial comparison of Po obtained from amplitude histograms indicated that model M2 was able to correctly identify regions where the channel was fully open or closed, and distinguish these from regions of sub-conductance gating. Without stripping events below 0.6 ms, idealisations produced by M2 had a mean overlap of 99.0% ± 0.7 (SD; n=8) with idealisations produced using M1. Overlap was defined as the per-sample correspondence, for each experimental recording, between the idealisations produced by each model. A digitised sample point of an experimental recording classified as the same state by both M1 and M2 would count as an overlap, but a sample classified as one state (open, for example) by M1 and as another state (closed, for example) by M2 would not count as an overlap between the two models. After removal of events < 0.6 ms, as in all further analysis, idealisations produced by M2 had a mean overlap of 99.3% ± 0.6 (SD; n=8) with idealisations produced using M1. Considering sublevel events only, on average 95.0% ± 3.5 (SD; n=8) of sublevel events classified by M2 overlapped with those classified by M1.

Lifetime distributions resulting from idealisations computed by both models were compared using the two-sample Kolmogorov-Smirnov (K-S) test (3). P=0.05 was chosen as a suitable level at which to reject the null hypothesis (that the two distributions, M1 and M2, come from the same underlying distribution). M1 idealisations were pre-processed to make them comparable to those produced by M2, since M1 contains multiple sub-conductance states while M2 contains only a single merged sub-conductance state. M1 sublevel events (S4 to S1) were all assigned to a single merged state, as in M2, and consecutive events of the new merged sublevel were combined into single longer events. Computed K-S distance values, D, and the corresponding critical distance for P=0.05 for each set of events are shown, for two representative experimental recordings in Fig. S9. The idealisations of all voltage-dependent channels were compared in this manner. Sparse lifetime distributions with fewer than 250 events were not included in the comparison. For all of the distributions analysed, D was lower than the critical value for P=0.05, meaning that the lifetime distributions arising from idealisations computed using M1 and M2 were not significantly different at this level. Additional visual inspection of the distributions (smoothed using the kernel density estimation method introduced by Rosales et al. (4) to reduce binning errors in regions with few samples) indicated that all distributions considered here had similar shape and number of components (Fig. S9). Model M2 is therefore comparable in classification performance to Model M1 in discriminating between currents belonging to full open, closed or sub-conducting open states but not between individual sub-conductance open states.

Computation of simulated data and use of mean currents

The mean currents obtained from voltage-dependent experimental recordings (from QuB) were used for comparing models and were calculated as described in the main Methods. For simulated data, state amplitudes and standard deviation were known *a priori* and were input to each model. The mean currents were computed directly from amplitude histograms by calculating the centroid of each amplitude distribution as follows: We can define a finite amplitude distribution x over the points x_i, representing currents, with corresponding weights w_i, such that

$$x = \{(x_1, w_1), (x_2, w_2), \dots, (x_n, w_n)\},$$

where n is the number of points in the distribution. The total weight of the distribution is

$$w_\Sigma = \sum_{j=1}^{n} w_j.$$

The mean current is then the centroid $\bar{x}$ of the distribution,

$$\bar{x} = \frac{\sum_{j=1}^{n} w_j x_j}{w_\Sigma}.$$

Model simulations were performed as described in the main text. Convergence criteria were the relative change in the centroid of each distribution, and the relative sum of squared residuals, between consecutive iterations of the simulation (30 seconds of simulated data for the fast-gating model, and 60 seconds of simulated data for all the remaining models per iteration) since both the mean current and the shape of the distribution were used to assess model correspondence to experimental data. The smallest resolvable difference in digitised current is 10^{-3} pA. This value was chosen as an appropriate relative error tolerance for changes in mean current (centroid) computed from each distribution. Likewise, a value of 10^{-6} pA^2 was chosen as the relative error tolerance for the sum of squared residual differences. Convergence occurred in under 1000 iterations, corresponding to a total of between 500 and 1000 min of simulated recordings for a given condition, depending on the model type. For the M1-adapted, M1-reduced, and M2 models, where state amplitudes and corresponding noise distributions are explicitly parameterised, it is possible to analytically compute amplitude distributions by calculating the equilibrium Po distribution for a given set of rates. Given a transition rate matrix, $\boldsymbol{Q}$, the transition probability matrix is defined as $\boldsymbol{P} = e^{\boldsymbol{Q}\Delta \boldsymbol{t}}$, with entries $p_{i,j} = \Pr(X_{n+1} = j \mid X_n = i)$, and $\Delta \boldsymbol{t}$ the sampling interval of the simulation (5, 6). For a given probability matrix, $\boldsymbol{P}$, the equilibrium Po distribution, $\boldsymbol{\pi}$, a row vector whose entries correspond to the equilibrium Po of each Markov state in the model, is, by definition, unchanged under multiplication with $\boldsymbol{P}$, so $\boldsymbol{\pi P} = \boldsymbol{\pi}$ (5, 6). This is related to the standard eigenvector problem and so we can compute $\boldsymbol{\pi}$ by calculating the eigenvalues and left eigenvectors of $\boldsymbol{P}$, so that

$$\boldsymbol{\pi} = \frac{\boldsymbol{v}}{\sum_i v_i}$$

where $\boldsymbol{v}$ is a left eigenvector of $\boldsymbol{P}$ with a corresponding eigenvalue of 1. The analytical current amplitude distribution is then simply a sum of Gaussian functions whose amplitudes correspond to the equilibrium Po, and whose means and standard deviations correspond to the current amplitude and noise for each state, as parameterised from each experimental recording. The analytical distributions were used to confirm that the simulated data were converging correctly. The simulated data, (rather than the analytically derived amplitude distributions) were used when comparing models since the

properties of *observed* 'sub-conductance levels' cannot be calculated analytically in the case of the fast-gating model

Computing the Earth Mover's Distance (EMD)

The same simulated amplitude distributions as those discussed above were used when computing Earth Movers Distance (EMD) to experimental recordings for each model. EMD was computed as described in (7).

In order to compute EMD we need to calculate the cumulative distribution function (CDF) of x defined as:

$$\mathrm{CDF}_x(t) = \begin{cases} 0 & \text{if } t \in (-\infty,\ x_1) \\ \sum_{i=1}^{k} w_i & \text{if } t \in [x_k,\ x_{k+1}), 1 \le k \le n-1. \\ w_\Sigma = \sum_{i=1}^{n} w_i & \text{if } t \in [x_n, \infty) \end{cases}$$

If we compute two CDFs, one for a model and one for a dataset, we can compute the EMD between the two distributions as

$$\mathrm{EMD}(\mathrm{data}, \mathrm{model}) = \frac{E}{w_\Sigma},$$

where E is the total work performed in the optimal transfer of area (mass) between the two CDFs. In our case, the problem is one-dimensional (a univariate distribution) and thus:

$$E = \sum_{k=1}^{n} (x_{k+1} - x_k)|\mathrm{CDF}_{\mathrm{data}}(x_k) - \mathrm{CDF}_{\mathrm{model}}(x_k)|.$$

This notation assumes that the $\mathrm{CDF}_{\mathrm{data}}(x)$ and $\mathrm{CDF}_{\mathrm{model}}(x)$ are defined over the same shared set of co-ordinates $X = [x_1,\ \dots\ , x_n]$ i.e., have the same support, which they do in our data.

Further analysis involving the EMD: multidimensional scaling

We use multidimensional scaling (MDS) to compare the performance of each proposed model in relation to the data. MDS allows us to represent the gating behaviour of TRIC-B channel as points in an abstract geometric space. MDS is a well-established tool in data visualisation and data mining (8). It allows for reduction in dimensionality of the data and visualisation of relations between the objects under investigation while preserving as much information as possible. Since the EMD is a metric in the space of TRIC-B channel gating behaviour (captured by the pdfs corresponding to amplitude histograms), we use classical MDS implemented in MATLAB (The MathWorks, Inc.) as the command ‘cmdscale’ (http://uk.mathworks.com/help/stats/cmdscale.html).

We first computed the EMDs between all pdfs (experimental, as well as model simulations), which correspond to individual channels, for all pairwise combinations of data-data, model-data, and model-model amplitude histograms. Then we used the computed EMDs to construct a distance matrix **D**. Specifically we generated an n*n distance matrix containing all possible pairwise interactions (where n = n(data) + n(model)*n(model fit type)). Each row of this matrix was assigned to a different amplitude histogram (and hence belongs to a specific channel) and contains EMDs between this amplitude histogram and all the other amplitude histograms. For instance, cell (2,3) contains the EMD

between second and third amplitude histograms in our dataset. Since the EMD is a metric, matrix **D** has zeros on the diagonal and is symmetric.

Next, we used the MDS to transform matrix **D** into coordinates of points in the "dissimilarities" space. In particular, following MDS, each amplitude histogram is represented as a single point in two-dimensional space. Here we used only the first two dimensions of the "dissimilarities" space, which turn out to be sufficient for the purpose of our analysis since the first two eigenvalues computed in the MDS were within one order of magnitude, while the remaining eigenvalues were significantly smaller. Points corresponding to data-derived and model-generated amplitude histograms in these first two dimensions are shown in Figure S10.

In summary, MDS allows us to extend pair-wise analysis of distances between amplitude histograms observed in experiments and simulated by the models and thus gain further insight into the performance of our modelling. Furthermore, using MDS guarantees that the Euclidean distances between elements in the "dissimilarity" space are a good approximation of the EMDs between amplitude histograms.

Projecting the amplitude histograms corresponding to all models and data in the two most significant dimensions after MDS illustrates some notable distinctions between model types and fits (Figure S10).

Firstly, key qualitative differences between models are preserved, and can be visualised in the new reduced space generated by MDS. Specifically, one can see that 'individually' fitted model rates (squares, Figure S10) i.e., those where a set of rates were fitted to a single experimental recording, are reasonably close to corresponding data points (blue circles, Figure S10). This is particularly true for the M1 full (red points) and linear (yellow) models, less so for the M2 model (purple), and barely true for the fast-gating model (green). This is consistent with the fact that the simulated data from the M2 and fast gating models exhibit clear visual differences in amplitude distributions to the data (for example, compare the amplitude distributions in Figures S4 – S7 for individually fitted rates, and Figure S8 for globally fitted rates). In the case of model M2, this is due to all sub-state amplitudes being merged into one; for the fast-gating model, this is a result of sub-state noise being invariant of the data to which the model rates were fitted. Variations in the differing abilities of the models to reproduce observed data are also highlighted when we look at the average Euclidean distances between models and data in the reduced co-ordinate space. For all models, the distance between individual fits (squares) and their corresponding data points (circles) is much smaller than the distance between global fits (stars) and their corresponding data points (circles). Full and linear models are the closest to the data, with the fast-gating model over four times further away on average.

Conversely, the points corresponding to globally fitted rates (stars in Figure S10) are much closer to the mean data distribution (black diamond). Finally, some important qualitative distinctions between model types can be made by considering the points corresponding to globally fitted rates for each model type. The globally fitted linear M1 model is almost exactly equivalent to the full M1 model, indicating that the reduced kinetic scheme performs favourably in reproducing observed behaviour, compared to the original scheme. These two models are also closest to the data mean in the space generated by MDS, crossing directly over it, which the other models do not. The M2 model appears to be flexible in reproducing a range of different observed data, however it is not as close to the experimental points on average as the M1 models. The fast gating models' points are tightly clustered. This makes sense as the model is less flexible than the others; it is unable to take into account per-data variation in sub-state noise, meaning it cannot reproduce all the variations in sub-state amplitude

distributions observed across our data. However, on average it is still not much further from the mean of the data than the M2 model.

We can conclude that although M2 is good for data classification, and simulation of average currents, it does not accurately capture the complexities of observed sub-state gating kinetics. The M1 models are only good for classifying data when there is a good signal-to-noise ratio. However they are able to reproduce observed data, after fitting of kinetic rates, more closely than the other models. In addition, the reduction of the full M1 scheme to a linear kinetic scheme doesn't appear to increase its distance from the observed data. The fast gating model does accurately reproduce some kinetic aspects of the observed data (since inter-sub-state kinetics are based on those fitted by the linear M1 model). However it cannot reproduce all of the variation in observed experimental data, particularly that relating to sub-state noise. It is the furthest of all our models from the experimental data.

Fast gating model: effects of filtering on sub-state noise

Eight single channel recordings obtained at +30 mV were analysed, and compared to simulated data obtained from the fast gating model (Figure 6C). Analysis of a representative recording is shown in Figure S11. Similar trends were observed across all 8 recordings analysed.

Experimental recordings were idealised using model M1, after filtering at 1 kHz, as discussed in the main material. Each raw experimental recording was then filtered using a Gaussian filter at multiple cut-off thresholds in the range 250–1500 Hz in 250 Hz increments. The data points corresponding to each sub-state in the previously obtained idealisation were then extracted from the filtered data (only events lasting longer than 1 ms were included to reduce the filter's effects on short events). The fast-gating model was simulated as discussed in the main material, with fast rates calibrated to generate appropriate amplitudes for each of the four sub-states, and then filtered in the same way as the experimental data. Distributions of simulated (blue) and experimentally obtained (red) data under a range of filtering thresholds are shown in Figure S11.

For the small amplitude sub-state S4, the amplitude distributions for both model and data have a low median amplitude, with the distributions exhibiting a tail into higher current amplitudes. The large amplitude sub-state exhibits the converse behaviour: a high median with a tail into smaller amplitude currents. This is consistent with what would be expected from a flickery opening/closing. The current distribution of the mid amplitude sub-state S3 exhibits little bias in either direction in simulated data, which is to be expected since transitions are occurring in an approximately equal ratio of closed:open in order to generate the intermediate current amplitude that is observed. Changes in the noise distributions for experimental data are consistent with what is seen in simulations of the fast-gating model, meaning that we cannot entirely discount the possibility of a fast gating mode between the closed and open state of the channel being the mechanism behind observed sub-conductances. Furthermore, increasing the filtering cut-off threshold clearly leads to greater spread and hence variability in current amplitudes observed in both the data and model. However, the fast-gating model is unable to completely reproduce the exact noise characteristics observed in experimental data (Figure S11).

Supporting References

1. Venturi, E., A. Matyjaszkiewicz, S.J. Pitt, K. Tsaneva-Atanasova, M. Nishi, D. Yamazaki, H. Takeshima, and R. Sitsapesan. 2013. TRIC-B channels display labile gating: evidence from the TRIC-A knockout mouse model. Pflugers Arch. 465: 1135–48.

2. Qin, F. 2004. Restoration of single-channel currents using the segmental k-means method based on hidden Markov modeling. Biophys. J. 86: 1488–501.

3. Massey, F.J. 1951. The Kolmogorov-Smirnov Test for Goodness of Fit. J. Am. Stat. Assoc. 46: 68–78.

4. Rosales, R.A., W.J. Fitzgerald, and S.B. Hladky. 2002. Kernel estimates for one- and two-dimensional ion channel dwell-time densities. Biophys. J. 82: 29–35.

5. Qin, F. 2007. Principles of single-channel kinetic analysis. Methods Mol. Biol. 403: 253–86.

6. Colquhoun, D., and A.G. Hawkes. 2009. A Q-Matrix Cookbook. In: Sakmann B, E Neher, editors. Single Channel Recording. Springer. pp. 589–633.

7. Cohen, S., and L. Guibas. 1997. The earth mover's distance: lower bounds and invariance under translation. DTIC Document.

8. Borg, I., and P.J.F. Groenen. 2005. Modern Multidimensional Scaling. Springer-Verlag New York.

Supporting Figures

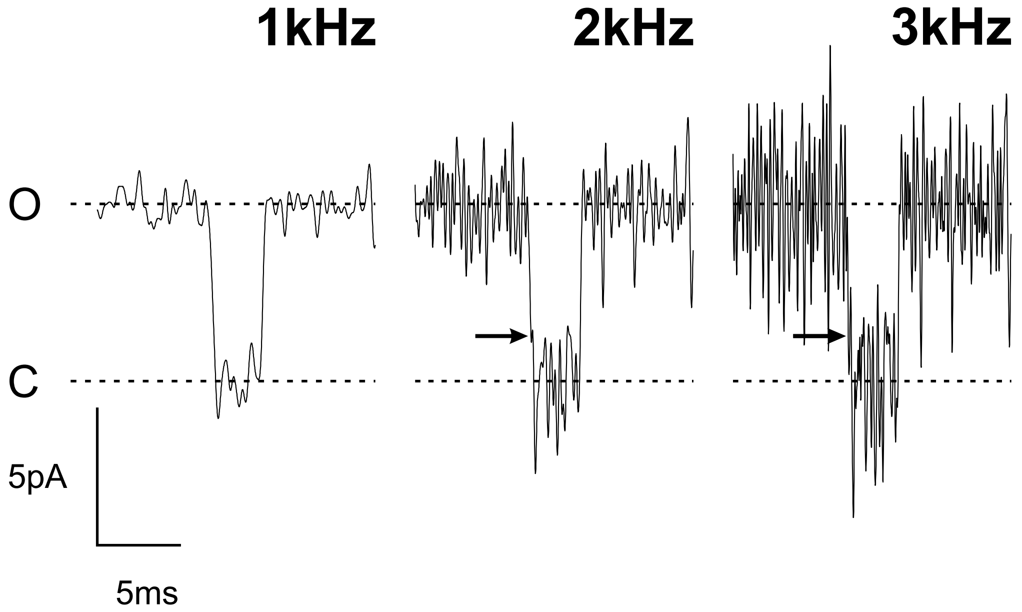

Figure S1. Transition pathways between open and closed channel states
Occasionally we observed opening or closing events that did not, when filtered at 1 kHz, appear to transition through a sub-conducting state (see Table S2). However, upon increasing filter cut-off frequency, we then found evidence for possible sub-conductance events. Representative current fluctuations are shown for an open-closed transition that initially appears not to have passed through any intermediate sub-conductance. The holding potential was +30 mV. Arrows indicate the possible sub-conductance levels that emerge as the filtering level is altered from 1 kHz (left), to 2 kHz (middle) and 3 kHz (right).

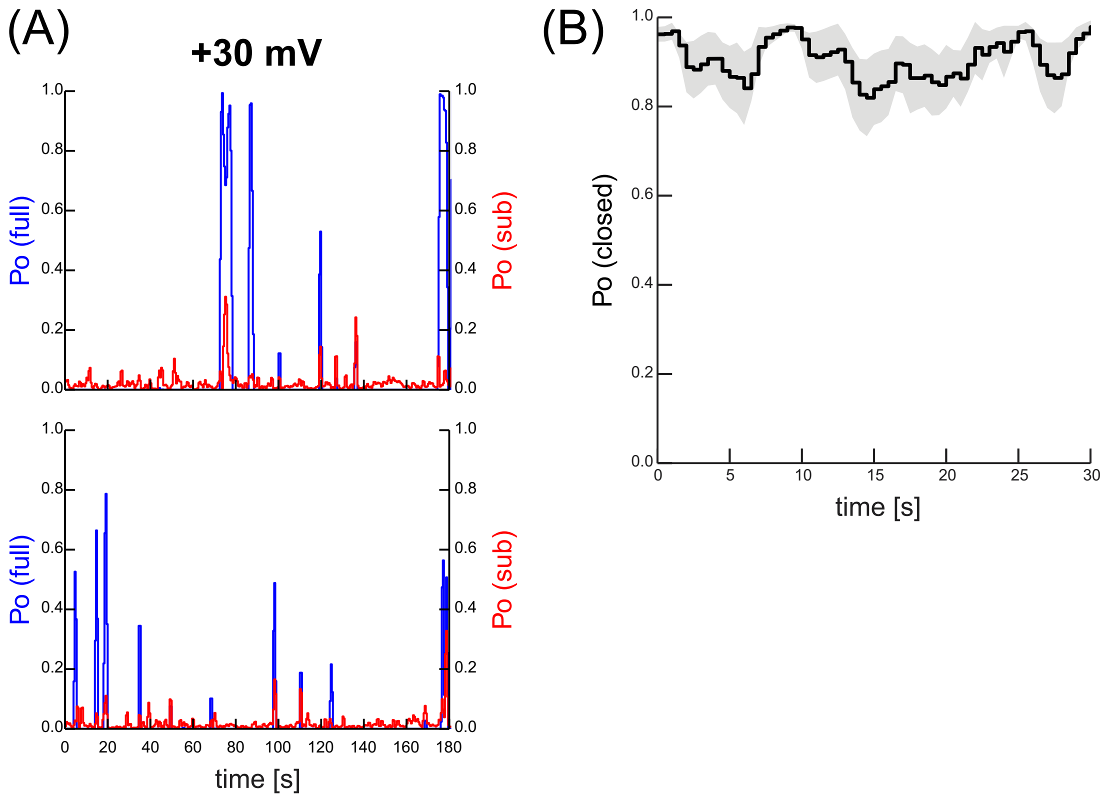

Figure S2. Steady-state channel gating behaviour

(A) Data from two typical experiments are shown in which the holding potential was held constant for 180 s at +30 mV. Single-channel records were divided into 1 s segments and the Po of each segment was calculated. The data were idealised using model M2 so that the Po for both the full open state, shown in blue, and the merged sub-conductance states (red) was obtained. (B) Mean stability plot of the closed state, for n=17 single-channel recordings at +30 mV. As in (A), the average Po for the full and sub states was computed over time using 1 s long segments. The probability of the closed state was then computed as 1 minus the Po in full and sub states. The mean pClosed across 17 recordings, of length greater than 30 s, is plotted as a black line. The shaded region indicates the region of +/- SEM from the mean. There are no significant signs of inactivation over this time period. We observed similar results for other voltages, as well as over longer periods of time (up to 100 s, although fewer recordings of this length were available).

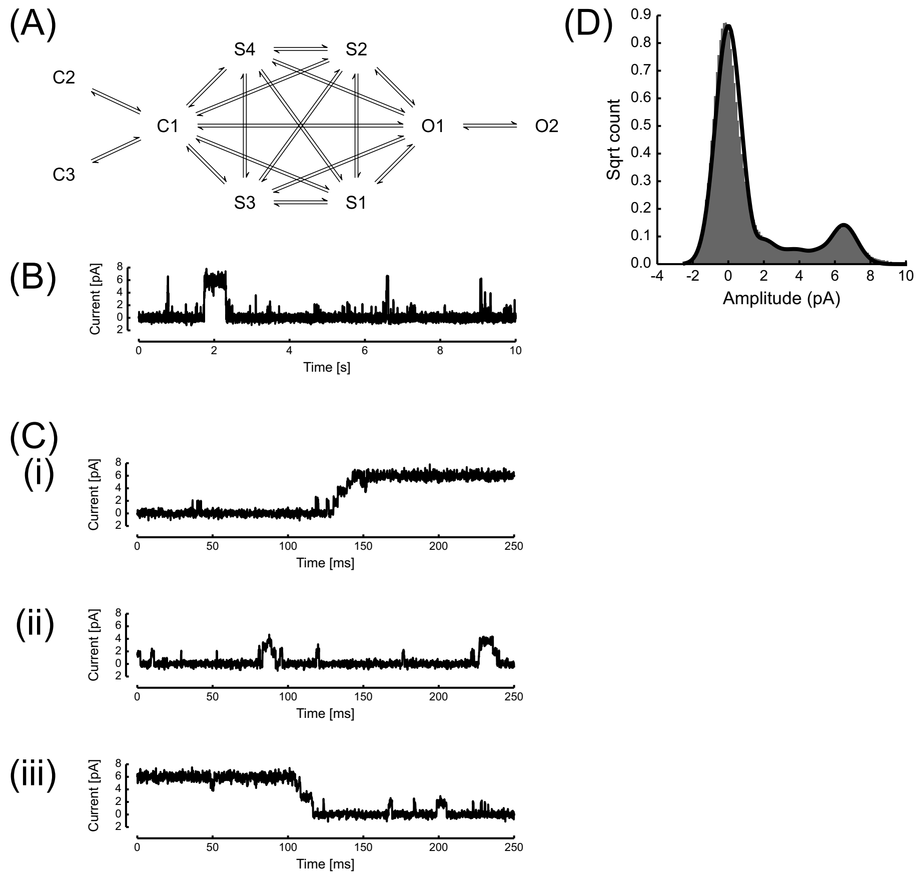

Figure S3. Model M1-adapted (adapted to include multiple full open and closed states) and typical examples of simulated data

(A) M1-adapted model topology, prior to removal of infrequent transitions. The four specified sub-conductance states in M1 were maintained in an all-to-all connected configuration with closed state C1 and fully open state O1. The closed states C2 and C3 and open state O2 were added to reflect the minimum number of time constants required to describe the open and closed lifetime distributions. Rates are omitted for clarity and are summarised in Table S4. (B) A typical example of 10 s of simulated data illustrating brief sub-conductance events that do not reach the full open level plus a typical opening burst to the full open state that lasts for several hundred milliseconds. (C) Simulated openings shown on an expanded time-scale to show the typical sub-conductance state gating that precedes the full opening burst (i), sub-conductance gating that does not transition into the fully open state (ii), and sub-conductance gating as the channel closes from the fully open state (iii). (D) Amplitude histogram of the simulated data (black line), using rate constants obtained from the same single experimental recording as in Fig. S4, S5, S6, S7 (iii), overlaid onto the current amplitude distribution of the corresponding experimental recording (grey bars). Sample counts plotted as square root.

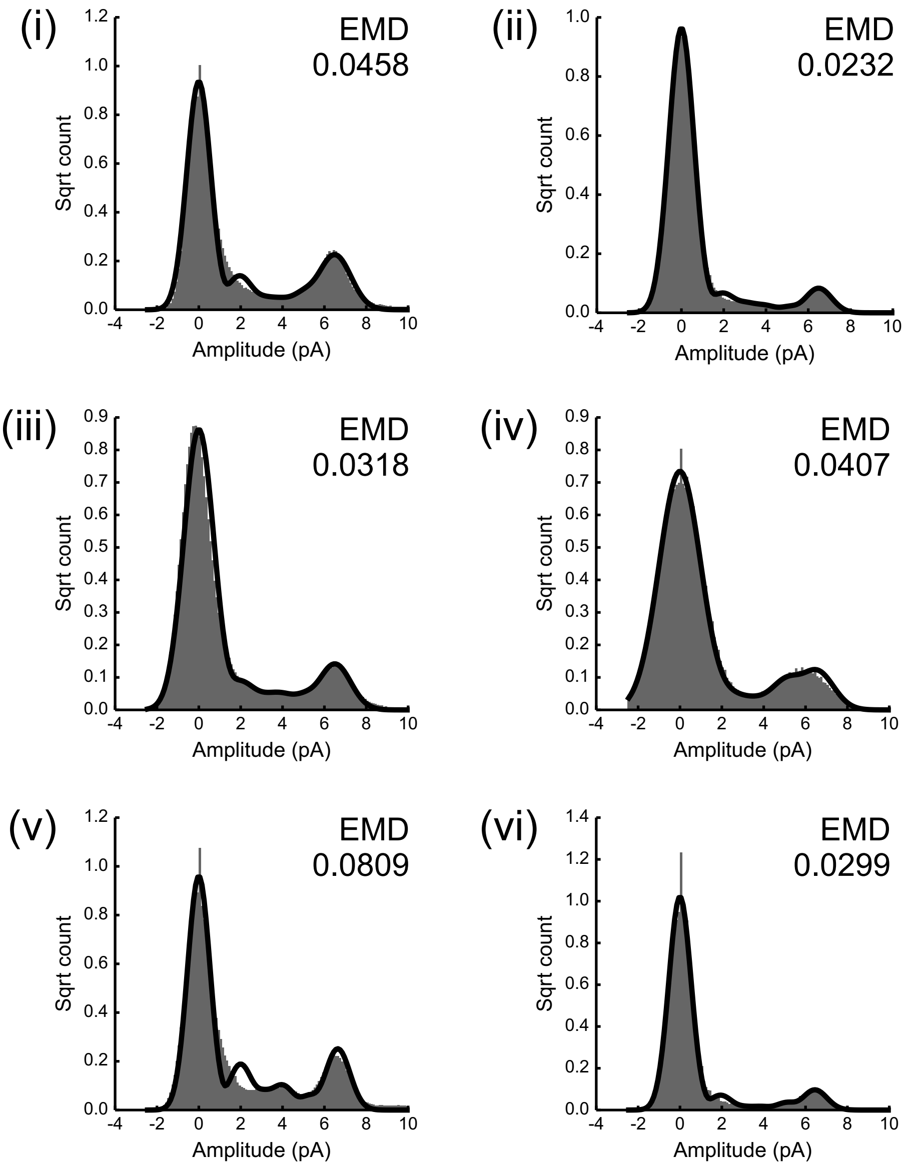

Figure S4. Amplitude histograms for individual experimental recordings, with corresponding simulations of model M1-adapted

Amplitude distributions of experimental data (grey bars) are shown for recordings of six representative voltage-dependent single channels, labelled (i) to (vi). For each experimental recording an individual set of kinetic rates was obtained for model M1-adapted, and amplitude histograms of the corresponding model simulations (black lines) are overlaid on each experimental histogram. Sample counts are plotted as square root. For each experimental recording, the EMD between the experimentally derived histogram and the corresponding model prediction is shown (Also see table S3). Note that amplitude histogram (iii) is the histogram shown in Fig. S3 (D).

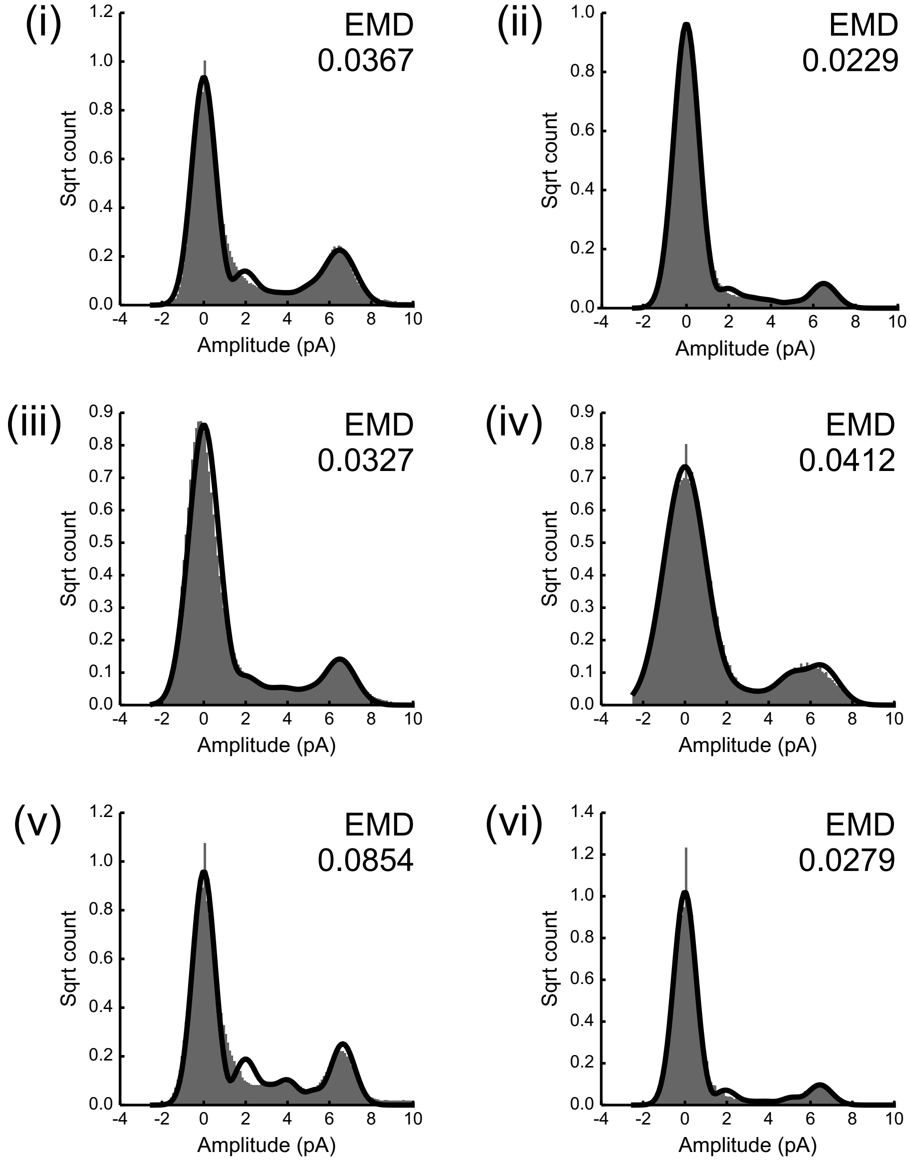

Figure S5. Amplitude histograms for individual experimental recordings, with corresponding simulations of model M1-reduced

Amplitude distributions of experimental data (grey bars) are shown for recordings of six representative voltage-dependent single channels, labelled (i) to (vi). For each experimental recording an individual set of kinetic rates was obtained for model M1-reduced, and amplitude histograms of the corresponding model simulations (black lines) are overlaid on each experimental histogram. Sample counts are plotted as square root. For each experimental recording, the EMD between the experimentally derived histogram and the corresponding model prediction is shown (also see table S3).

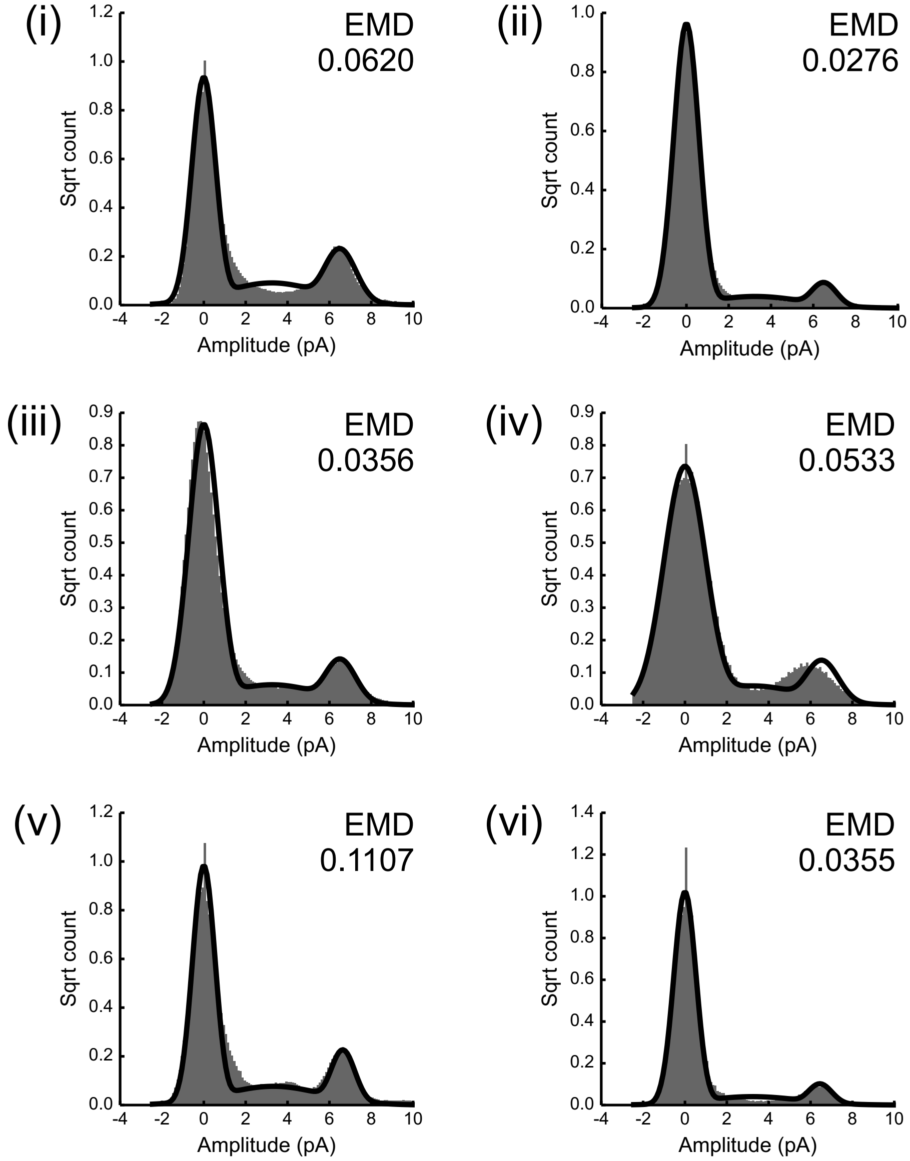

Figure S6. Amplitude histograms for individual experimental recordings, with corresponding simulations of model M2-adapted

Amplitude distributions of experimental data (grey bars) are shown for recordings of six representative voltage-dependent single channels, labelled (i) to (vi). For each experimental recording an individual set of kinetic rates was obtained for model M2-adapted, and amplitude histograms of the corresponding model simulations (black lines) are overlaid on each experimental histogram. Sample counts are plotted as square root. For each experimental recording, the EMD between the experimentally derived histogram and the corresponding model prediction is shown (also see Table S3).

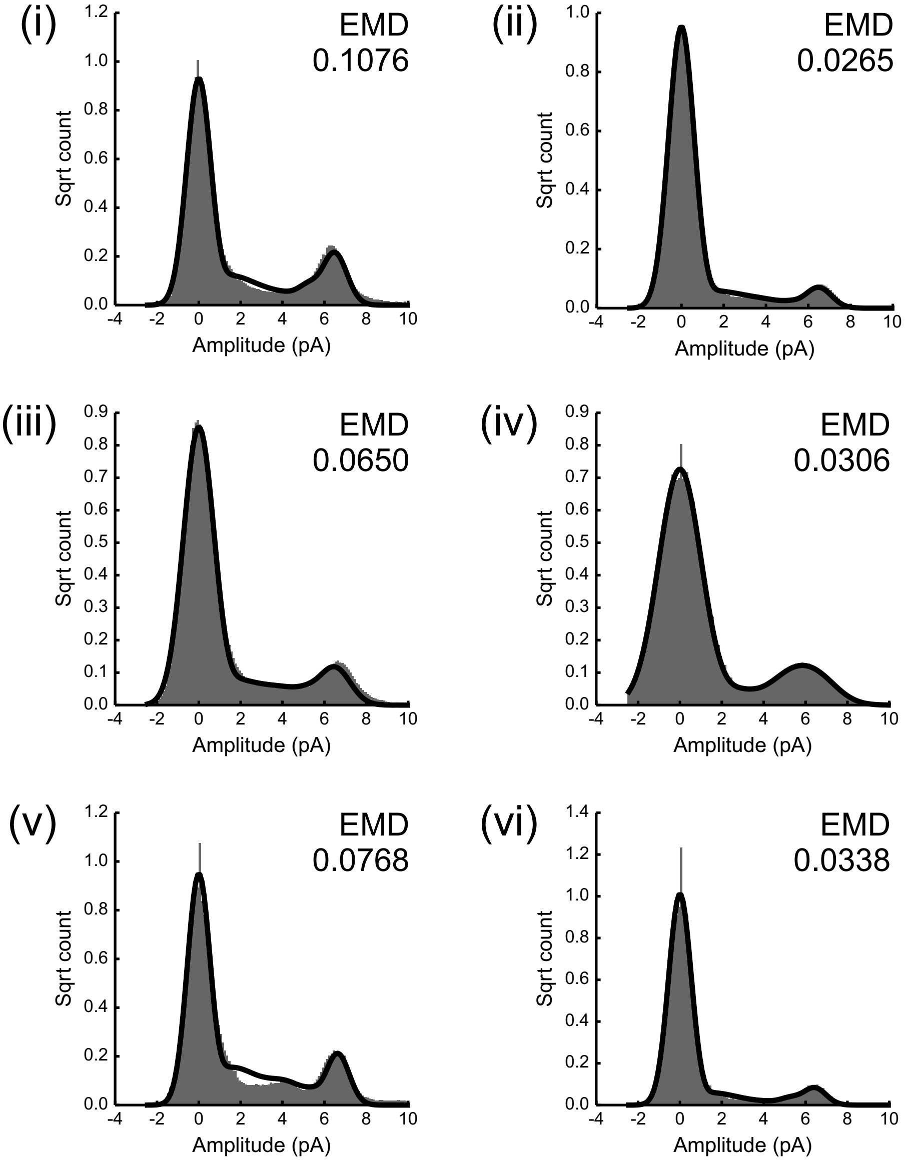

Figure S7. Amplitude histograms for individual experimental recordings, with corresponding simulations of the candidate fast-gating model

Amplitude distributions of experimental data (grey bars) are shown for recordings of six representative voltage-dependent single channels, labelled (i) to (vi). For each experimental recording an individual set of kinetic rates was obtained for the fast-gating model (corresponding to rates in M1-reduced, as discussed in the main material), and amplitude histograms of the corresponding model simulations (black lines) are overlaid on each experimental histogram. Sample counts are plotted as square root. For each experimental recording, the EMD between the experimentally derived histogram and the corresponding model prediction is shown (also see table S3).

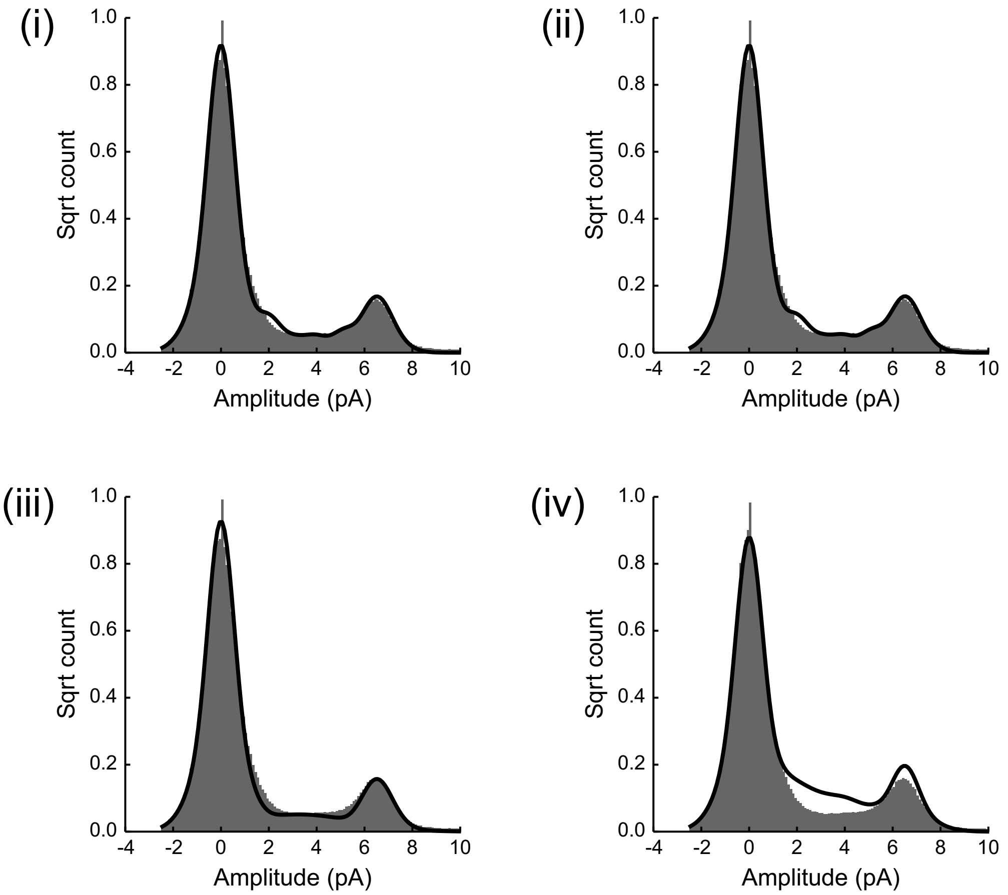

Figure S8. Mean amplitude histograms of model simulations with rates fitted globally to experimental data

The average outputs of the four kinetic models, using globally fitted rates to the six representative experimental recordings at +30 mV that were used throughout EMD analysis, are shown as simulated amplitude histograms (black lines) compared to mean amplitude histograms derived from the experimental data (grey bars). The kinetic models used were: (i) M1-adapted (Fig. S3), (ii) M1-reduced (Fig. 6A), (iii) M2-adapted (Fig. 6B)) and (iv) Fast-gating model (Fig. 6C).

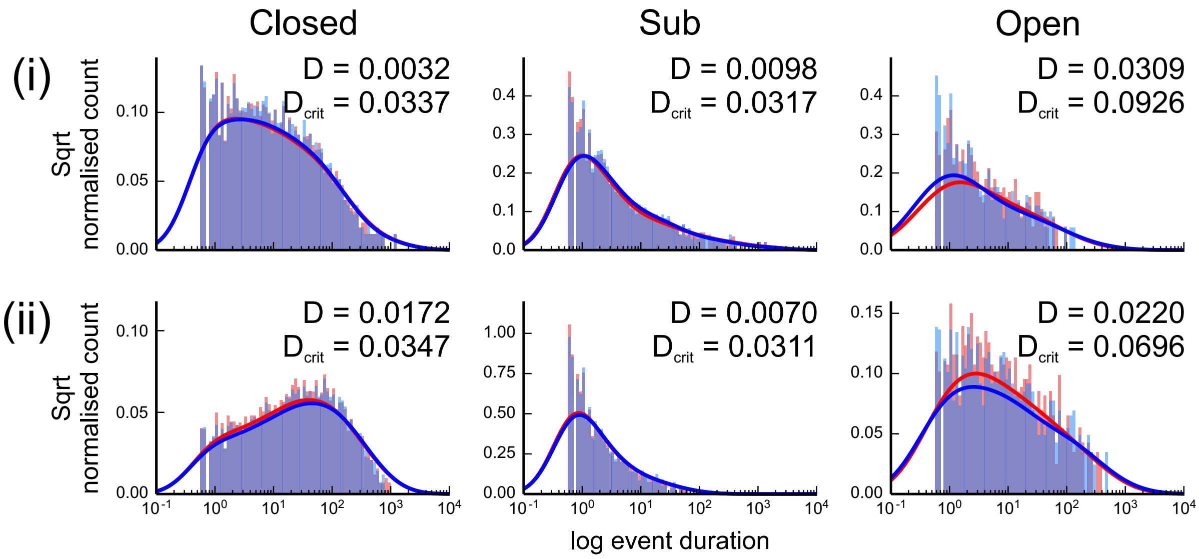

Figure S9. Comparison of lifetime distributions arising from idealisation in QuB with M1 or M2 using the kernel density method

Lifetime histograms are plotted for (left to right) the closed, sub-conductance open, and full open states from idealisations with M1 (orange bars) or M2 (blue bars) for two typical voltage-dependent channels ((i) and (ii)). Solid lines show the smoothed lifetime distribution estimated using the kernel density method (4), for M1 (red line) and M2 (blue line).

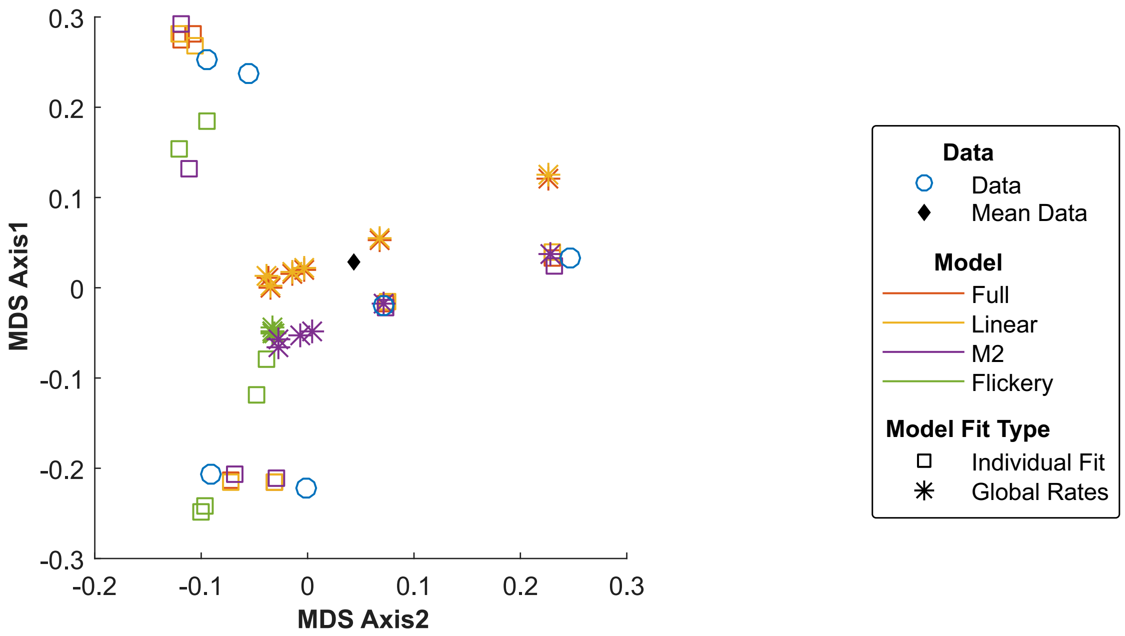

Figure S10. Application of multidimensional scaling to EMDs between models and data

The first two components of the configuration matrix after application of classic multidimensional scaling to EMDs between all model fits and data. Plot shows points corresponding to six representative experimental recordings (blue circles), the point corresponding to the mean distribution of experimental data (black diamond), and points corresponding to the amplitude distributions generated by various model fits. Symbols represent the manner in which a model's rates were fitted to data (squares: individually fitted rates; stars: globally fitted rates), and colours represent the model structure (red – full M1; yellow – reduced linear M1; purple – M2; Green – fast gating model).

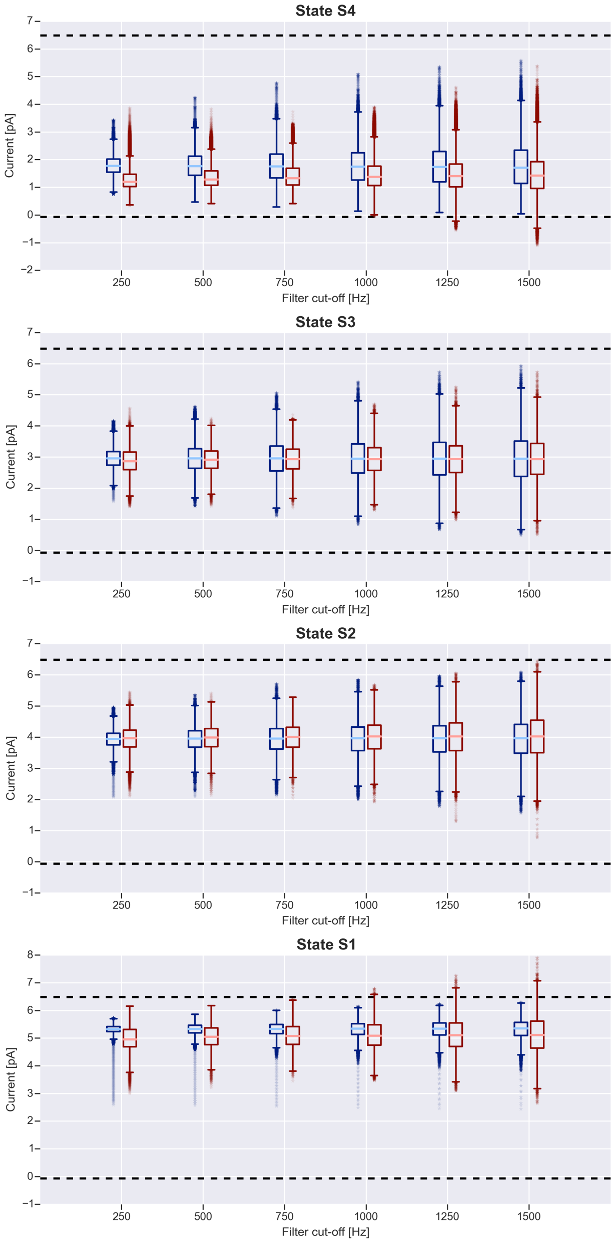

Figure S11. Comparison of filtering effects on experimentally observed sub-states, and sub-states generated by the fast-gating model.

A representative comparison of sub-state noise observed in a single experimental recording (red) to noise resulting from filtering applied to sub-states in the fast gating model (blue) is shown. The method in which this analysis was generated is discussed above. For each sub-state, data from the model are plotted in blue and experimental data are plotted in red. Boxes indicate the upper and lower quartiles of the data points at each filtering threshold. Pale lines across the centre of each box indicate the median current amplitude for a given state at a given filtering level. Whiskers extend from each box to 1.5 times the interquartile range, to indicate the approximate spread of data. Outliers are plotted as stars. Black dashed lines indicate the mean amplitude of the closed state (~0 pA), and the fully open state (~6.5 pA).

Supporting Tables

Table S1. Lifetime analysis for full open and closed states of single voltage-dependent channels

Probability density functions (pdfs) were fitted to the lifetime histograms of full open and closed states for 8 representative voltage-dependent single channels at +30 mV. The corresponding time constants and areas are tabulated for each channel. There were not enough events at -30 mV for lifetime analysis.

Closed State

Tau 1 (ms)	Area 1	Tau 2 (ms)	Area 2	Tau 3 (ms)	Area 3	Tau 4 (ms)	Area 4
1.08	13.2 %	31.95	26.5 %	231.82	60.3 %		
2.23	46.7 %	31.38	35.9 %	437.30	17.4 %		
0.80	38.5 %	10.00	42.3 %	138.99	19.3 %		
0.84	40.9 %	09.51	29.7 %	775.25	29.3 %		
0.70	12.0 %	7.87	10.4 %	81.59	40.7 %	176.13	36.8 %
0.92	13.5 %	7.54	18.5 %	38.17	43.5 %	104.58	24.5 %
0.50	21.6%	8.83	28.3%	62.42	30.5%	623.06	19.6%
1.14	29.0%	10.42	34.0%	60.55	20.5%	1122.75	16.4%

Full Open State

Tau 1 (ms)	Area 1	Tau 2 (ms)	Area 2	Tau 3 (ms)	Area 3
1.92	100.0 %				
1.97	30.3%	46.41	69.7%		
3.76	38.9%	149.88	61.1%		
0.95	31.0 %	10.85	69.0 %		
1.36	36.9 %	19.35	63.1 %		
0.92	40.8 %	16.52	59.2 %		
1.24	19.9 %	20.12	71.2 %	82.86	8.9 %
1.34	50.5 %	8.78	35.5 %	61.36	14.0 %

Table S2. Observed direct transitions between the closed and full open state

The total number of detected transitions across 8 experiments, where only a single channel is gating in the bilayer, when idealised with M1 or M2 are shown. The number of direct transitions between open and closed states (without apparent intermediate sub-conductance state transitions) is also shown. Only approximately 0.01% of all transitions were potentially direct openings or closings (passing through no intermediate sub-conductance states).

	Total number of transitions	Direct open-closed transitions	Percentage of total
**Model M1**	90839	12	0.013 %
**Model M2**	63538	7	0.011 %

Table S3. Comparison of experimentally observed mean single-channel currents with simulated mean currents derived from the various models

QuB was used to obtain the mean current (combined full open and sub-conductance state currents) for each of six representative voltage-dependent channels at +30 mV. The mean value of these currents across the six experiments was 0.289 pA. Simulated data from each of the four model types indicated in the first column was obtained. All models were adapted to incorporate the 3 closed and 2 full open states derived from lifetime analysis. Simulated recordings were obtained for each model using both individually and globally fitted rates to the six voltage-dependent channels, and the mean current was measured from the resulting amplitude distributions by computing their centroids (as described above) and is given in column 1. The second column gives the difference between the predicted model current and the mean experimental current (0.289 pA) expressed both as an absolute current magnitude (pA) and as a percentage of the experimental mean current.

To further characterise model performance, we used the Earth Movers Distance (EMD), to provide a measure of the difference (distance) between experimental and simulated amplitude distributions. This corresponds to the 'work' (total change in area) required to change the model distribution to exactly match experimental data. First, for each of the six experimental recordings, the mean EMD against all five other recordings was computed. The EMD between the six experimental recordings ranged from 0.355 to 0.417, with a mean value of 0.359. Secondly, the EMD between simulated amplitude distributions and experimental recordings was computed, and is shown in the rightmost column of this table. EMD for simulated versus experimentally derived amplitude distributions is much lower than that computed within the experimental data (0.359). This means that the amount of 'work' required to transform simulated amplitude distributions to those of corresponding experimental recordings is, on average, lower than that required to transform the amplitude distribution of one experimental recording to that of another. Therefore current amplitude distributions obtained through simulation of our models fall well within the range of variation that would be expected in our data.

	Model	Mean model current (pA)	Mean model current – mean experimental current	Mean model EMD
**Individually fitted rates**	M1-Adapted	0.2917	0.0030 (1.04 %)	0.0421
	Reduced, Linear M1	0.2909	0.0022 (0.77 %)	0.0411
	M2-Adapted	0.2692	-0.0194 (-6.73 %)	0.0541
	Fast-Gating	0.2498	-0.0388 (-13.45 %)	0.0567
**Globally fitted rates**	M1-Adapted	0.2924	0.0038 (1.31 %)	0.1778
	Reduced, Linear M1	0.2946	0.0059 (2.06 %)	0.1783
	M2-Adapted	0.2262	-0.0625 (-21.66 %)	0.1711
	Fast-Gating	0.2554	-0.0333 (-11.53 %)	0.2493

Table S4. Transition rates for model M1-adapted

The transition rates for the model shown in Fig. S3 (M1-adapted to include multiple closed and full open states) were obtained by simultaneous global fit of the data from n=8 voltage-dependent experimental recordings in QuB. The rows in the table give the rates out of a given Markov state and the columns give the rates entering a state. Zero rates (non-existent connections) are omitted for clarity. Those rates that correspond to transitions which either do not occur or have an extremely low probability of occurring across all channels, are shown in blue text. Rates are in units of sec^{-1}.

					**To**				
		**C1**	**C2**	**C3**	**S4**	**S3**	**S2**	**S1**	**O1**	**O2**
	**C1**		125	11.2	191	0.44	6.1 E-22	1.6 E-29	8.3 E-11	
	**C2**	12.7								
	**C3**	1.77								
	**S4**	1228				58.4	23	5.89	0.37	
**From**	**S3**	13.9			317		149	52.9	0.52	
	**S2**	8.9 E-38			151	195		163	40.7	
	**S1**	6.3			1.1 E-15	66.6	172		538	
	**O1**	5.3 E-19			1.98	9.3 E-7	9.04	112		70.1
	**O2**								31.4	
